# Mendelian randomization implies no direct causal association between leukocyte telomere length and amyotrophic lateral sclerosis

**DOI:** 10.1038/s41598-020-68848-9

**Published:** 2020-07-22

**Authors:** Yixin Gao, Ting Wang, Xinghao Yu, International FTD-Genomics Consortium (IFGC), Raffaele Ferrari, Dena G. Hernandez, Michael A. Nalls, Jonathan D. Rohrer, Adaikalavan Ramasamy, John B. J. Kwok, Carol Dobson-Stone, William S. Brooks, Peter R. Schofield, Glenda M. Halliday, John R. Hodges, Olivier Piguet, Lauren Bartley, Elizabeth Thompson, Eric Haan, Isabel Hernández, Agustín Ruiz, Mercè Boada, Barbara Borroni, Alessandro Padovani, Carlos Cruchaga, Nigel J. Cairns, Luisa Benussi, Giuliano Binetti, Roberta Ghidoni, Gianluigi Forloni, Diego Albani, Daniela Galimberti, Chiara Fenoglio, Maria Serpente, Elio Scarpini, Jordi Clarimón, Alberto Lleó, Rafael Blesa, Maria Landqvist Waldö, Karin Nilsson, Christer Nilsson, Ian R. A. Mackenzie, Ging-Yuek R. Hsiung, David M. A. Mann, Jordan Grafman, Christopher M. Morris, Johannes Attems, Timothy D. Griffiths, Ian G. McKeith, Alan J. Thomas, Pietro Pietrini, Edward D. Huey, Eric M. Wassermann, Atik Baborie, Evelyn Jaros, Michael C. Tierney, Pau Pastor, Cristina Razquin, Sara Ortega-Cubero, Elena Alonso, Robert Perneczky, Janine Diehl-Schmid, Panagiotis Alexopoulos, Alexander Kurz, Innocenzo Rainero, Elisa Rubino, Lorenzo Pinessi, Ekaterina Rogaeva, Peter St George-Hyslop, Giacomina Rossi, Fabrizio Tagliavini, Giorgio Giaccone, James B. Rowe, Johannes C. M. Schlachetzki, James Uphill, John Collinge, Simon Mead, Adrian Danek, Vivianna M. Van Deerlin, Murray Grossman, John Q. Trojanowski, Julie van der Zee, Marc Cruts, Christine Van Broeckhoven, Stefano F. Cappa, Isabelle Leber, Didier Hannequin, Véronique Golfier, Martine Vercelletto, Alexis Brice, Benedetta Nacmias, Sandro Sorbi, Silvia Bagnoli, Irene Piaceri, Jørgen E. Nielsen, Lena E. Hjermind, Matthias Riemenschneider, Manuel Mayhaus, Bernd Ibach, Gilles Gasparoni, Sabrina Pichler, Wei Gu, Martin N. Rossor, Nick C. Fox, Jason D. Warren, Maria Grazia Spillantini, Huw R. Morris, Patrizia Rizzu, Peter Heutink, Julie S. Snowden, Sara Rollinson, Anna Richardson, Alexander Gerhard, Amalia C. Bruni, Raffaele Maletta, Francesca Frangipane, Chiara Cupidi, Livia Bernardi, Maria Anfossi, Maura Gallo, Maria Elena Conidi, Nicoletta Smirne, Rosa Rademakers, Matt Baker, Dennis W. Dickson, Neill R. Graff-Radford, Ronald C. Petersen, David Knopman, Keith A. Josephs, Bradley F. Boeve, Joseph E. Parisi, William W. Seeley, Bruce L. Miller, Anna M. Karydas, Howard Rosen, John C. van Swieten, Elise G. P. Dopper, Harro Seelaar, Yolande A. L. Pijnenburg, Philip Scheltens, Giancarlo Logroscino, Rosa Capozzo, Valeria Novelli, Annibale A. Puca, Massimo Franceschi, Alfredo Postiglione, Graziella Milan, Paolo Sorrentino, Mark Kristiansen, Huei-Hsin Chiang, Caroline Graff, Florence Pasquier, Adeline Rollin, Vincent Deramecourt, Thibaud Lebouvier, Dimitrios Kapogiannis, Luigi Ferrucci, Stuart Pickering-Brown, Andrew B. Singleton, John Hardy, Parastoo Momeni, Huashuo Zhao, Ping Zeng

**Affiliations:** 1 Department of Epidemiology and Biostatistics, School of Public Health, Xuzhou Medical University, Xuzhou, 221004 Jiangsu People’s Republic of China; 2 Center for Medical Statistics and Data Analysis, School of Public Health, Xuzhou Medical University, Xuzhou, 221004 Jiangsu People’s Republic of China; 3 Department of Molecular Neuroscience, UCL, Russell Square House, 9-12 Russell Square House, London, WC1B 5EH UK; 4 Laboratory of Neurogenetics, National Institute on Aging, National Institutes of Health, Building 35, Room 1A215, 35 Convent Drive, Bethesda, MD 20892 USA; 5 Reta Lila Weston Research Laboratories, Department of Molecular Neuroscience, UCL Institute of Neurology, Queen Square, London, WC1N 3BG UK; 6 Department of Neurodegenerative Disease, Dementia Research Centre, UCL Institute of Neurology, Queen Square, London, WC1N 3BG UK; 7 Department of Medical and Molecular Genetics, King’s College London Tower Wing, Guy’s Hospital, London, SE1 9RT UK; 8 The Jenner Institute, University of Oxford, Roosevelt Drive, Oxford, OX3 7BQ UK; 9 Neuroscience Research Australia, Sydney, NSW 2031 Australia; 10 School of Medical Sciences, University of New South Wales, Sydney, NSW 2052 Australia; 11 Prince of Wales Clinical School, University of New South Wales, Sydney, NSW 2052 Australia; 12 South Australian Clinical Genetics Service, SA Pathology (at Women’s and Children’s Hospital), North Adelaide, SA 5006 Australia; 13 Department of Paediatrics, University of Adelaide, Adelaide, SA 5000 Australia; 14 Research Center and Memory Clinic of Fundació ACE, Institut Català de Neurociències Aplicades, Barcelona, Spain; 15 Neurology Clinic, University of Brescia, Brescia, Italy; 16 Department of Psychiatry, Washington University, St. Louis, MO USA; 17 Hope Center, Washington University School of Medicine, St. Louis, MO USA; 18 Department of Pathology and Immunology, Washington University, St. Louis, MO USA; 19 Molecular Markers Laboratory, IRCCS Istituto Centro San Giovanni di Dio Fatebenefratelli, Brescia, Italy; 20 MAC Memory Clinic, IRCCS Istituto Centro San Giovanni di Dio Fatebenefratelli, Brescia, Italy; 21 Biology of Neurodegenerative Disorders, IRCCS Istituto di Ricerche Farmacologiche, “Mario Negri”, Milan, Italy; 22 University of Milan, Milan, Italy; 23 Fondazione Cà Granda, IRCCS Ospedale Maggiore Policlinico, via F. Sforza 35, 20122 Milan, Italy; 24 Memory Unit, Neurology Department and Sant Pau Biomedical Research Institute, Hospital de la Santa Creu i Sant Pau, Universitat Autònoma de Barcelona, Barcelona, Spain; 25 Center for Networker Biomedical Research in Neurodegenerative Diseases (CIBERNED), Madrid, Spain; 26 Unit of Geriatric Psychiatry, Department of Clinical Sciences, Lund University, Lund, Sweden; 27 Clinical Memory Research Unit, Department of Clinical Sciences, Lund University, Lund, Sweden; 28 Department of Pathology and Laboratory Medicine, University of British Columbia, Vancouver, Canada; 29 Division of Neurology, University of British Columbia, Vancouver, Canada; 30 Institute of Brain, Behaviour and Mental Health, University of Manchester, Salford Royal Hospital, Stott Lane, Salford, M6 8HD UK; 31 Departments of Physical Medicine and Rehabilitation, Psychiatry, and Cognitive Neurology and Alzheimer’s Disease Center, Rehabilitation Institute of Chicago, Feinberg School of Medicine, Northwestern University, Chicago, USA; 32 Department of Psychology, Weinberg College of Arts and Sciences, Northwestern University, Chicago, USA; 33 Newcastle Brain Tissue Resource, Institute for Ageing, Newcastle University, Newcastle upon Tyne, NE4 5PL UK; 34 Institute of Neuroscience and Institute for Ageing, Campus for Ageing and Vitality, Newcastle University, Newcastle upon Tyne, NE4 5PL UK; 35 Institute of Neuroscience, Newcastle University Medical School, Framlington Place, Newcastle upon Tyne, NE2 4HH UK; 36 IMT School for Advanced Studies, Lucca, Lucca, Italy; 37 Departments of Psychiatry and Neurology, Taub Institute, Columbia University, 630 West 168th Street, New York, NY 10032 USA; 38 Behavioral Neurology Unit, National Insititute of Neurological Disorders and Stroke, National Insititutes of Health, 10 Center DR MSC 1440, Bethesda, MD 20892-1440 USA; 39 Department of Laboratory Medicine and Pathology, Walter Mackenzie Health Sciences Centre, University of Alberta Edmonton, 8440 - 112 St, Alberta, T6G 2B7 Canada; 40 Institute for Ageing and Health, Campus for Ageing and Vitality, Newcastle University, Newcastle upon Tyne, NE4 5PL UK; 41 Neurogenetics Laboratory, Division of Neurosciences, Center for Applied Medical Research, Universidad de Navarra, Pamplona, Spain; 42 Department of Neurology, Clínica Universidad de Navarra, University of Navarra School of Medicine, Pamplona, Spain; 43 Neuroepidemiology and Ageing Research Unit, School of Public Health, Faculty of Medicine, The Imperial College of Science, Technology and Medicine, London, W6 8RP UK; 44 West London Cognitive Disorders Treatment and Research Unit, West London Mental Health Trust, London, TW8 8 DS UK; 45 Department of Psychiatry and Psychotherapy, Technische Universität München, 81675 Munich, Germany; 46 Neurology I, Department of Neuroscience, University of Torino, Turin, Italy; 47 A.O. Città della Salute e della Scienza di Torino, Turin, Italy; 48 Tanz Centre for Research in Neurodegenerative Diseases, University of Toronto, 60 Leonard Street, Toronto, ON M5T 2S8 Canada; 49 Department of Clinical Neurosciences, Cambridge Institute for Medical Research, University of Cambridge, Hills Road, Cambridge, CB2 0XY UK; 50 Division of Neurology V and Neuropathology, Fondazione IRCCS Istituto Neurologico Carlo Besta, 20133 Milan, Italy; 51 Department of Clinical Neurosciences, Cambridge University, Cambridge, CB2 0SZ UK; 52 MRC Cognition and Brain Sciences Unit, Cambridge, CB2 7EF UK; 53 Behavioural and Clinical Neuroscience Institute, Cambridge, CB2 3EB UK; 54 Department of Cellular and Molecular Medicine, University of California San Diego, 9500 Gilman Drive, La Jolla, CA 92093 USA; 55 MRC Prion Unit, Department of Neurodegenerative Disease, UCL Institute of Neurology, Queen Square House, Queen Square, London, WC1N 3BG UK; 56 Neurologische Klinik und Poliklinik, Ludwig-Maximilians-Universität, Munich, Germany; 57 German Center for Neurodegenerative Diseases (DZNE), Munich, Germany; 58 Department of Pathology and Laboratory Medicine, University of Pennsylvania Perelman School of Medicine, Philadelphia, PA USA; 59 Department of Neurology and Penn Frontotemporal Degeneration Center, University of Pennsylvania Perelman School of Medicine, Philadelphia, PA USA; 60 Neurodegenerative Brain Diseases Group, Department of Molecular Genetics, VIB, Antwerp, Belgium; 61 Laboratory of Neurogenetics, Institute Born-Bunge, University of Antwerp, Antwerp, Belgium; 62 Neurorehabilitation Unit, Department of Clinical Neuroscience, Vita-Salute University and San Raffaele Scientific Institute, Milan, Italy; 63 Inserm, UMR_S975, CRICM, 75013 Paris, France; 64 UPMC Univ Paris 06, UMR_S975, 75013 Paris, France; 65 CNRS UMR 7225, 75013 Paris, France; 66 Département de neurologie-centre de références des démences rares, AP-HP, Hôpital de la Salpêtrière, 75013 Paris, France; 67 Service de Neurologie, Inserm U1079, CNR-MAJ, Rouen University Hospital, Rouen, France; 68 Service de Neurologie, CH Saint Brieuc, Saint Brieuc, France; 69 Service de Neurologie, CHU Nantes, Nantes, France; 70 Department of Neurosciences, Psychology, Drug Research and Child Health (NEUROFARBA), University of Florence, Florence, Italy; 71 Department of Neurosciences, Psychology, Drug Research and Child Health (NEUROFARBA), University of Florence and IRCCS “Don Carlo Gnocchi” Firenze, Florence, Italy; 72 Danish Dementia Research Centre, Neurogenetics Clinic, Department of Neurology, Rigshospitalet, Copenhagen University Hospital, Copenhagen, Denmark; 73 Department of Cellular and Molecular Medicine, Section of Neurogenetics, The Panum Institute, University of Copenhagen, Copenhagen, Denmark; 74 Department for Psychiatry and Psychotherapy, Saarland University Hospital, Kirrberger Str.1, Bld.90, 66421 Homburg/Saar, Germany; 75 Laboratory for Neurogenetics, Saarland University, Kirrberger Str.1, Bld.90, 66421 Homburg/Saar, Germany; 76 Department of Psychiatry, Psychotherapy and Psychosomatics, University Regensburg, Universitätsstr. 84, 93053 Regensburg, Germany; 77 Luxembourg Centre For Systems Biomedicine (LCSB), University of Luxembourg, 7, Avenue des Hauts-Fourneaux, 4362 Esch-sur-Alzette, Luxembourg; 78 Department of Clinical Neurosciences, John Van Geest Brain Repair Centre, University of Cambridge, Forvie Site, Robinson Way, Cambridge, CB2 0PY UK; 79 German Center for Neurodegenerative Diseases-Tübingen, Otfried Muellerstrasse 23, 72076 Tuebingen, Germany; 80 Faculty of Medical and Human Sciences, Institute of Brain, Behaviour and Mental Health, University of Manchester, Manchester, UK; 81 Salford Royal Foundation Trust, Faculty of Medical and Human Sciences, University of Manchester, Manchester, UK; 82 Institute of Brain, Behaviour and Mental Health, The University of Manchester, 27 Palatine Road, Withington, Manchester, M20 3LJ UK; 83 Regional Neurogenetic Centre, ASPCZ, Lamezia Terme, Italy; 84 Department of Neuroscience, Mayo Clinic Jacksonville, 4500 San Pablo Road, Jacksonville, FL 32224 USA; 85 Department of Neurology, Mayo Clinic Jacksonville, 4500 San Pablo Road, Jacksonville, FL 32224 USA; 86 Department of Neurology, Mayo Clinic Rochester, 2001st Street SW, Rochester, MN 5905 USA; 87 Department of Pathology, Mayo Clinic Rochester, 2001st Street SW, Rochester, MN 5905 USA; 88 Department of Neurology, University of California, Box 1207, San Francisco, CA 94143 USA; 89 Department of Neurology, Memory and Aging Center, University of California, San Francisco, CA 94158 USA; 90 Department of Neurology, Erasmus Medical Centre, Rotterdam, The Netherlands; 91 Department of Medical Genetics, VU University Medical Centre, Amsterdam, The Netherlands; 92 Alzheimer Centre and Department of Neurology, VU University Medical Centre, Amsterdam, The Netherlands; 93 Department of Basic Medical Sciences, Neurosciences and Sense Organs of the “Aldo Moro” University of Bari, Bari, Italy; 94 Medical Genetics Unit, Fondazione Policlinico Universitario A. Gemelli, Rome, Italy; 95 Cardiovascular Research Unit, IRCCS Multimedica, Milan, Italy; 96 Department of Medicine and Surgery, University of Salerno, Baronissi, SA Italy; 97 Neurology Department, IRCCS Multimedica, Milan, Italy; 98 Department of Clinical Medicine and Surgery, University of Naples Federico II, Naples, Italy; 99 Geriatric Center Frullone- ASL Napoli 1 Centro, Naples, Italy; 100 UCL Genomics, Institute of Child Health (ICH), UCL, London, UK; 101 Dept NVS, Alzheimer Research Center, Karolinska Institutet, Novum, 141 57 Stockholm, Sweden; 102 Department of Geriatric Medicine, Genetics Unit, M51, Karolinska University Hospital, 14186 Stockholm, Sweden; 103 Univ Lille, Inserm 1171, DISTALZ, CHU, 59000 Lille, France; 104 National Institute on Aging (NIA/NIH), 3001 S. Hanover St, NM 531, Baltimore, MD 21230 USA; 105 Clinical Research Branch, National Institute on Aging, Baltimore, MD USA; 106 Laboratory of Neurogenetics, Department of Internal Medicine, Texas Tech University Health Science Center, 4th street, Lubbock, TX 79430 USA

**Keywords:** Computational biology and bioinformatics, Genetics, Medical research, Neurology, Risk factors

## Abstract

We employed Mendelian randomization (MR) to evaluate the causal relationship between leukocyte telomere length (LTL) and amyotrophic lateral sclerosis (ALS) with summary statistics from genome-wide association studies (*n* = ~ 38,000 for LTL and ~ 81,000 for ALS in the European population; *n* = ~ 23,000 for LTL and ~ 4,100 for ALS in the Asian population). We further evaluated mediation roles of lipids in the pathway from LTL to ALS. The odds ratio per standard deviation decrease of LTL on ALS was 1.10 (95% CI 0.93–1.31, *p* = 0.274) in the European population and 0.75 (95% CI 0.53–1.07, *p* = 0.116) in the Asian population. This null association was also detected between LTL and frontotemporal dementia in the European population. However, we found that an indirect effect of LTL on ALS might be mediated by low density lipoprotein (LDL) or total cholesterol (TC) in the European population. These results were robust against extensive sensitivity analyses. Overall, our MR study did not support the direct causal association between LTL and the ALS risk in neither population, but provided suggestive evidence for the mediation role of LDL or TC on the influence of LTL and ALS in the European population.

## Introduction

Amyotrophic lateral sclerosis (ALS) is an adult-onset fatal multisystem neurodegenerative disease, leading to substantial public health threat although it is relatively rare worldwide. However, the cause and pathogenesis underlying ALS mostly remains unknown, with few replicable and definitive risk factors and scarce drugs available^1–4^. The number of ALS cases is predicted to increase dramatically due to population aging in the coming years^5^, which would further aggravate the ALS-associated social and economic burden. Therefore, the identification of its risk factors can provide better understanding of ALS and has the potential to pave the way for therapeutic intervention.

In the past few years the role of telomere in various complex diseases has attracted much attention^6^. Progressive telomere shortening occurs in all dividing normal cells due to incomplete synthesis of DNA lagging-strand, oxidative damage and other factors, which ultimately leads to cellular growth arrest or apoptosis that is thought to be an initial proliferative barrier to tumor development in humans^7^. Indeed, recent studies suggested that leukocyte telomere length (LTL) was widely relevant to age-related diseases and disorders (e.g. many types of cancer and coronary heart disease)^8–11^. In particular, it was demonstrated that shorter LTL was associated with various neurodegenerative disorders. For example, a latest study showed LTL at baseline and 18 months was shorter in patients of Parkinson's disease (PD) compared to healthy controls^12^, although prior studies found nonsignificant association between LTL and PD (Table 1). In addition, telomere shortening was recognized as an indicator of progression for Alzheimer’s disease (AD) (Table 1).

**Table 1:** Estimated effect sizes of shorter LTL on neurodegenerative diseases in previous studies.

NDD	OR/HR (95% CI, *p*)	*N* (case/control)	Country	References
PD	0.70 (0.38–1.28, 0.246)	956/1,284	EUR and Asian	^74^
PD	0.91 (0.71–1.16, 0.450)	96/172	USA	^75^
PD	0.99 (0.77–1.27, 0.535)	131/115	Finland	^76^
PD	0.99 (0.88–1.12, 0.875)	408/809	USA	^77^
PD	1.30 (0.76–2.17, 0.340)	28/27	Japan	^78^
ALS	0.89 (0.68–1.16, 0.400)	6,100/7,125	EUR	^9^
ALS	0.92 (0.87–0.97, 0.008)	1,241/335	UK	^14^
AD	1.03 (1.01–1.05, 0.012)	71,880/383,378	EUR	^79^
AD	1.05 (1.01–1.09, 0.010)	71,880/383,378	EUR	^80^
AD	1.19 (1.02–1.41, 0.030)	17,008/37,154	EUR	^9^
AD	1.35 (1.12–1.67, 0.002)	25,580/48,466	EUR	^81^
AD	1.35 (1.11–1.67, 0.003)	25,580/48,466	EUR	^82^
AD	2.70 (1.69–4.17, 1.47E−05)	860/2,022	Multiethnic	^83^
Dementia	1.20 (1.00–1.47, 0.058)	190/1,469	Multiethnic	^84^
Dementia	5.26 (1.85–14.3, 0.002)	20/151	UK	^85^

*NDD* neurodegenerative disease, *PD* Parkinson’s disease, *ALS* amyotrophic lateral sclerosis, *AD* Alzheimer’s disease, *OR* odds ratio, *HR* hazard ratio, *CI* confidence internal, *p p* value, *N* sample size, *EUR* European.

However, the knowledge about the relationship between LTL and ALS is very limited. Previous studies proposed that telomerase inhibition could be a pathogenetic contributor to the neurodegeneration in ALS^13^. A recent study^14^, along with ALS animal models^15^, offered some evidence that shorter LTL likely decreased the risk of ALS (Table 1). However, it remains uncertain whether such association is causal or not. Because it is rather challenging to determinate causal relationship between LTL and ALS via observational studies or randomized controlled trials (RCT), in this study we resort to another novel statistical approach called Mendelian randomization (MR)^16,17^. Briefly, depending on single nucleotide polymorphisms (SNPs) as instrumental variables, MR can infer the causal association between an exposure (e.g. LTL) and an outcome (e.g. ALS)^17,18^. The basic idea behind MR is that the two alleles of a genetic variant are randomly allocated during the process of gamete formation under the Mendel’s law; such allocation is analogous to the randomization of subjects in RCT and hence has a powerful control for reverse causality and confounders^19^ (Supplementary Fig. S1). Furthermore, the recent success of large-scale genome-wide association studies (GWASs)^20–24^ allows us to choose appropriate SNPs as valid instrumental variables for a variety of exposures for causal inference in MR^25–27^.

In this study we aim to investigate whether there exists a causal association between LTL and the risk of ALS. To achieve such goal, we conducted the two-sample MR analysis with summary statistics publicly available from GWASs with ~ 38,000 individuals for LTL and ~ 81,000 individuals for ALS in the European population, and with ~ 23,000 individuals for LTL and ~ 4,100 individuals for ALS in the Asian population. Additionally, we further explored the mediation role of lipids in the relationship between LTL and ALS with network MR analysis given the evidence that blood lipids may be relevant to ALS.

## Materials and methods

### GWAS data sources for LTL, ALS and other relevant traits

We first obtained genetic data for LTL from the ENGAGE Telomere Consortium^21^, where a total of ~ 2.3 million SNPs for 37,684 individuals of European ancestry were contained after quality control (Supplementary Text). In this study LTL was measured as a continuous variable, and the linear additive regression was implemented for each genetic variant to detect the association with LTL^21^. A set of independent associated index SNPs (*p* < 5.00E−8) were selected as candidate instrumental variables for LTL. To minimize the pleiotropic bias of instruments, we applied a conservative manner^28^ that was previously undertaken in many MR studies^20,29–32^. Specifically, we would remove index SNPs that were located within 1 Mb of ALS-associated locus (Supplementary Table S1) and that may be potentially related to ALS if their Bonferroni-adjusted *p* values were less than 0.05. Finally, we reserved seven SNPs to serve as instrumental variables. To estimate the causal effect of LTL on ALS, we obtained summary statistics from the largest ALS GWAS that contained ~ 10 million SNPs on 80,610 European individuals (20,806 ALS cases and 59,804 controls)^20^ (https://als.umassmed.edu/). The summary statistics (e.g. marginal effect size, standard error and effect allele) of these instruments are shown in Table 2.

**Table 2:** Summary information of instrumental variables for LTL and ALS in the European population.

SNP	GENE	CHR	BP	A1/A2	LTL				ALS				PVE	*F*
					BETA	SE	*p*	*N*	BETA	SE	*p*	*N*		
rs11125529	*TERT*	2	54,329,370	C/A	− 0.056	0.010	4.48E−08	37,653	− 0.007	0.020	0.730	80,610	8.32E−04	31.4
rs10936599	*TERC*	3	170,974,795	T/C	− 0.079	0.008	2.54E−31	37,669	0.003	0.016	0.839	80,610	3.89E−03	147.0
rs7675998	*ZNF208*	4	164,227,270	A/G	− 0.074	0.009	4.35E−16	34,694	− 0.005	0.016	0.747	80,610	1.94E−03	67.6
rs2736100	*NAF1*	5	1,339,516	A/C	− 0.078	0.009	4.38E−19	25,842	0.010	0.014	0.493	80,610	2.90E−03	75.1
rs9420907	*ACYP2*	10	105,666,455	A/C	− 0.069	0.010	6.90E−11	37,653	0.050	0.019	0.011	80,610	1.26E−03	47.6
rs8105767	*RTEL1*	19	22,007,281	A/G	− 0.048	0.008	1.11E−09	37,499	0.006	0.015	0.683	80,610	9.59E−04	36.0
rs755017	*OBFC1*	20	61,892,066	A/G	− 0.062	0.011	6.71E−09	37,113	− 0.005	0.022	0.831	80,610	8.55E−04	31.8

*SNP* the label of single-nucleotide polymorphism that served as instrumental variable, *CHR* chromosome, *BP* base position, *A1* effect allele, indicates the allele that is associated with shorter LTL, explaining why all the BETA estimates are negative, *A2* alternative allele, *BETA* SNP effect size, *SE* standard error of the SNP effect size, *p* and *N* are respectively the *p* value and sample size, *PVE* proportion of variance explained by the SNP (i.e. ${\text{PVE}}_{i} = (\hat{\beta }_{i}^{x} )^{2} /((\hat{\beta }_{i}^{x} )^{2} + {\text{var(}}\hat{\beta }_{i}^{x} ) \times N_{i} )$^86^, where $\hat{\beta }_{i}^{x}$ and ${\text{var(}}\hat{\beta }_{i}^{x} )$ are the estimated effect size and variance for instrument *i*; *F*: *F* statistic (i.e. $F_{i} = {\text{PVE}}_{i} {(}N_{i} - 1 - k{)/}(k - k \times {\text{PVE}}_{i} )$^87,88^, where *N*_*i*_ is the sample size for instrument *i* and *k* is the number of instruments). Both of PVE and *F* statistic are calculated to validate the issue of weak instruments.

In addition, since ALS and frontotemporal dementia (FTD) often represent a continuous disease spectrum with comorbidity in up to 50% cases, and share common genetic mechanisms^33–35^, we also explored the causal association between LTL and FTD with MR approaches (Table 3). We removed index SNPs that were associated with FTD^36^ and reserved six instruments as one instrument was missing in the FTD GWAS data set (Supplementary Tables S2-S3). Furthermore, we attempted to validate whether the identified relationship between LTL and ALS in the European population also holds in the Asian population. Therefore, we performed additional MR analyses with another two GWAS datasets in which both LTL^22^ and ALS^37^ were conducted on the Asian individuals (Supplementary Text). Note that, the two sets of index SNPs of LTL from the two populations share no common instruments (Table 2 and Supplementary Table S4).

**Table 3:** GWAS data sets used in our MR analysis in the present study.

Traits	Pop	*k*_1_/*k*_0_	*N* (case/control)	Data source
ALS	EUR		80,610 (20,806/59,804)	AVS^20^
HDL	EUR	85/87	93,561	GLGC^61^
LDL	EUR	78/78	89,138	GLGC^61^
TC	EUR	86/86	93,845	GLGC^61^
TG	EUR	53/54	90,263	GLGC^61^
LTL	EUR	7/7	37,684	ENGAGE^21^
FTD	EUR		12,928 (3,526/9,402)	IFGC^36^
HDL	EUR	79/87	93,561	GLGC^61^
LDL	EUR	66/78	89,138	GLGC^61^
TC	EUR	76/86	93,845	GLGC^61^
TG	EUR	47/54	90,263	GLGC^61^
LTL	EUR	6/7	37,684	ENGAGE^21^
ALS	Asian		4,084 (1,234/2,850)	Benyamin^37^
HDL	Asian	30/31	70,657	Kanai^89^
LDL	Asian	21/22	72,866	Kanai^89^
TC	Asian	31/32	128,305	Kanai^89^
TG	Asian	26/26	105,597	Kanai^89^
LTL	Asian	8/10	23,096	SCHS^22^

Here *k*_1_ is the final number of instruments employed in the analysis while *k*_0_ is the number of candidate instruments.

*ALS* amyotrophic lateral sclerosis, *FTD* frontotemporal dementia, *HDL* high density lipoprotein, *LDL* low density lipoprotein, *TC* total cholesterol, *TG* triglycerides, *LTL* leukocyte telomere length, *Pop* population, *EUR* European, *AVS* the ALS Variant Server, *IFGC* International FTD-Genomics Consortium, *GLGC* Global Lipids Genetics Consortium, *ENGAGE* European Network for Genetic and Genomic Epidemiology, *SCHS* Singapore Chinese Health Study.

We note that the ALS cases were sporadic and the European-ALS GWAS adjusted the effect of age in the association analysis (Supplementary Text). The latter indicates that the confounding effect due to age on the causal effect estimation was removed. In addition, given the fact that LTL would shorten progressively with age, to facilitate the explanation of our results, we thus made a sign transformation for effect sizes of those used instrumental variables so that the causal relationship corresponds to *shorter* LTL.

### Causal effect estimation via two-sample Mendelian randomization

We implemented the two-sample MR to estimate the causal effect of LTL on ALS via inverse-variance weighted (IVW) methods^38–41^ (Supplementary Text). We also employed the weighted median method^42^, likelihood-based approach^43^, leave-one-out (LOO) analysis^44^, MR-PRESSO test^45^ and MR-Egger regression^38,46^ as part of sensitivity analyses to validate the robustness of our results. As a supplementary analysis, we further implemented the generalized summary based Mendelian Randomization (GSMR) method^47^ by leveraging possible linkage disequilibrium among instruments, and applied the HEIDI-outlier approach to detect pleiotropic instrumental variables.

### Mediation analysis to explore the mediation effect of lipids between LTL and ALS/FTD

In our MR analysis, we attempted to provide deeper insight into the relationship between LTL and ALS/FTD by conducting mediation analysis although non-significant causal associations were identified in neither population. Because previous studies showed LTL was associated with blood lipid levels^48–52^ (as would be also confirmed by our results; see below for details), and because there existed evidence for potential causal associations between lipids and ALS^3,53,54^, we further investigated whether the effect of LTL on ALS/FTD might be mediated through lipids^55–59^ by implementing network MR analysis^60^ with the lipid trait (e.g. HDL, LDL, TC or TG)^61^ as mediator (Supplementary Fig. S2 and Supplementary Text). Besides LTL, in the network MR analysis each of lipids should also have a set of instrumental variables (Table 3). The details of selecting instrumental variables for lipids were described elsewhere^53^. To make the estimated causal effects comparable between the European and Asian populations, following prior work^53^ we unified the units of lipid in the two populations (Supplementary Text). The summary statistics of instruments for lipids are displayed in Supplementary Tables S5-S9.

## Results

### Causal effect of LTL on ALS and FTD

A total of seven instrumental variables of LTL were employed in the European population (Table 2). All the selected instruments collectively explain about 1.26% phenotypic variation of LTL and all the *F* statistics are above 10 (ranging from 31.4 to 147.0 with an average of 62.3) (Table 2), which rules out the possibility of weak instrument bias^28,39,62^. With the fixed-effects IVW method, we observe that the odds ratio (OR) per standard deviation (SD) decrease of LTL (~ 30 base pair per year) on ALS is 1.10 (95% confidence interval [CI] 0.93–1.31, *p* = 0.274) in the European population and 0.75 (95% CI 0.53–1.07, *p* = 0.116) in the Asian population (Table 4). We also fail to detect statistically significant causal relationship between LTL and FTD in the European population, with the OR per SD decrease of LTL on FTD estimated to be 0.81 (95% CI 0.44–1.48, *p* = 0.498) (Table 4).

**Table 4:** Association of LTL with the risk of ALS or FTD in the European and Asian populations.

Method	ALS-european	FTD-european	ALS-asian
	OR (95% CI, *p*)	OR (95% CI, *p*)	OR (95% CI, *p*)
IVW-random	1.10 (0.92–1.32, 0.284)	0.81 (0.44–1.48, 0.498)	0.75 (0.53–1.07, 0.116)
IVW-fixed	1.10 (0.93–1.31, 0.274)	0.81 (0.44–1.48, 0.498)	0.75 (0.53–1.07, 0.116)
MR-Egger	1.02 (0.32–3.29, 0.964)	0.40 (0.01–14.71, 0.516)	0.61 (0.24–1.56, 0.241)
Weighted Median	1.06 (0.85–1.32, 0.624)	0.73 (0.35–1.52, 0.400)	0.67 (0.43–1.05, 0.082)
Likelihood	1.10 (0.92–1.32, 0.290)	0.81 (0.44–1.48, 0.496)	0.75 (0.53–1.07, 0.115)
GSMR	1.10 (0.93–1.31, 0.274)	0.81 (0.44–1.48, 0.498)	0.73 (0.51–1.05, 0.086)^a^

The intercept of the MR-Egger regression is 0.006 (95% CI − 0.079–0.090, *p* = 0.872), 0.055 (95% CI − 0.214–0.323, *p* = 0.601) or 0.026 (95% CI − 0.076–0.128, *p* = 0.552), respectively.

^a^Seven instruments were finally employed because the genotype of rs41309367 on gene *RTEL1* was missing in the 1,000 Genomes Project.

**Figure 1:**
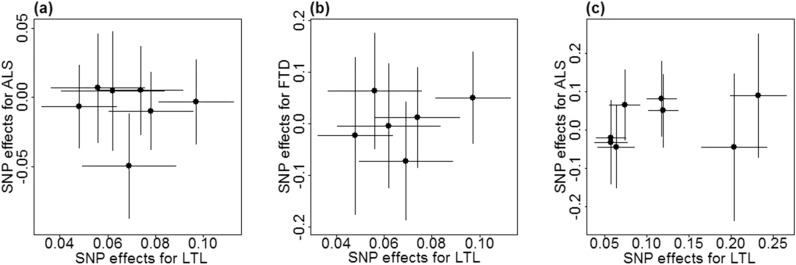
Relationship between effect sizes on LTL and ALS/FTD for SNPs served as instrumental variables. Results are shown for seven SNPs of ALS (**a**) and six SNPs of FTD (**b**) in the European population. Results are also displayed for eight SNPs of ALS in the Asian population (**c**). In each panel, horizontal/vertical lines represent the 95% confidence intervals.

We now validated the causal effect of LTL on ALS estimated above through various sensitivity analyses. Here, we mainly focused on the relationship between LTL and ALS in the European population (Table 4). The weighted median and maximum likelihood methods generate similar null causal effect estimates. In particular, the OR is estimated to be 1.06 (95% CI 0.85–1.32, *p* = 0.624) by the weighted median method and 1.10 (95% CI 0.92–1.32, *p* = 0.290) by the maximum likelihood approach. Both the LOO (Supplementary Table S10) and MR-PRESSO analyses indicate that no instrument outliers exist (see also Fig. 1). The MR-Egger regression provides little evidence of horizontal pleiotropy as its intercept is not significantly deviated from zero (0.006, 95% CI − 0.079–0.090, *p* = 0.872). The results of sensitivity analyses for LTL and ALS in the Asian population as well as for LTL and FTD in the European population are summarized in Supplementary Tables S11-S12.

Finally, we conducted GSMR with genotypes of 503 European individuals or 504 Asian individuals in the 1,000 Genomes Project as reference panel^63^. It is shown that GSMR generates consistent causal effect estimates with previous results (Table 4), again supporting the null association between LTL and ALS/FTD. In addition, the HEIDI-outlier approach does not detect any instruments that exhibit apparent pleiotropic effects, implying the observed association between LTL and ALS/FTD would be not confounded by pleiotropy.

### Mediation analysis of the role between LTL, lipids and ALS/FTD

Although we do not find statistically significant evidence that LTL causally influences ALS/FTD in the direct biological pathway, we cannot fully exclude the probability that LTL may impact ALS/FTD via other indirect pathways. We selected six or eight index association SNPs to serve as instrumental variables for LTL on lipids in the European and Asian populations, respectively. In the European population, the causal effects per SD decrease of LTL on HDL and TG are 0.08 (95% CI 0.03–0.14, *p* = 0.005) and − 0.10 (95% CI − 0.15 to − 0.04, *p* = 0.001), respectively (Table 5). However, HDL and TG are not associated with ALS, implying there may be no indirect effects of LTL on ALS mediated by HDL or TG.

**Table 5:** Three directions of the relation with exposure to mediator, mediator to outcome and exposure to outcome.

Pop	Exposure	Mediator	*a*	SE (*a*)	*p*	Mediator	Outcome	*b*	SE (*b*)	*p*	Exposure	Outcome	*c*	SE (*c*)	*p*
EUR	LTL	HDL	0.082	0.029	0.005	HDL	ALS	0.013	0.039	0.743	LTL	ALS	0.097	0.089	0.274
	**LTL**	**LDL**	**− 0.060**	**0.031**	**0.057**	**LDL**	**ALS**	**− 0.110**	**0.031**	**3.41E−04**	LTL	ALS	0.097	0.089	0.274
	**LTL**	**TC**	**− 0.059**	**0.031**	**0.052**	**TC**	**ALS**	**− 0.098**	**0.032**	**0.002**	LTL	ALS	0.097	0.089	0.274
	LTL	TG	− 0.095	0.028	0.001	TG	ALS	− 0.045	0.044	0.309	LTL	ALS	0.097	0.089	0.274
	LTL	HDL	0.082	0.029	0.005	HDL	FTD	− 0.035	0.125	0.786	LTL	FTD	− 0.208	0.308	0.498
	LTL	LDL	− 0.060	0.031	0.057	LDL	FTD	− 0.139	0.107	0.196	LTL	FTD	− 0.208	0.308	0.498
	LTL	TC	− 0.059	0.031	0.052	TC	FTD	− 0.142	0.104	0.172	LTL	FTD	− 0.208	0.308	0.498
	LTL	TG	− 0.095	0.028	0.001	TG	FTD	− 0.018	0.140	0.898	LTL	FTD	− 0.208	0.308	0.498
Asian	LTL	HDL	− 0.020	0.022	0.366	HDL	ALS	0.108	0.129	0.404	LTL	ALS	− 0.284	0.180	0.116
	LTL	LDL	0.003	0.023	0.898	LDL	ALS	− 0.234	0.131	0.073	LTL	ALS	− 0.284	0.180	0.116
	LTL	TC	− 0.002	0.014	0.911	TC	ALS	− 0.276	0.214	0.197	LTL	ALS	− 0.284	0.180	0.116
	LTL	TG	0.018	0.014	0.214	TG	ALS	0.160	0.195	0.414	LTL	ALS	− 0.284	0.180	0.116

*Pop* population, *EUR* European, *LTL* leukocyte telomere length, *HDL* high density lipoprotein, *LDL* low density lipoprotein, *TC* total cholesterol, *TG* triglycerides, *ALS* amyotrophic lateral sclerosis, *FTD* frontotemporal dementia, *p p* value,

The effect size and the standard error of the relationship with Exposure to Mediator, Mediator to Outcome and Exposure to Outcome are denoted as *a*, *b*, *c* and SE(*a*), SE(*b*), SE(*c*), respectively.

The marginally significant causal association between LTL and LDL/TC and the significant causal association between LDL/TC and ALS in the European population are shown in bold.

On the other hand, the causal effect per SD decrease of LTL on LDL and TC are − 0.06 (95% CI − 0.12–0.00, *p* = 0.057) and − 0.06 (95% CI − 0.12–0.00, *p* = 0.052), respectively, both of which are marginally significant at the level of 0.05. Moreover, in the European population these two lipids are causally associated with ALS: the ORs per SD decrease of LDL (~ 37.0 mg/dL) and TC (~ 42.6 mg/dL) on ALS are − 0.11 (95% CI − 0.17 to − 0.05, *p* = 3.41E−04) and − 0.10 (95% CI − 0.16 to − 0.04, *p* = 0.002), respectively. Therefore, based on the basic principle of the classical mediation inference, we can reasonably state that there likely exists potential indirect effect of LTL on ALS mediated by LDL (*ab* = 0.007 and *p* = 0.079) or TC (*ab* = 0.006 and *p* = 0.092) (Table 6). More specifically, in terms of the suggestive evidence of mediation effects displayed above, in the European population we can conclude that shorter LTL can reduce the LDL/TC level, which in turn results in the lower risk of ALS. However, we fail to repeat such mediation association for ALS in the Asian population or for FTD in the European population (Tables 5, 6).

**Table 6:** Mediation analysis of the role between telomere length, lipids and ALS/FTD.

Pop	Exposure	Mediator	Outcome	*ab* (*S*_*ab*_)	95% CI	*Z*	*p*
EUR	LTL	HDL	ALS	0.001 (0.003)	− 0.005–0.007	0.354	0.724
	**LTL**	**LDL**	**ALS**	**0.007 (0.004)**	**−** **0.001–0.014**	**1.754**	**0.079**
	**LTL**	**TC**	**ALS**	**0.006 (0.003)**	**−** **0.001–0.013**	**1.682**	**0.092**
	LTL	TG	ALS	0.004 (0.004)	− 0.004–0.012	1.021	0.307
	LTL	HDL	FTD	− 0.003 (0.010)	− 0.022–0.016	− 0.298	0.766
	LTL	LDL	FTD	0.008 (0.007)	− 0.005–0.022	1.194	0.232
	LTL	TC	FTD	0.008 (0.007)	− 0.005–0.022	1.227	0.220
	LTL	TG	FTD	0.002 (0.013)	− 0.023–0.027	0.134	0.893
Asian	LTL	HDL	ALS	− 0.002 (0.002)	− 0.006–0.002	− 1.048	0.295
	LTL	LDL	ALS	− 0.001 (0.004)	− 0.009–0.008	− 0.157	0.875
	LTL	TC	ALS	0.001 (0.002)	− 0.004–0.005	0.223	0.824
	LTL	TG	ALS	0.003 (0.003)	− 0.003–0.009	0.916	0.360

*Pop* population, *EUR* European, *LTL* leukocyte telomere length, *HDL* high density lipoprotein, *LDL* low density lipoprotein, *TC* total cholesterol, *TG* triglycerides, *ALS* amyotrophic lateral sclerosis, *FTD* frontotemporal dementia, *ab* the mediation effect, *S*_*ab*_ standard error of the mediation effect, CI, *Z* and *p* represent confidence internal, *Z* statistic and *p* value, respectively.

The marginally significant mediated effect of LTL on the risk of ALS by LDL or TC are shown in bold.

**Figure 2:**
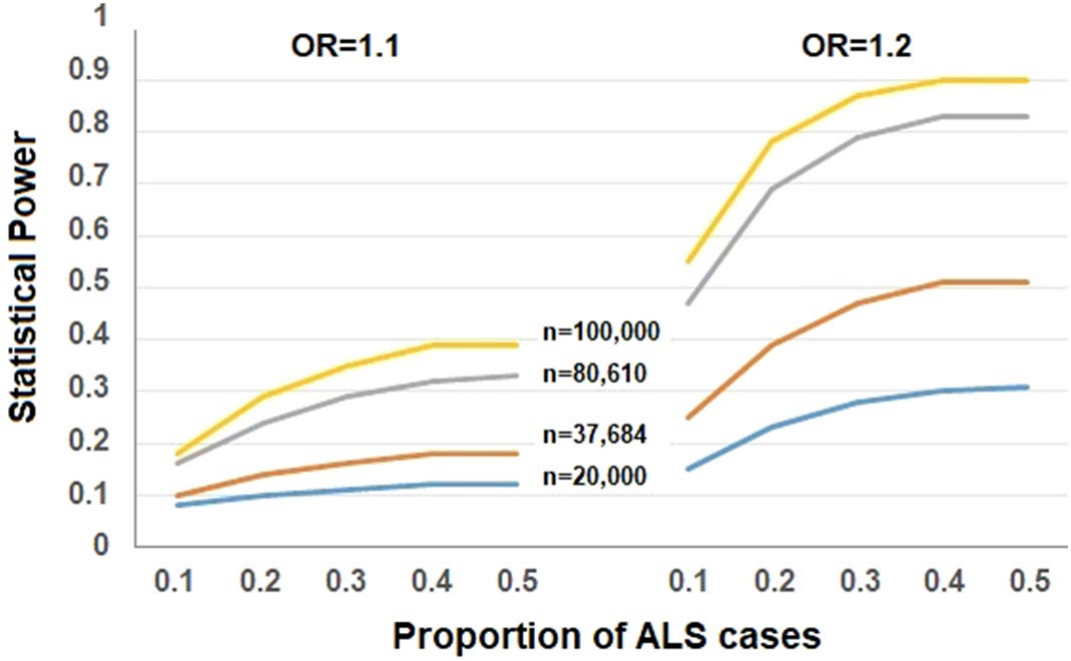
Statistical power calculation for the causal effect of LTL on ALS estimated with the method proposed in^64^. In the calculation, the total phenotypic variance explained by instrumental variables was 1.26% and the proportion of ALS cases varied from 0.1 to 0.5, the significance level was 0.05, the sample size was 20,000, 37,684, 80,610 or 100,000, and the OR = 1.10 or 1.20.

Finally, we examined whether the lack of detectable non-zero causal effect of LTL on ALS is due to the lack of statistical power. We calculated the statistical power to detect an OR of 1.10 or 1.20 (approximately equal the estimated causal effects above) per SD decrease of LTL on the risk of ALS following an analytic approach (https://cnsgenomics.shinyapps.io/mRnd/)^64^. It is shown the estimated statistical power is only 15% or 44% (Fig. 2), indicating we have low to moderate power to identify such causal effect with current sample sizes if LTL is indeed causally associated with the risk of ALS.

## Discussion

In the present study we have implemented a comprehensive two-sample MR analysis to dissect whether there exists causal relationship between LTL and the risk of ALS. To our knowledge, this is the first MR study to investigate the relationship between LTL and ALS using statistical genetic approaches via summary statistics available from large-scale GWAS. We found that an indirect effect of LTL on ALS might be mediated by LDL or TC, although our MR analysis did not support the existence of direct causal association between LTL and ALS/FTD. These findings were robust to the choice of statistical methods and were carefully validated through various sensitivity analyses.

Our results are not fully consistent with those in previous studies (Table 1). For example, previous studies displayed distinct association in direction and magnitude between LTL and ALS in the European population^9,14^. Compared to those prior work, our study has the advantage of larger sample size (20,806/59,804 vs. 6,100/7,125 and 1,241/335) and thus holds higher power. In addition, we recognize that the estimated causal effect of shorter LTL on ALS had an opposite direction in the two populations although they were non-significant in neither population. Given the substantial difference of ALS in clinical features and molecular mechanisms between European and Asian populations^65–69^, this finding may not be unexpected. As little has been known about the causal factors for ALS to date^1^, our study therefore contributes considerably to the research area on the relationship between LTL and the risk of ALS, and has potential implication for the therapeutic intervention of ALS.

Besides revealing the null causal relationship between LTL and ALS in the two populations, our study also, at least in part, offers empirical evidence for several questions that were previously unanswered. First, we also validated that the causal association did not hold between LTL and FTD, which might be partly due to the fact that FTD and ALS share extensive similarities in clinical manifestation and genetic foundation^33–35^. Second, unlike previous studies, the mediation analysis was performed, which provided suggestive evidence supporting the mediation role of LDL or TC in the causal pathway from LTL to ALS in the European population. Therefore, interventions by targeting LDL or TC can be considered as a potential promising manner to counteract the effect of LTL changes on the risk of ALS.

Of course, our study is not without drawbacks. In addition to the general MR limitations similar to other work (e.g. the linear effect assumption), other potential shortcomings should be mentioned^17,18,70^. First, in our study telomere length measured in blood leukocytes was employed; however, LTL may be not representative of telomere length in tissues that are most relevant to ALS. Second, we note that the Asian-ALS GWAS and the European-FTD GWAS did not adjust the effect of age in their association analyses (Supplementary Text), which may bias our estimates because telomere length would become short with age. However, we cannot examine the causal effect between LTL and ALS/FTD stratified by the age group^1,6^ as it is impossible for us to obtain individual-level GWAS datasets due to privacy concerns. Third, as *C9orf72*, *TARDBP* and *FUS* are known to be the most common mutated genes in ALS^71–73^. Removing ALS patients with mutations in those genes and performing additional sensitivity analysis can shed new lights on the relationship between LTL and ALS in more general population of sporadic ALS cases (note that excluding those special ALS cases might lead to the reduction of statistical power because of decreased sample size). Again, we cannot conduct such analysis as individual datasets are not accessible. Fourth, as shown above, our MR analysis has only limited statistical power; in addition, our mediation analysis showed that the mediated effect of LTL on the risk of ALS by LDL or TC was only marginally significant. Therefore, studies with larger sample size are required to validate our results in both the European and Asian populations.

## Conclusions

Our MR study did not support the causal association between LTL and the risk of ALS in neither the European population nor the Asian population, but provided suggestive evidence supporting the mediation role of LDL or TC on the influence of LTL and ALS in the European population.

## Supplementary information

Supplementary Information 1.

Supplementary Information 2.

Supplementary Information 3.

## References

[1] Al-Chalabi A, Hardiman O (2013). The epidemiology of ALS: a conspiracy of genes, environment and time. Nat. Rev. Neurol..

[2] Armon C (2019). Smoking is a cause of amyotrophic lateral sclerosis. High low-density lipoprotein cholesterol levels? Unsure. Ann. Neurol..

[3] Bandres-Ciga S (2019). Shared polygenic risk and causal inferences in amyotrophic lateral sclerosis. Ann. Neurol..

[4] Zhan Y, Fang F (2019). Smoking and amyotrophic lateral sclerosis: a mendelian randomization study. Ann. Neurol..

[5] Arthur KC (2016). Projected increase in amyotrophic lateral sclerosis from 2015 to 2040. Nat. Commun..

[6] Kong CM, Lee XW, Wang X (2013). Telomere shortening in human diseases. FEBS J..

[7] Shay JW, Wright WE (2019). Telomeres and telomerase: three decades of progress. Nat. Rev. Genet..

[8] Zhu H, Belcher M, van der Harst P (2011). Healthy aging and disease: role for telomere biology?. Clin. Sci. (Lond.).

[9] Haycock PC (2017). Association between telomere length and risk of cancer and non-neoplastic diseases a Mendelian randomization study. Jama Oncol..

[10] Zhang C (2015). Genetic determinants of telomere length and risk of common cancers: a Mendelian randomization study. Hum. Mol. Genet..

[11] Zhan Y (2017). Exploring the causal pathway from telomere length to coronary heart disease: a network Mendelian randomization study. Circ. Res..

[12] Martin-Ruiz C (2020). Senescence and inflammatory markers for predicting clinical progression in Parkinson's disease: the ICICLE-PD study. J. Parkinsons Dis..

[13] De Felice B (2014). Telomerase expression in amyotrophic lateral sclerosis (ALS) patients. J. Hum. Genet..

[14] Al Khleifat A (2019). Telomere length is greater in ALS than in controls: a whole genome sequencing study. Amyotroph. Lateral. Scler. Frontotemporal. Degener,.

[15] Linkus B (2016). Telomere shortening leads to earlier age of onset in ALS mice. Aging (Albany N. Y.).

[16] Fall T (2013). The role of adiposity in cardiometabolic traits: a Mendelian randomization analysis. PLoS Med..

[17] Sleiman PM, Grant SF (2010). Mendelian randomization in the era of genomewide association studies. Clin. Chem..

[18] Paternoster L, Tilling K, Davey Smith G (2017). Genetic epidemiology and Mendelian randomization for informing disease therapeutics: conceptual and methodological challenges. PLoS Genet..

[19] Haycock PC (2016). Best (but oft-forgotten) practices: the design, analysis, and interpretation of Mendelian randomization studies. Am. J. Clin. Nutr..

[20] Nicolas A (2018). Genome-wide analyses identify KIF5A as a novel ALS gene. Neuron.

[21] Codd V (2013). Identification of seven loci affecting mean telomere length and their association with disease. Nat. Genet..

[22] Dorajoo R (2019). Loci for human leukocyte telomere length in the Singaporean Chinese population and trans-ethnic genetic studies. Nat. Commun..

[23] Visscher PM (2017). 10 Years of GWAS discovery: biology, function, and translation. Am. J. Hum. Genet..

[24] Welter D (2014). The NHGRI GWAS Catalog, a curated resource of SNP-trait associations. Nucleic Acids Res..

[25] Zeng P, Wang T, Zheng J, Zhou X (2019). Causal association of type 2 diabetes with amyotrophic lateral sclerosis: new evidence from Mendelian randomization using GWAS summary statistics. BMC Med..

[26] Yu, X. *et al.* Relationship between birth weight and chronic kidney disease: evidence from systematics review and two-sample Mendelian randomization analysis. *Hum. Mol. Genet.* ddaa074 (2020). 10.1093/hmg/ddaa074 32329512

[27] Yu x (2020). Alcohol drinking and amyotrophic lateral sclerosis: an instrumental variable causal inference. Ann. Neurol..

[28] Zeng P, Zhou X (2019). Causal association between birth weight and adult diseases: evidence from a Mendelian randomization analysis. Front. Genet..

[29] Zhao JV, Schooling CM (2019). Effect of linoleic acid on ischemic heart disease and its risk factors: a Mendelian randomization study. BMC Med..

[30] Tyrrell J (2016). Height, body mass index, and socioeconomic status: mendelian randomisation study in UK Biobank. Br. Med. J..

[31] Larsson SC, Burgess S, Michaëlsson K (2017). Association of genetic variants related to serum calcium levels with coronary artery disease and myocardial infarction. JAMA.

[32] Ahmad OS (2015). A Mendelian randomization study of the effect of type-2 diabetes on coronary heart disease. Nat. Commun..

[33] Diekstra FP (2014). C9orf72 and UNC13A are shared risk loci for amyotrophic lateral sclerosis and frontotemporal dementia: a genome-wide meta-analysis. Ann. Neurol..

[34] Lattante S, Ciura S, Rouleau GA, Kabashi E (2015). Defining the genetic connection linking amyotrophic lateral sclerosis (ALS) with frontotemporal dementia (FTD). Trends Genet..

[35] Karch CM (2018). Selective genetic overlap between amyotrophic lateral sclerosis and diseases of the frontotemporal dementia spectrum. JAMA Neurol..

[36] Ferrari R (2014). Frontotemporal dementia and its subtypes: a genome-wide association study. Lancet Neurol..

[37] Benyamin B (2017). Cross-ethnic meta-analysis identifies association of the GPX3-TNIP1 locus with amyotrophic lateral sclerosis. Nat. Commun..

[38] Bowden J (2016). Assessing the suitability of summary data for two-sample Mendelian randomization analyses using MR-Egger regression: the role of the I-2 statistic. Int. J. Epidemiol..

[39] Burgess S, Small DS, Thompson SG (2017). A review of instrumental variable estimators for Mendelian randomization. Stat. Methods Med. Res..

[40] Hartwig FP, Davey Smith G, Bowden J (2017). Robust inference in summary data Mendelian randomization via the zero modal pleiotropy assumption. Int. J. Epidemiol..

[41] Yavorska OO, Burgess S (2017). Mendelian randomization: an R package for performing Mendelian randomization analyses using summarized data. Int. J. Epidemiol..

[42] Bowden J, Smith GD, Haycock PC, Burgess S (2016). Consistent estimation in Mendelian randomization with some invalid instruments using a weighted median estimator. Genet. Epidemiol..

[43] Burgess S, Butterworth A, Thompson SG (2013). Mendelian randomization analysis with multiple genetic variants using summarized data. Genet. Epidemiol..

[44] Noyce AJ (2017). Estimating the causal influence of body mass index on risk of Parkinson disease: a Mendelian randomisation study. PLoS Med..

[45] Verbanck M, Chen C-Y, Neale B, Do R (2018). Detection of widespread horizontal pleiotropy in causal relationships inferred from Mendelian randomization between complex traits and diseases. Nat. Genet..

[46] Burgess S, Thompson SG (2017). Interpreting findings from Mendelian randomization using the MR-Egger method. Eur. J. Epidemiol..

[47] Zhu Z (2018). Causal associations between risk factors and common diseases inferred from GWAS summary data. Nat. Commun..

[48] Laimer M (2016). Telomere length increase after weight loss induced by bariatric surgery: results from a 10 year prospective study. Int. J. Obes. (Lond.).

[49] Rehkopf DH (2016). Leukocyte telomere length in relation to 17 biomarkers of cardiovascular disease risk: a cross-sectional study of US adults. PLoS Med..

[50] Revesz D, Milaneschi Y, Verhoeven JE, Penninx BWJH (2014). Telomere length as a marker of cellular aging is associated with prevalence and progression of metabolic syndrome. J. Clin. Endocrinol. Metab..

[51] Al-Attas OS (2010). Adiposity and insulin resistance correlate with telomere length in middle-aged Arabs: the influence of circulating adiponectin. Eur. J. Endocrinol..

[52] Weng Q (2019). Leukocyte telomere length, lipid parameters and gestational diabetes risk: a case-control study in a Chinese population. Sci. Rep..

[53] Zeng P, Zhou X (2019). Causal effects of blood lipids on amyotrophic lateral sclerosis: a Mendelian randomization study. Hum. Mol. Genet..

[54] Dupuis L (2008). Dyslipidemia is a protective factor in amyotrophic lateral sclerosis. Neurology.

[55] MacKinnon DP, Fairchild AJ, Fritz MS (2007). Mediation analysis. Annu. Rev. Psychol..

[56] MacKinnon DP (2008). Introduction to statistical mediation analysis.

[57] MacKinnon DP, Fairchild AJ (2009). Current directions in mediation analysis. Curr. Dir. Psychol. Sci..

[58] Richiardi L, Bellocco R, Zugna D (2013). Mediation analysis in epidemiology: methods, interpretation and bias. Int. J. Epidemiol..

[59] VanderWeele TJ (2016). Mediation analysis: a Practitioner's guide. Annu. Rev. Public Health.

[60] Burgess S, Daniel RM, Butterworth AS, Thompson SG, Consortium EP-I (2015). Network Mendelian randomization: using genetic variants as instrumental variables to investigate mediation in causal pathways. Int. J. Epidemiol..

[61] Willer CJ (2013). Discovery and refinement of loci associated with lipid levels. Nat. Genet..

[62] Cragg JG, Donald SG (1993). Testing identifiability and specification in instrumental variable models. Economet. Theor..

[63] The 1000 Genomes Project Consortium (2015). A global reference for human genetic variation. Nature.

[64] Brion M-JA, Shakhbazov K, Visscher PM (2013). Calculating statistical power in Mendelian randomization studies. Int. J. Epidemiol..

[65] Chio A (2013). Global epidemiology of amyotrophic lateral sclerosis: a systematic review of the published literature. Neuroepidemiology.

[66] Liu MS, Cui LY, Fan DS, Assoc CA (2014). Age at onset of amyotrophic lateral sclerosis in China. Acta Neurol. Scand..

[67] Ogaki K (2012). Analysis of C9orf72 repeat expansion in 563 Japanese patients with amyotrophic lateral sclerosis. Neurobiol. Aging.

[68] Diez Roux AV (2009). Race/ethnicity and telomere length in the Multi-Ethnic Study of Atherosclerosis. Aging Cell.

[69] Davidson EM (2014). Consideration of ethnicity in guidelines and systematic reviews promoting lifestyle interventions: a thematic analysis. Eur. J. Public Health.

[70] Sheehan NA, Didelez V, Burton PR, Tobin MD (2008). Mendelian randomisation and causal inference in observational epidemiology. PLoS Med..

[71] Zou ZY (2017). Genetic epidemiology of amyotrophic lateral sclerosis: a systematic review and meta-analysis. J. Neurol. Neurosurg. Psychiatry.

[72] Renton AE, Chio A, Traynor BJ (2014). State of play in amyotrophic lateral sclerosis genetics. Nat. Neurosci..

[73] Onesto E (2016). Gene-specific mitochondria dysfunctions in human TARDBP and C9ORF72 fibroblasts. Acta Neuropathol. Commun..

[74] Forero DA (2016). Telomere length in Parkinson's disease: a meta-analysis. Exp. Gerontol..

[75] Wang H (2008). Telomere length and risk of Parkinson's disease. Mov. Disord..

[76] Eerola J (2010). No evidence for shorter leukocyte telomere length in Parkinson's disease patients. J. Gerontol. A Biol. Sci. Med. Sci..

[77] Schurks M (2014). Telomere length and Parkinson's disease in men: a nested case-control study. Eur. J. Neurol..

[78] Guan JZ (2008). A percentage analysis of the telomere length in Parkinson's disease patients. J. Gerontol. A Biol. Sci. Med. Sci..

[79] Gao K (2019). Exploring the causal pathway from telomere length to Alzheimer's disease: an update Mendelian randomization study. Front. Psychiatry.

[80] Guo YF, Yu HN (2019). Leukocyte telomere length shortening and Alzheimer's disease etiology. J. Alzheimers Dis..

[81] Zhan Y (2015). Telomere length shortening and Alzheimer disease—a Mendelian randomization study. JAMA Neurol..

[82] Zhan Y, Hagg S (2018). Telomere length shortening in Alzheimer's disease: procedures for a causal investigation using single nucleotide polymorphisms in a Mendelian randomization study. Methods Mol. Biol..

[83] Forero DA (2016). Meta-analysis of telomere length in Alzheimer's disease. J. Gerontol. A Biol. Sci. Med. Sci..

[84] Honig LS, Kang MS, Schupf N, Lee JH, Mayeux R (2012). Association of shorter leukocyte telomere repeat length with dementia and mortality. Arch. Neurol..

[85] Martin-Ruiz C (2006). Telomere length predicts poststroke mortality, dementia, and cognitive decline. Ann. Neurol..

[86] Shim H (2015). A multivariate genome-wide association analysis of 10 LDL subfractions, and their response to statin treatment, in 1868 Caucasians. PLoS ONE.

[87] Burgess S, Thompson SG, Collaboration CCG (2011). Avoiding bias from weak instruments in Mendelian randomization studies. Int. J. Epidemiol..

[88] Burgess S, Thompson SG (2012). Improving bias and coverage in instrumental variable analysis with weak instruments for continuous and binary outcomes. Stat. Med..

[89] Kanai M (2018). Genetic analysis of quantitative traits in the Japanese population links cell types to complex human diseases. Nat. Genet..

