# Supplementary material for: Mendelian randomization implies no direct causal association between leukocyte telomere length and amyotrophic lateral sclerosis
Source: Sci Rep. 2020 Jul 22;10:12184. doi: 10.1038/s41598-020-68848-9 (PMC7376149; doi:10.1038/s41598-020-68848-9)
Supplement: Supplementary file 2 — Supplementary Information 2. [file 41598_2020_68848_MOESM2_ESM.docx]

**Mendelian randomization implies no direct causal association between leukocyte telomere length and amyotrophic lateral sclerosis**

**Yixin Gao^1$^, Ting Wang^1$^, Xinghao Yu^1^, International FTD-Genomics Consortium (IFGC), Huashuo Zhao^1, 2*^, Ping Zeng^1, 2*^**

^1^ Department of Epidemiology and Biostatistics, School of Public Health, Xuzhou Medical University, Xuzhou, Jiangsu, 221004, PR China

^2^ Center for Medical Statistics and Data Analysis, School of Public Health, Xuzhou Medical University, Xuzhou, Jiangsu, 221004, PR China

^$^ Co-first authors

^*^ Corresponding authors: hszhao@xzhmu.edu.cn and [zpstat@xzhmu.edu.cn](mailto:zpstat@xzhmu.edu.cn)

**Supplementary Results**

| SNP | GENE | CHR | BP | A1/A2 | BETA | SE | *p* |
| --- | --- | --- | --- | --- | --- | --- | --- |
| rs10463311 | *TNIP1* | 5 | 150,410,835 | T/C | -0.085 | 0.016 | 4.00E-08 |
| rs3849943 | *C9orf72* | 9 | 27,543,382 | T/C | -0.176 | 0.016 | 3.77E-30 |
| rs117027576 | *SDR9C7* | 12 | 57,316,603 | T/G | -0.316 | 0.057 | 2.28E-08 |
| rs118082508 | *SDR9C7* | 12 | 57,318,819 | T/C | 0.318 | 0.057 | 1.97E-08 |
| rs113247976 | *KIF5A* | 12 | 57,975,700 | T/C | 0.322 | 0.052 | 6.43E-10 |
| rs116900480 | *RPL21P103* | 12 | 58,656,105 | T/C | 0.317 | 0.051 | 6.60E-10 |
| rs142321490 | *RPL21P103* | 12 | 58,676,132 | C/G | 0.317 | 0.051 | 6.15E-10 |
| rs74654358 | *TBK1* | 12 | 64,881,967 | A/G | 0.198 | 0.034 | 4.66E-09 |
| rs12973192 | *UNC13A* | 19 | 17,753,239 | C/G | -0.121 | 0.015 | 3.92E-15 |
| rs75087725 | *AP001062.7* | 21 | 45,753,117 | A/C | 0.515 | 0.067 | 1.85E-14 |

Supplementary Table S1. Summary information for index SNPs of ALS in the European population. Note: SNP: the label of single-nucleotide polymorphism; CHR: chromosome; BP: base position; A1: effect allele; A2: alternative allele; BETA: SNP effect size; SE: standard error of the SNP effect size; *p*: *p* value.

| SNP | GENE | CHR | BP | A1/A2 | BETA | SE | *p* |
| --- | --- | --- | --- | --- | --- | --- | --- |
| rs9268877 | *HLA-DRB9* | 6 | 32,431,147 | A/G | 0.286 | 0.045 | 1.65E-10 |
| rs9268852 | *HLA-DRB9* | 6 | 32,429,594 | A/G | 0.280 | 0.044 | 1.68E-10 |
| rs9268863 | *HLA-DRB9* | 6 | 32,430,289 | A/G | 0.279 | 0.044 | 2.20E-10 |
| rs9268881 | *HLA-DRB9* | 6 | 32,431,606 | A/T | -0.288 | 0.045 | 2.39E-10 |
| rs9268888 | *HLA-DRB9* | 6 | 32,431,867 | T/G | -0.302 | 0.048 | 2.99E-10 |
| rs9268912 | *HLA-DRB9* | 6 | 32,432,509 | A/C | -0.300 | 0.048 | 3.88E-10 |
| rs9268883 | *HLA-DRB9* | 6 | 32,431,638 | A/T | -0.289 | 0.046 | 4.49E-10 |
| rs9268893 | *HLA-DRB9* | 6 | 32,431,927 | T/C | -0.290 | 0.048 | 1.42E-09 |
| rs9268856 | *HLA-DRB9* | 6 | 32,429,719 | A/C | -0.285 | 0.050 | 1.30E-08 |
| rs9268854 | *HLA-DRB9* | 6 | 32,429,672 | A/G | 0.288 | 0.051 | 1.47E-08 |
| rs9268855 | *HLA-DRB9* | 6 | 32,429,675 | A/G | -0.288 | 0.051 | 1.47E-08 |
| rs9268862 | *HLA-DRB9* | 6 | 32,430,167 | A/C | 0.284 | 0.050 | 1.48E-08 |
| rs9268845 | *HLA-DRB9* | 6 | 32,429,204 | T/C | -0.295 | 0.052 | 1.48E-08 |
| rs9268857 | *HLA-DRB9* | 6 | 32,429,739 | A/G | 0.283 | 0.050 | 1.62E-08 |
| rs9268850 | *HLA-DRB9* | 6 | 32,429,477 | A/G | -0.286 | 0.051 | 1.69E-08 |
| rs9268840 | *HLA-DRB9* | 6 | 32,428,804 | T/C | -0.284 | 0.051 | 2.06E-08 |
| rs7747521 | *HLA-DRB9* | 6 | 32,431,105 | A/G | 0.303 | 0.054 | 2.26E-08 |
| rs4434496 | *HLA-DRB9* | 6 | 32,430,508 | T/C | -0.289 | 0.052 | 2.31E-08 |
| rs4428528 | *HLA-DRB9* | 6 | 32,430,362 | C/G | 0.289 | 0.052 | 2.36E-08 |
| rs7766843 | *HLA-DRB9* | 6 | 32,430,729 | T/C | -0.293 | 0.053 | 2.37E-08 |
| rs6940440 | *HLA-DRB9* | 6 | 32,429,087 | A/C | 0.274 | 0.049 | 2.41E-08 |
| rs7766854 | *HLA-DRB9* | 6 | 32,430,752 | T/C | -0.293 | 0.053 | 2.47E-08 |
| rs7747010 | *HLA-DRB9* | 6 | 32,430,800 | A/G | 0.299 | 0.054 | 3.11E-08 |
| rs7747025 | *HLA-DRB9* | 6 | 32,430,814 | A/G | 0.299 | 0.054 | 3.11E-08 |
| rs4280993 | *HLA-DRB9* | 6 | 32,430,604 | A/C | -0.289 | 0.052 | 3.20E-08 |
| rs7746751 | *HLA-DRB9* | 6 | 32,430,867 | A/G | -0.289 | 0.052 | 3.21E-08 |
| rs7746922 | *HLA-DRB9* | 6 | 32,430,975 | A/C | -0.292 | 0.053 | 3.82E-08 |
| rs9268885 | *HLA-DRB9* | 6 | 32,431,705 | T/C | -0.305 | 0.056 | 4.43E-08 |
| rs1980493 | *BTNL2* | 6 | 32,363,215 | T/C | 0.329 | 0.060 | 4.94E-08 |

Supplementary Table S2. Summary information for index SNPs of FTD in the European population. Note: SNP: the label of single-nucleotide polymorphism; CHR: chromosome; BP: base position; A1: effect allele; A2: alternative allele; BETA: SNP effect size; SE: standard error of the SNP effect size; *p*: *p* value.

| SNP | GENE | CHR | BP | A1/A2 | LTL | | | |  | FTD | | | | PVE | *F* |
| --- | --- | --- | --- | --- | --- | --- | --- | --- | --- | --- | --- | --- | --- | --- | --- |
|  |  |  |  |  | BETA | SE | *p* | *N* |  | BETA | SE | *p* | *N* |  |  |
| rs11125529 | *TERT* | 2 | 54,329,370 | C/A | -0.056 | 0.010 | 4.48E-08 | 37,653 |  | -0.064 | 0.057 | 0.262 | 12,928 | 8.32E-04 | 31.4 |
| rs10936599 | *TERC* | 3 | 170,974,795 | T/C | -0.097 | 0.008 | 2.54E-31 | 37,669 |  | -0.051 | 0.045 | 0.261 | 12,928 | 3.89E-03 | 147.0 |
| rs7675998 | *ZNF208* | 4 | 164,227,270 | A/G | -0.074 | 0.009 | 4.35E-16 | 34,694 |  | -0.013 | 0.050 | 0.792 | 12,928 | 1.94E-03 | 67.6 |
| rs9420907 | *ACYP2* | 10 | 105,666,455 | A/C | -0.069 | 0.010 | 6.90E-11 | 37,653 |  | 0.072 | 0.058 | 0.217 | 12,928 | 1.26E-03 | 47.6 |
| rs8105767 | *RTEL1* | 19 | 22,007,281 | A/G | -0.048 | 0.008 | 1.11E-09 | 37,499 |  | 0.023 | 0.078 | 0.767 | 12,928 | 9.59E-04 | 36.0 |
| rs755017 | *OBFC1* | 20 | 61,892,066 | A/G | -0.062 | 0.011 | 6.71E-09 | 37,113 |  | 0.003 | 0.062 | 0.956 | 12,928 | 8.55E-04 | 31.8 |

Supplementary Table S3. Summary information of instrumental variables for LTL and FTD in the European population. Note: SNP: the label of single-nucleotide polymorphism; CHR: chromosome; BP: base position; A1: effect allele, indicates the allele that is associated with shorter LTL, explaining why all the BETA estimates are negative; A2: alternative allele; BETA: SNP effect size; SE: standard error of the SNP effect size; PVE: proportion of variance explained by the SNP; *p*, *N*, and *F* represent *p* value, sample size and *F* statistic, respectively. The information of rs2736100 is missed in the FTD GWAS data set. All the selected instruments collectively explain about 0.97% phenotypic variation of LTL at the observed scale. For these instrumental variables, all the *F* statistics are above 10 (ranging from 31.4 to 147.0) with an average *F* statistic of 60.2 and an overall *F* statistic of 60.7, suggesting that the selected genetic variants have sufficient strong effects for instrument and the bias of weak instrument is unlikely.

| SNP | GENE | CHR | BP | A1/A2 | LTL | | | |  | ALS | | | | PVE | *F* |
| --- | --- | --- | --- | --- | --- | --- | --- | --- | --- | --- | --- | --- | --- | --- | --- |
|  |  |  |  |  | BETA | SE | *p* | *N* |  | BETA | SE | *p* | *N* |  |  |
| rs3219104 | *PARP1* | 1 | 226,562,621 | A/C | -0.074 | 0.009 | 2.23E-16 | 23,096 |  | -0.066 | 0.047 | 0.161 | 4,084 | 2.92E-03 | 67.6 |
| rs2293607 | *TERC* | 3 | 169,482,335 | C/T | -0.120 | 0.009 | 7.57E-39 | 23,096 |  | -0.050 | 0.048 | 0.306 | 4,084 | 7.64E-03 | 177.8 |
| rs10857352 | *NAF1* | 4 | 164,101,482 | A/G | -0.064 | 0.011 | 4.85E-09 | 23,096 |  | 0.044 | 0.054 | 0.419 | 4,084 | 1.46E-03 | 33.8 |
| rs7705526 | *TERT* | 5 | 1,285,974 | C/A | -0.118 | 0.009 | 2.61E-38 | 23,096 |  | -0.081 | 0.050 | 0.104 | 4,084 | 7.39E-03 | 171.9 |
| rs7776744 | *POT1* | 7 | 124,599,749 | G/A | -0.058 | 0.009 | 2.51E-10 | 23,096 |  | 0.020 | 0.049 | 0.690 | 4,084 | 1.79E-03 | 41.5 |
| rs12415148 | *OBFC1* | 10 | 105,680,586 | T/C | -0.204 | 0.020 | 2.78E-25 | 23,096 |  | 0.045 | 0.097 | 0.643 | 4,084 | 4.48E-03 | 104.0 |
| rs41293836 | *TINF2* | 14 | 24,721,327 | C/T | -0.233 | 0.017 | 2.47E-42 | 23,096 |  | -0.090 | 0.082 | 0.273 | 4,084 | 8.07E-03 | 187.8 |
| rs41309367 | *RTEL1* | 20 | 62,309,554 | T/C | -0.058 | 0.010 | 1.16E-08 | 23,096 |  | 0.033 | 0.055 | 0.537 | 4,084 | 1.45E-03 | 33.6 |

Supplementary Table S4. Summary information of instrumental variables for LTL and ALS in the Asian population. Note: SNP: the label of single-nucleotide polymorphism; CHR: chromosome; BP: base position; A1: effect allele, indicates the allele that is associated with shorter LTL, explaining why all the BETA estimates are negative; A2: alternative allele; BETA: SNP effect size; SE: standard error of the SNP effect size; PVE: proportion of variance explained by the SNP; *p*, *N*, and *F* represent *p* value, sample size and *F* statistic, respectively. All the selected instruments collectively explain about 3.52% phenotypic variation of LTL at the observed scale. For these instrumental variables, all the *F* statistics are above 10 (ranging from 33.6 to 187.8) with an average *F* statistic of 102.3 and an overall *F* statistic of 105.3, suggesting that the selected genetic variants have sufficient strong effects for instrument and the bias of weak instrument is unlikely.

| SNP | GENE | CHR | BP | A1/A2 | LTL | | | |  | lipids | | | | | PVE | *F* |
| --- | --- | --- | --- | --- | --- | --- | --- | --- | --- | --- | --- | --- | --- | --- | --- | --- |
|  |  |  |  |  | BETA | SE | *p* | *N* |  | Subtype | BETA | SE | *p* | *N* |  |  |
| rs11125529 | *TERT* | 2 | 54,329,370 | C/A | -0.056 | 0.010 | 4.48E-08 | 37,653 |  | HDL | 0.004 | 0.007 | 0.411 | 94,281 | 8.32E-04 | 31.4 |
| rs10936599 | *TERC* | 3 | 170,974,795 | T/C | -0.097 | 0.008 | 2.54E-31 | 37,669 |  | HDL | 0.003 | 0.005 | 0.761 | 94,311 | 3.89E-03 | 147.0 |
| rs7675998 | *ZNF208* | 4 | 164,227,270 | A/G | -0.074 | 0.009 | 4.35E-16 | 34,694 |  | HDL | -0.004 | 0.006 | 0.506 | 94,311 | 1.94E-03 | 67.6 |
| rs2736100 | *NAF1* | 5 | 1,339,516 | A/C | -0.078 | 0.009 | 4.38E-19 | 25,842 |  | HDL | 0.009 | 0.004 | 0.025 | 173,212 | 2.90E-03 | 75.1 |
| rs9420907 | *ACYP2* | 10 | 105,666,455 | A/C | -0.069 | 0.010 | 6.90E-11 | 37,653 |  | HDL | 0.013 | 0.007 | 0.024 | 94,299 | 1.26E-03 | 47.6 |
| rs8105767 | *RTEL1* | 19 | 22,007,281 | A/G | -0.048 | 0.008 | 1.11E-09 | 37,499 |  | HDL | 0.012 | 0.005 | 0.112 | 92,819 | 9.59E-04 | 36.0 |
| rs11125529 | *TERT* | 2 | 54,329,370 | C/A | -0.056 | 0.010 | 4.48E-08 | 37,653 |  | LDL | -0.008 | 0.007 | 0.415 | 89,859 | 8.32E-04 | 31.4 |
| rs10936599 | *TERC* | 3 | 170,974,795 | T/C | -0.097 | 0.008 | 2.54E-31 | 37,669 |  | LDL | -0.012 | 0.006 | 0.204 | 89,888 | 3.89E-03 | 147.0 |
| rs7675998 | *ZNF208* | 4 | 164,227,270 | A/G | -0.074 | 0.009 | 4.35E-16 | 34,694 |  | LDL | -0.001 | 0.006 | 0.899 | 89,888 | 1.94E-03 | 67.6 |
| rs2736100 | *NAF1* | 5 | 1,339,516 | A/C | -0.078 | 0.009 | 4.38E-19 | 25,842 |  | LDL | -0.004 | 0.004 | 0.290 | 159,322 | 2.90E-03 | 75.1 |
| rs9420907 | *ACYP2* | 10 | 105,666,455 | A/C | -0.069 | 0.010 | 6.90E-11 | 37,653 |  | LDL | 0.003 | 0.008 | 0.821 | 89,876 | 1.26E-03 | 47.6 |
| rs8105767 | *RTEL1* | 19 | 22,007,281 | A/G | -0.048 | 0.008 | 1.11E-09 | 37,499 |  | LDL | 0.001 | 0.006 | 0.988 | 88,432 | 9.59E-04 | 36.0 |
| rs11125529 | *TERT* | 2 | 54,329,370 | C/A | -0.056 | 0.010 | 4.48E-08 | 37,653 |  | TC | -0.003 | 0.007 | 0.866 | 94,563 | 8.32E-04 | 31.4 |
| rs10936599 | *TERC* | 3 | 170,974,795 | T/C | -0.097 | 0.008 | 2.54E-31 | 37,669 |  | TC | -0.018 | 0.006 | 0.019 | 94,595 | 3.89E-03 | 147.0 |
| rs7675998 | *ZNF208* | 4 | 164,227,270 | A/G | -0.074 | 0.009 | 4.35E-16 | 34,694 |  | TC | -0.005 | 0.006 | 0.469 | 94,595 | 1.94E-03 | 67.6 |
| rs2736100 | *NAF1* | 5 | 1,339,516 | A/C | -0.078 | 0.009 | 4.38E-19 | 25,842 |  | TC | -0.003 | 0.004 | 0.480 | 173,242 | 2.90E-03 | 75.1 |
| rs9420907 | *ACYP2* | 10 | 105,666,455 | A/C | -0.069 | 0.010 | 6.90E-11 | 37,653 |  | TC | 0.008 | 0.007 | 0.291 | 94,583 | 1.26E-03 | 47.6 |
| rs8105767 | *RTEL1* | 19 | 22,007,281 | A/G | -0.048 | 0.008 | 1.11E-09 | 37,499 |  | TC | 0.001 | 0.006 | 0.958 | 93,066 | 9.59E-04 | 36.0 |
| rs11125529 | *TERT* | 2 | 54,329,370 | C/A | -0.056 | 0.010 | 4.48E-08 | 37,653 |  | TG | -0.003 | 0.007 | 0.500 | 90,981 | 8.32E-04 | 31.4 |
| rs10936599 | *TERC* | 3 | 170,974,795 | T/C | -0.097 | 0.008 | 2.54E-31 | 37,669 |  | TG | -0.011 | 0.005 | 0.100 | 91,013 | 3.89E-03 | 147.0 |
| rs7675998 | *ZNF208* | 4 | 164,227,270 | A/G | -0.074 | 0.009 | 4.35E-16 | 34,694 |  | TG | -0.010 | 0.006 | 0.131 | 91,013 | 1.94E-03 | 67.6 |
| rs2736100 | *NAF1* | 5 | 1,339,516 | A/C | -0.078 | 0.009 | 4.38E-19 | 25,842 |  | TG | -0.008 | 0.004 | 0.040 | 163,771 | 2.90E-03 | 75.1 |
| rs9420907 | *ACYP2* | 10 | 105,666,455 | A/C | -0.069 | 0.010 | 6.90E-11 | 37,653 |  | TG | 0.000 | 0.007 | 0.468 | 91,001 | 1.26E-03 | 47.6 |
| rs8105767 | *RTEL1* | 19 | 22,007,281 | A/G | -0.048 | 0.008 | 1.11E-09 | 37,499 |  | TG | -0.002 | 0.005 | 0.445 | 89,484 | 9.59E-04 | 36.0 |

Supplementary Table S5. Summary information of instrumental variables for LTL and lipids in the European population. Note: SNP: the label of single-nucleotide polymorphism; CHR: chromosome; BP: base position; A1: effect allele, indicates the allele that is associated with shorter LTL, explaining why all the BETA estimates are negative; A2: alternative allele; BETA: SNP effect size; SE: standard error of the SNP effect size; PVE: proportion of variance explained by the SNP; *p*, *N*, and *F* represent *p* value, sample size, and *F* statistic, respectively. For each lipid, all the selected instruments collectively explain about 1.18% phenotypic variation of LTL at the observed scale. For these instrumental variables, all the *F* statistics are above 10 (ranging from 31.4 to 147.0) with an average *F* statistic of 67.4 and an overall *F* statistic of 69.9, suggesting that the selected genetic variants have sufficient strong effects for instrument and the bias of weak instrument is unlikely.

| SNP | GENE | CHR | BP | A1/A2 | LTL | | | |  | lipids | | | | | PVE | *F* |
| --- | --- | --- | --- | --- | --- | --- | --- | --- | --- | --- | --- | --- | --- | --- | --- | --- |
|  |  |  |  |  | BETA | SE | *p* | *N* |  | Subtype | BETA | SE | *p* | *N* |  |  |
| rs3219104 | *PARP1* | 1 | 226,562,621 | A/C | -0.074 | 0.009 | 2.23E-16 | 23,096 |  | HDL | -0.014 | 0.006 | 0.013 | 70,657 | 2.92E-03 | 67.6 |
| rs2293607 | *TERC* | 3 | 169,482,335 | C/T | -0.120 | 0.009 | 7.57E-39 | 23,096 |  | HDL | -0.002 | 0.006 | 0.665 | 70,657 | 7.64E-03 | 177.8 |
| rs10857352 | *NAF1* | 4 | 164,101,482 | A/G | -0.064 | 0.011 | 4.85E-09 | 23,096 |  | HDL | 0.011 | 0.007 | 0.127 | 70,657 | 1.46E-03 | 33.8 |
| rs7705526 | *TERT* | 5 | 1,285,974 | C/A | -0.118 | 0.009 | 2.61E-38 | 23,096 |  | HDL | -0.011 | 0.006 | 0.078 | 70,657 | 7.39E-03 | 171.9 |
| rs7776744 | *POT1* | 7 | 124,599,749 | G/A | -0.058 | 0.009 | 2.51E-10 | 23,096 |  | HDL | 0.004 | 0.006 | 0.539 | 70,657 | 1.79E-03 | 41.5 |
| rs12415148 | *OBFC1* | 10 | 105,680,586 | T/C | -0.204 | 0.020 | 2.78E-25 | 23,096 |  | HDL | 0.001 | 0.011 | 0.895 | 70,657 | 4.48E-03 | 104.0 |
| rs41293836 | *TINF2* | 14 | 24,721,327 | C/T | -0.233 | 0.017 | 2.47E-42 | 23,096 |  | HDL | -0.006 | 0.011 | 0.581 | 70,657 | 8.07E-03 | 187.8 |
| rs41309367 | *RTEL1* | 20 | 62,309,554 | T/C | -0.058 | 0.010 | 1.16E-08 | 23,096 |  | HDL | 0.014 | 0.006 | 0.019 | 70,657 | 1.45E-03 | 33.6 |
| rs3219104 | *PARP1* | 1 | 226,562,621 | A/C | -0.074 | 0.009 | 2.23E-16 | 23,096 |  | LDL | -0.007 | 0.006 | 0.232 | 72,866 | 2.92E-03 | 67.6 |
| rs2293607 | *TERC* | 3 | 169,482,335 | C/T | -0.120 | 0.009 | 7.57E-39 | 23,096 |  | LDL | 0.007 | 0.006 | 0.221 | 72,866 | 7.64E-03 | 177.8 |
| rs10857352 | *NAF1* | 4 | 164,101,482 | A/G | -0.064 | 0.011 | 4.85E-09 | 23,096 |  | LDL | -0.004 | 0.007 | 0.588 | 72,866 | 1.46E-03 | 33.8 |
| rs7705526 | *TERT* | 5 | 1,285,974 | C/A | -0.118 | 0.009 | 2.61E-38 | 23,096 |  | LDL | -0.014 | 0.007 | 0.031 | 72,866 | 7.39E-03 | 171.9 |
| rs7776744 | *POT1* | 7 | 124,599,749 | G/A | -0.058 | 0.009 | 2.51E-10 | 23,096 |  | LDL | 0.011 | 0.006 | 0.073 | 72,866 | 1.79E-03 | 41.5 |
| rs12415148 | *OBFC1* | 10 | 105,680,586 | T/C | -0.204 | 0.020 | 2.78E-25 | 23,096 |  | LDL | 0.014 | 0.011 | 0.207 | 72,866 | 4.48E-03 | 104.0 |
| rs41293836 | *TINF2* | 14 | 24,721,327 | C/T | -0.233 | 0.017 | 2.47E-42 | 23,096 |  | LDL | 0.006 | 0.012 | 0.619 | 72,866 | 8.07E-03 | 187.8 |
| rs41309367 | *RTEL1* | 20 | 62,309,554 | T/C | -0.058 | 0.010 | 1.16E-08 | 23,096 |  | LDL | -0.007 | 0.006 | 0.271 | 72,866 | 1.45E-03 | 33.6 |
| rs3219104 | *PARP1* | 1 | 226,562,621 | A/C | -0.074 | 0.009 | 2.23E-16 | 23,096 |  | TC | -0.003 | 0.004 | 0.412 | 128,305 | 2.92E-03 | 67.6 |
| rs2293607 | *TERC* | 3 | 169,482,335 | C/T | -0.120 | 0.009 | 7.57E-39 | 23,096 |  | TC | 0.000 | 0.004 | 0.957 | 128,305 | 7.64E-03 | 177.8 |
| rs10857352 | *NAF1* | 4 | 164,101,482 | A/G | -0.064 | 0.011 | 4.85E-09 | 23,096 |  | TC | 0.000 | 0.004 | 0.979 | 128,305 | 1.46E-03 | 33.8 |
| rs7705526 | *TERT* | 5 | 1,285,974 | C/A | -0.118 | 0.009 | 2.61E-38 | 23,096 |  | TC | -0.009 | 0.004 | 0.033 | 128,305 | 7.39E-03 | 171.9 |
| rs7776744 | *POT1* | 7 | 124,599,749 | G/A | -0.058 | 0.009 | 2.51E-10 | 23,096 |  | TC | 0.004 | 0.004 | 0.269 | 128,305 | 1.79E-03 | 41.5 |
| rs12415148 | *OBFC1* | 10 | 105,680,586 | T/C | -0.204 | 0.020 | 2.78E-25 | 23,096 |  | TC | 0.016 | 0.007 | 0.016 | 128,305 | 4.48E-03 | 104.0 |
| rs41293836 | *TINF2* | 14 | 24,721,327 | C/T | -0.233 | 0.017 | 2.47E-42 | 23,096 |  | TC | -0.007 | 0.007 | 0.302 | 128,305 | 8.07E-03 | 187.8 |
| rs41309367 | *RTEL1* | 20 | 62,309,554 | T/C | -0.058 | 0.010 | 1.16E-08 | 23,096 |  | TC | 0.004 | 0.004 | 0.338 | 128,305 | 1.45E-03 | 33.6 |
| rs3219104 | *PARP1* | 1 | 226,562,621 | A/C | -0.074 | 0.009 | 2.23E-16 | 23,096 |  | TG | 0.003 | 0.004 | 0.433 | 105,597 | 2.92E-03 | 67.6 |
| rs2293607 | *TERC* | 3 | 169,482,335 | C/T | -0.120 | 0.009 | 7.57E-39 | 23,096 |  | TG | 0.002 | 0.004 | 0.643 | 105,597 | 7.64E-03 | 177.8 |
| rs10857352 | *NAF1* | 4 | 164,101,482 | A/G | -0.064 | 0.011 | 4.85E-09 | 23,096 |  | TG | 0.004 | 0.005 | 0.384 | 105,597 | 1.46E-03 | 33.8 |
| rs7705526 | *TERT* | 5 | 1,285,974 | C/A | -0.118 | 0.009 | 2.61E-38 | 23,096 |  | TG | 0.003 | 0.004 | 0.472 | 105,597 | 7.39E-03 | 171.9 |
| rs7776744 | *POT1* | 7 | 124,599,749 | G/A | -0.058 | 0.009 | 2.51E-10 | 23,096 |  | TG | 0.002 | 0.004 | 0.509 | 105,597 | 1.79E-03 | 41.5 |
| rs12415148 | *OBFC1* | 10 | 105,680,586 | T/C | -0.204 | 0.020 | 2.78E-25 | 23,096 |  | TG | 0.005 | 0.007 | 0.503 | 105,597 | 4.48E-03 | 104.0 |
| rs41293836 | *TINF2* | 14 | 24,721,327 | C/T | -0.233 | 0.017 | 2.47E-42 | 23,096 |  | TG | 0.001 | 0.007 | 0.938 | 105,597 | 8.07E-03 | 187.8 |
| rs41309367 | *RTEL1* | 20 | 62,309,554 | T/C | -0.058 | 0.010 | 1.16E-08 | 23,096 |  | TG | -0.002 | 0.004 | 0.515 | 105,597 | 1.45E-03 | 33.6 |

Supplementary Table S6. Summary information of instrumental variables for LTL and lipids in the Asian population. Note: SNP: the label of single-nucleotide polymorphism; CHR: chromosome; BP: base position; A1: effect allele, indicates the allele that is associated with shorter LTL, explaining why all the BETA estimates are negative; A2: alternative allele; BETA: SNP effect size; SE: standard error of the SNP effect size; PVE: proportion of variance explained by the SNP; *p*, *N*, and *F* represent *p* value, sample size, and *F* statistic, respectively. BETA and SE of the index SNPs of HDL, LDL, TC and TG are recomputed by multiplying a factor of 1.05 (= 15.4/14.7), 1.11 (= 41.0/37.0), 0.91 (= 38.6/42.6) and 0.83 (= 71.9/86.8), respectively. For each lipid, all the selected instruments collectively explain about 3.52% phenotypic variation of LTL at the observed scale. For these instrumental variables, all the *F* statistics are above 10 (ranging from 33.6 to 187.8) with an average *F* statistic of 102.3 and an overall *F* statistic of 105.3, suggesting that the selected genetic variants have sufficient strong effects for instrument and the bias of weak instrument is unlikely.

| SNP | GENE | CHR | BP | A1/A2 | lipids | | | | |  | ALS | | | | PVE | *F* |
| --- | --- | --- | --- | --- | --- | --- | --- | --- | --- | --- | --- | --- | --- | --- | --- | --- |
|  |  |  |  |  | Subtype | BETA | SE | *p* | *N* |  | BETA | SE | *p* | *N* |  |  |
| rs12133576 | *DR1* | 1 | 93,816,400 | A/G | HDL | 0.024 | 0.004 | 6.15E-11 | 187,123 |  | 0.013 | 0.014 | 0.374 | 80,610 | 2.58E-04 | 48.2 |
| rs12145743 | *RRNAD1* | 1 | 156,700,651 | G/T | HDL | 0.020 | 0.004 | 1.80E-08 | 181,336 |  | -0.016 | 0.014 | 0.258 | 80,610 | 1.75E-04 | 31.8 |
| rs12740374 | *CELSR2* | 1 | 109,817,590 | T/G | HDL | 0.034 | 0.004 | 1.69E-15 | 186,888 |  | -0.015 | 0.017 | 0.365 | 80,610 | 3.74E-04 | 70.0 |
| rs12748152 | *AL034380.1* | 1 | 27,138,393 | C/T | HDL | 0.051 | 0.006 | 9.74E-16 | 187,057 |  | 0.017 | 0.026 | 0.509 | 80,610 | 3.56E-04 | 66.6 |
| rs1689797 | *GS1-122H1.2* | 1 | 182,150,978 | C/A | HDL | 0.036 | 0.004 | 2.85E-21 | 187,126 |  | 0.023 | 0.014 | 0.107 | 80,610 | 5.28E-04 | 98.9 |
| rs2642438 | *MARC1* | 1 | 220,970,028 | G/A | HDL | 0.030 | 0.004 | 7.78E-14 | 179,439 |  | 0.001 | 0.015 | 0.957 | 80,610 | 3.36E-04 | 60.4 |
| rs4650994 | *C1orf220* | 1 | 178,515,312 | G/A | HDL | 0.021 | 0.003 | 6.70E-09 | 186,927 |  | -0.003 | 0.014 | 0.849 | 80,610 | 2.04E-04 | 38.1 |
| rs4660293 | *PABPC4* | 1 | 40,028,180 | A/G | HDL | 0.035 | 0.004 | 2.86E-18 | 187,027 |  | -0.014 | 0.016 | 0.407 | 80,610 | 4.16E-04 | 77.9 |
| rs4846914 | *GALNT2* | 1 | 230,295,691 | A/G | HDL | 0.048 | 0.003 | 3.51E-41 | 186,995 |  | -0.018 | 0.014 | 0.188 | 80,610 | 1.06E-03 | 198.5 |
| rs1047891 | *CPS1* | 2 | 211,540,507 | C/A | HDL | 0.027 | 0.004 | 8.73E-10 | 182,043 |  | 0.001 | 0.015 | 0.930 | 80,610 | 2.61E-04 | 47.6 |
| rs1515110 | *AC068138.1* | 2 | 227,122,216 | G/T | HDL | 0.032 | 0.004 | 8.04E-18 | 187,081 |  | 0.025 | 0.014 | 0.076 | 80,610 | 4.55E-04 | 85.2 |
| rs676210 | *APOB* | 2 | 21,231,524 | A/G | HDL | 0.066 | 0.004 | 2.35E-54 | 187,081 |  | -0.038 | 0.016 | 0.020 | 80,610 | 1.45E-03 | 272.2 |
| rs7607980 | *COBLL1* | 2 | 165,551,201 | C/T | HDL | 0.045 | 0.005 | 1.81E-15 | 187,036 |  | -0.005 | 0.021 | 0.795 | 80,610 | 3.95E-04 | 73.9 |
| rs13076253 | *CPNE4* | 3 | 131,751,775 | A/C | HDL | 0.028 | 0.005 | 4.96E-09 | 186,809 |  | -0.043 | 0.019 | 0.027 | 80,610 | 1.86E-04 | 34.8 |
| rs13099479 | *PBRM1* | 3 | 52,677,478 | A/G | HDL | 0.036 | 0.006 | 1.82E-08 | 187,132 |  | -0.003 | 0.024 | 0.915 | 80,610 | 1.80E-04 | 33.7 |
| rs2013208 | *RBM5* | 3 | 50,129,399 | T/C | HDL | 0.025 | 0.004 | 8.92E-12 | 169,708 |  | -0.014 | 0.014 | 0.291 | 80,610 | 2.93E-04 | 49.8 |
| rs2606736 | *ATG7* | 3 | 11,400,249 | C/T | HDL | 0.025 | 0.004 | 4.80E-08 | 129,328 |  | 0.009 | 0.014 | 0.539 | 80,610 | 2.53E-04 | 32.7 |
| rs6805251 | *GSK3B* | 3 | 119,560,606 | T/C | HDL | 0.020 | 0.004 | 1.33E-08 | 186,301 |  | -0.009 | 0.014 | 0.504 | 80,610 | 1.75E-04 | 32.7 |
| rs687339 | *RP11-463H24.1* | 3 | 135,932,359 | C/T | HDL | 0.032 | 0.004 | 7.11E-13 | 187,105 |  | 0.010 | 0.016 | 0.558 | 80,610 | 3.02E-04 | 56.6 |
| rs10019888 | *RP11-324H7.1* | 4 | 26,062,990 | A/G | HDL | 0.027 | 0.005 | 4.90E-08 | 187,077 |  | 0.025 | 0.018 | 0.170 | 80,610 | 1.84E-04 | 34.5 |
| rs13107325 | *SLC39A8* | 4 | 103,188,709 | C/T | HDL | 0.071 | 0.008 | 1.07E-15 | 179,316 |  | -0.045 | 0.026 | 0.089 | 80,610 | 4.59E-04 | 82.4 |
| rs2602836 | *RP11-696N14.1* | 4 | 100,014,805 | A/G | HDL | 0.019 | 0.003 | 4.96E-08 | 187,102 |  | -0.014 | 0.014 | 0.293 | 80,610 | 1.70E-04 | 31.9 |
| rs3822072 | *FAM13A* | 4 | 89,741,269 | G/A | HDL | 0.025 | 0.003 | 4.06E-12 | 187,115 |  | 0.019 | 0.014 | 0.190 | 80,610 | 2.91E-04 | 54.5 |
| rs6450176 | *ARL15* | 5 | 53,298,025 | G/A | HDL | 0.025 | 0.004 | 6.88E-10 | 187,132 |  | -0.024 | 0.016 | 0.131 | 80,610 | 2.27E-04 | 42.4 |
| rs1936800 | *RP11-73O6.4* | 6 | 127,436,064 | C/T | HDL | 0.020 | 0.003 | 3.06E-10 | 187,111 |  | 0.023 | 0.014 | 0.093 | 80,610 | 1.85E-04 | 34.6 |
| rs1980493 | *BTNL2* | 6 | 32,363,215 | T/C | HDL | 0.032 | 0.005 | 3.76E-10 | 183,275 |  | 0.024 | 0.020 | 0.213 | 80,610 | 2.39E-04 | 43.9 |
| rs205262 | *C6orf106* | 6 | 34,563,164 | A/G | HDL | 0.028 | 0.004 | 3.88E-13 | 181,707 |  | 0.002 | 0.015 | 0.908 | 80,610 | 2.90E-04 | 52.7 |
| rs3861397 | *RP11-12A2.3* | 6 | 139,828,916 | A/G | HDL | 0.024 | 0.004 | 8.40E-11 | 187,084 |  | 0.012 | 0.015 | 0.398 | 80,610 | 2.38E-04 | 44.4 |
| rs9457931 | *LPAL2* | 6 | 160,929,904 | A/G | HDL | 0.055 | 0.007 | 7.30E-13 | 171,669 |  | -0.007 | 0.030 | 0.816 | 80,610 | 3.33E-04 | 57.2 |
| rs998584 | *VEGFA* | 6 | 43,757,896 | C/A | HDL | 0.026 | 0.004 | 2.27E-11 | 183,791 |  | 0.002 | 0.015 | 0.911 | 80,610 | 2.55E-04 | 46.8 |
| rs11765979 | *KLF14* | 7 | 130,445,877 | C/A | HDL | 0.041 | 0.005 | 3.11E-17 | 94,311 |  | 0.021 | 0.014 | 0.137 | 80,610 | 7.81E-04 | 73.7 |
| rs17145738 | *TBL2* | 7 | 72,982,874 | T/C | HDL | 0.041 | 0.005 | 4.95E-13 | 184,971 |  | -0.005 | 0.022 | 0.809 | 80,610 | 3.20E-04 | 59.3 |
| rs17173637 | *ABP1* | 7 | 150,529,449 | T/C | HDL | 0.036 | 0.006 | 1.90E-08 | 183,901 |  | -0.014 | 0.024 | 0.547 | 80,610 | 2.20E-04 | 40.6 |
| rs4142995 | *SNX13* | 7 | 17,919,258 | G/T | HDL | 0.026 | 0.004 | 9.37E-12 | 165,161 |  | 0.002 | 0.014 | 0.860 | 80,610 | 3.06E-04 | 50.5 |
| rs4917014 | *AC020743.4* | 7 | 50,305,863 | G/T | HDL | 0.022 | 0.004 | 1.03E-08 | 186,868 |  | 0.000 | 0.015 | 0.999 | 80,610 | 2.03E-04 | 38.0 |
| rs702485 | *DAGLB* | 7 | 6,449,272 | G/A | HDL | 0.024 | 0.003 | 6.45E-12 | 186,974 |  | 0.002 | 0.014 | 0.915 | 80,610 | 2.73E-04 | 51.1 |
| rs10087900 | *GPIHBP1* | 8 | 144,303,418 | G/A | HDL | 0.023 | 0.004 | 2.17E-09 | 183,672 |  | 0.019 | 0.014 | 0.168 | 80,610 | 2.24E-04 | 41.2 |
| rs10808546 | *RP11-136O12.2* | 8 | 126,495,818 | T/C | HDL | 0.041 | 0.003 | 4.11E-30 | 185,835 |  | -0.008 | 0.014 | 0.571 | 80,610 | 7.78E-04 | 144.7 |
| rs13702 | *LPL* | 8 | 19,824,492 | C/T | HDL | 0.106 | 0.004 | 1.28E-160 | 187,044 |  | -0.010 | 0.015 | 0.499 | 80,610 | 4.13E-03 | 775.2 |
| rs1866956 | *AC107964.1* | 8 | 19,748,921 | T/C | HDL | 0.022 | 0.004 | 7.96E-10 | 187,031 |  | 0.004 | 0.015 | 0.796 | 80,610 | 1.84E-04 | 34.4 |
| rs2293889 | *TRPS1* | 8 | 116,599,199 | G/T | HDL | 0.031 | 0.004 | 4.27E-17 | 180,102 |  | 0.022 | 0.014 | 0.112 | 80,610 | 4.41E-04 | 79.5 |
| rs4240624 | *RP11-115J16.1* | 8 | 9,184,231 | A/G | HDL | 0.082 | 0.006 | 1.32E-45 | 185,696 |  | -0.022 | 0.025 | 0.369 | 80,610 | 1.07E-03 | 198.9 |
| rs11789603 | *ABCA1* | 9 | 107,647,019 | T/C | HDL | 0.060 | 0.006 | 3.70E-21 | 184,432 |  | -0.003 | 0.022 | 0.899 | 80,610 | 5.42E-04 | 100.0 |
| rs1883025 | *ABCA1* | 9 | 107,664,301 | C/T | HDL | 0.070 | 0.004 | 1.50E-65 | 186,365 |  | -0.013 | 0.016 | 0.408 | 80,610 | 1.55E-03 | 289.8 |
| rs2066714 | *ABCA1* | 9 | 107,586,753 | C/T | HDL | 0.045 | 0.007 | 7.26E-10 | 93,528 |  | -0.001 | 0.020 | 0.957 | 80,610 | 4.35E-04 | 40.7 |
| rs686030 | *TTC39B* | 9 | 15,304,782 | A/C | HDL | 0.055 | 0.005 | 4.29E-27 | 187,035 |  | 0.057 | 0.020 | 0.004 | 80,610 | 6.73E-04 | 126.0 |
| rs10761771 | *RP11-351O1.3* | 10 | 65,230,164 | C/T | HDL | 0.020 | 0.003 | 4.12E-09 | 182,895 |  | -0.004 | 0.014 | 0.765 | 80,610 | 1.85E-04 | 33.9 |
| rs12412743 | *TECTB* | 10 | 114,045,333 | C/T | HDL | 0.029 | 0.005 | 1.31E-09 | 187,095 |  | 0.021 | 0.018 | 0.242 | 80,610 | 2.23E-04 | 41.8 |
| rs2250802 | *GPAM* | 10 | 113,921,354 | G/A | HDL | 0.034 | 0.004 | 2.02E-17 | 184,088 |  | 0.029 | 0.015 | 0.050 | 80,610 | 4.35E-04 | 80.1 |
| rs970548 | *MARCH8* | 10 | 46,013,277 | C/A | HDL | 0.026 | 0.004 | 1.71E-10 | 186,596 |  | 0.000 | 0.016 | 0.996 | 80,610 | 2.34E-04 | 43.8 |
| rs102275 | *C11orf10* | 11 | 61,557,803 | T/C | HDL | 0.039 | 0.004 | 6.40E-28 | 187,085 |  | 0.004 | 0.014 | 0.778 | 80,610 | 6.67E-04 | 124.8 |
| rs10501321 | *MADD* | 11 | 47,294,626 | C/T | HDL | 0.048 | 0.004 | 3.54E-38 | 186,984 |  | 0.025 | 0.015 | 0.091 | 80,610 | 9.62E-04 | 180.0 |
| rs12801636 | *PCNXL3* | 11 | 65,391,317 | A/G | HDL | 0.024 | 0.004 | 3.15E-08 | 187,099 |  | -0.007 | 0.016 | 0.652 | 80,610 | 1.67E-04 | 31.3 |
| rs499974 | *MOGAT2* | 11 | 75,455,021 | C/A | HDL | 0.026 | 0.004 | 1.12E-08 | 186,749 |  | 0.000 | 0.019 | 0.991 | 80,610 | 1.91E-04 | 35.7 |
| rs7112577 | *PAFAH1B2* | 11 | 117,044,603 | G/C | HDL | 0.083 | 0.013 | 2.34E-10 | 89,235 |  | -0.042 | 0.036 | 0.246 | 80,610 | 4.59E-04 | 41.0 |
| rs11045163 | *RP11-284H19.1* | 12 | 20,463,526 | G/A | HDL | 0.022 | 0.004 | 3.20E-09 | 187,075 |  | 0.014 | 0.014 | 0.321 | 80,610 | 2.05E-04 | 38.4 |
| rs11065987 | *BRAP* | 12 | 112,072,424 | A/G | HDL | 0.022 | 0.004 | 1.23E-09 | 187,119 |  | 0.035 | 0.014 | 0.010 | 80,610 | 2.15E-04 | 40.2 |
| rs2241210 | *UBE3B* | 12 | 109,950,144 | G/A | HDL | 0.033 | 0.004 | 2.49E-20 | 174,782 |  | -0.024 | 0.014 | 0.084 | 80,610 | 5.15E-04 | 90.0 |
| rs2454722 | *HCAR1* | 12 | 123,171,218 | G/A | HDL | 0.035 | 0.004 | 3.31E-14 | 186,355 |  | 0.023 | 0.019 | 0.230 | 80,610 | 3.41E-04 | 63.6 |
| rs4379922 | *RP11-592O2.1* | 12 | 125,351,116 | C/T | HDL | 0.025 | 0.004 | 9.56E-12 | 186,203 |  | 0.004 | 0.014 | 0.797 | 80,610 | 2.53E-04 | 47.1 |
| rs7306660 | *SCARB1* | 12 | 125,327,384 | G/A | HDL | 0.035 | 0.004 | 3.34E-19 | 186,939 |  | 0.011 | 0.015 | 0.469 | 80,610 | 4.91E-04 | 91.8 |
| rs838876 | *SCARB1* | 12 | 125,259,888 | A/G | HDL | 0.049 | 0.004 | 7.33E-33 | 173,066 |  | 0.007 | 0.016 | 0.630 | 80,610 | 9.22E-04 | 159.8 |
| rs4983559 | *ZBTB42* | 14 | 105,277,209 | G/A | HDL | 0.020 | 0.004 | 9.57E-09 | 183,672 |  | -0.003 | 0.014 | 0.858 | 80,610 | 1.63E-04 | 29.9 |
| rs10468017 | *ALDH1A2* | 15 | 58,678,512 | T/C | HDL | 0.118 | 0.004 | 1.21E-188 | 181,223 |  | -0.012 | 0.015 | 0.421 | 80,610 | 5.28E-03 | 962.6 |
| rs424346 | *ADAM10* | 15 | 59,010,962 | T/C | HDL | 0.068 | 0.011 | 4.84E-08 | 128,346 |  | 0.021 | 0.038 | 0.590 | 80,610 | 2.81E-04 | 36.1 |
| rs492571 | *FRMD5* | 15 | 44,211,273 | T/C | HDL | 0.066 | 0.009 | 1.27E-12 | 177,352 |  | 0.016 | 0.032 | 0.615 | 80,610 | 3.06E-04 | 54.3 |
| rs633695 | *LIPC* | 15 | 58,725,839 | G/A | HDL | 0.089 | 0.005 | 7.82E-58 | 92,820 |  | -0.002 | 0.015 | 0.916 | 80,610 | 2.89E-03 | 268.6 |
| rs16942887 | *PSKH1* | 16 | 67,928,042 | A/G | HDL | 0.083 | 0.005 | 8.28E-54 | 185,604 |  | -0.018 | 0.021 | 0.373 | 80,610 | 1.43E-03 | 265.5 |
| rs16965220 | *NLRC5* | 16 | 57,065,121 | A/C | HDL | 0.022 | 0.004 | 7.91E-09 | 185,512 |  | 0.011 | 0.015 | 0.432 | 80,610 | 1.89E-04 | 35.0 |
| rs2241770 | *NUP93* | 16 | 56,866,196 | T/C | HDL | 0.099 | 0.006 | 6.78E-60 | 185,537 |  | -0.021 | 0.024 | 0.370 | 80,610 | 1.62E-03 | 301.0 |
| rs2925979 | *CMIP* | 16 | 81,534,790 | C/T | HDL | 0.035 | 0.004 | 1.32E-19 | 185,553 |  | -0.003 | 0.015 | 0.863 | 80,610 | 4.85E-04 | 90.0 |
| rs9989419 | *AC012181.1* | 16 | 56,985,139 | G/A | HDL | 0.147 | 0.004 | 0.00E+00 | 177,532 |  | -0.001 | 0.015 | 0.975 | 80,610 | 9.34E-03 | 1674.2 |
| rs1877031 | *STARD3* | 17 | 37,814,080 | A/G | HDL | 0.034 | 0.004 | 1.20E-19 | 185,471 |  | -0.006 | 0.014 | 0.674 | 80,610 | 4.69E-04 | 87.1 |
| rs4148005 | *ABCA8* | 17 | 66,882,466 | T/G | HDL | 0.028 | 0.004 | 5.74E-14 | 184,431 |  | 0.004 | 0.015 | 0.803 | 80,610 | 3.35E-04 | 61.8 |
| rs4939883 | *LIPG* | 18 | 47,167,214 | C/T | HDL | 0.080 | 0.005 | 1.80E-66 | 185,576 |  | -0.023 | 0.018 | 0.202 | 80,610 | 1.70E-03 | 315.3 |
| rs6567160 | *U4* | 18 | 57,829,135 | T/C | HDL | 0.026 | 0.004 | 2.92E-09 | 185,608 |  | -0.002 | 0.016 | 0.909 | 80,610 | 2.12E-04 | 39.3 |
| rs103294 | *AC010518.3* | 19 | 54,797,848 | T/C | HDL | 0.052 | 0.004 | 4.00E-30 | 175,917 |  | -0.023 | 0.018 | 0.214 | 80,610 | 8.02E-04 | 141.3 |
| rs2075650 | *TOMM40* | 19 | 45,395,619 | A/G | HDL | 0.055 | 0.005 | 9.72E-26 | 175,421 |  | -0.036 | 0.020 | 0.077 | 80,610 | 6.72E-04 | 118.0 |
| rs2278236 | *ANGPTL4* | 19 | 8,431,581 | A/G | HDL | 0.033 | 0.004 | 3.19E-18 | 185,450 |  | 0.023 | 0.014 | 0.091 | 80,610 | 4.82E-04 | 89.4 |
| rs2288912 | *APOC4* | 19 | 45,449,199 | G/C | HDL | 0.030 | 0.004 | 7.15E-15 | 178,910 |  | 0.001 | 0.014 | 0.932 | 80,610 | 3.80E-04 | 68.1 |
| rs731839 | *PEPD* | 19 | 33,899,065 | A/G | HDL | 0.022 | 0.004 | 3.44E-09 | 185,498 |  | -0.024 | 0.015 | 0.117 | 80,610 | 1.91E-04 | 35.4 |
| rs737337 | *DOCK6* | 19 | 11,347,493 | T/C | HDL | 0.057 | 0.006 | 4.56E-17 | 185,432 |  | 0.035 | 0.025 | 0.173 | 80,610 | 4.62E-04 | 85.8 |
| rs4465830 | *ZNF335* | 20 | 44,585,420 | A/G | HDL | 0.060 | 0.004 | 5.18E-40 | 185,505 |  | 0.000 | 0.018 | 0.989 | 80,610 | 9.91E-04 | 184.1 |
| rs6031587 | *HNF4A* | 20 | 43,038,249 | C/T | HDL | 0.049 | 0.007 | 1.92E-09 | 163,095 |  | 0.002 | 0.027 | 0.950 | 80,610 | 2.67E-04 | 43.5 |
| rs181360 | *UBE2L3* | 22 | 21,928,916 | T/G | HDL | 0.038 | 0.004 | 9.24E-18 | 178,216 |  | 0.032 | 0.018 | 0.071 | 80,610 | 4.50E-04 | 80.1 |
| rs10903129 | *TMEM57* | 1 | 25,768,937 | G/A | LDL | 0.033 | 0.004 | 3.03E-17 | 169,920 |  | 0.015 | 0.014 | 0.279 | 80,610 | 4.62E-04 | 78.6 |
| rs11591147 | *PCSK9* | 1 | 55,505,647 | G/T | LDL | 0.497 | 0.018 | 8.58E-143 | 77,417 |  | -0.075 | 0.057 | 0.188 | 80,610 | 9.75E-03 | 762.4 |
| rs12748152 | *AL034380.1* | 1 | 27,138,393 | T/C | LDL | 0.050 | 0.007 | 3.21E-12 | 172,988 |  | -0.017 | 0.026 | 0.509 | 80,610 | 3.30E-04 | 57.2 |
| rs2587534 | *RP4-781K5.7* | 1 | 234,849,339 | A/G | LDL | 0.039 | 0.004 | 8.06E-25 | 172,966 |  | 0.007 | 0.014 | 0.628 | 80,610 | 6.45E-04 | 111.7 |
| rs2642438 | *MARC1* | 1 | 220,970,028 | G/A | LDL | 0.035 | 0.004 | 7.32E-16 | 165,470 |  | 0.001 | 0.015 | 0.957 | 80,610 | 4.24E-04 | 70.2 |
| rs267733 | *ANXA9* | 1 | 150,958,836 | A/G | LDL | 0.033 | 0.005 | 5.29E-09 | 164,562 |  | -0.060 | 0.019 | 0.001 | 80,610 | 2.37E-04 | 39.0 |
| rs2902875 | *RP11-101C11.1* | 1 | 55,695,535 | T/C | LDL | 0.076 | 0.013 | 2.19E-09 | 89,883 |  | 0.037 | 0.035 | 0.293 | 80,610 | 4.00E-04 | 36.0 |
| rs4970712 | *EVI5* | 1 | 92,993,547 | C/A | LDL | 0.034 | 0.004 | 2.46E-13 | 173,036 |  | -0.004 | 0.017 | 0.792 | 80,610 | 3.43E-04 | 59.4 |
| rs646776 | *PSRC1* | 1 | 109,818,530 | T/C | LDL | 0.160 | 0.004 | 1.63E-272 | 173,021 |  | 0.017 | 0.017 | 0.318 | 80,610 | 7.60E-03 | 1325.6 |
| rs7534572 | *DOCK7* | 1 | 62,999,675 | G/C | LDL | 0.041 | 0.006 | 1.29E-11 | 75,145 |  | 0.008 | 0.015 | 0.600 | 80,610 | 6.55E-04 | 49.2 |
| rs7551981 | *RP11-101C11.1* | 1 | 55,719,166 | T/G | LDL | 0.047 | 0.004 | 1.36E-33 | 173,021 |  | 0.025 | 0.014 | 0.071 | 80,610 | 8.91E-04 | 154.3 |
| rs10195252 | *COBLL1* | 2 | 165,513,091 | T/C | LDL | 0.024 | 0.004 | 3.81E-08 | 157,208 |  | -0.007 | 0.014 | 0.627 | 80,610 | 2.37E-04 | 37.2 |
| rs10490626 | *INSIG2* | 2 | 118,835,841 | G/A | LDL | 0.051 | 0.007 | 1.70E-12 | 173,044 |  | 0.021 | 0.026 | 0.411 | 80,610 | 3.13E-04 | 54.2 |
| rs11563251 | *UGT1A1* | 2 | 234,679,384 | T/C | LDL | 0.035 | 0.006 | 4.50E-08 | 172,855 |  | -0.031 | 0.023 | 0.174 | 80,610 | 1.79E-04 | 31.0 |
| rs1250229 | *AC012462.1* | 2 | 216,304,384 | C/T | LDL | 0.024 | 0.004 | 3.13E-08 | 173,032 |  | -0.025 | 0.016 | 0.111 | 80,610 | 1.93E-04 | 33.5 |
| rs1367117 | *APOB* | 2 | 21,263,900 | A/G | LDL | 0.119 | 0.004 | 9.48E-183 | 173,007 |  | 0.035 | 0.015 | 0.017 | 80,610 | 5.06E-03 | 879.1 |
| rs16831243 | *YSK4* | 2 | 135,762,344 | T/C | LDL | 0.038 | 0.006 | 9.06E-12 | 162,945 |  | 0.023 | 0.024 | 0.342 | 80,610 | 2.90E-04 | 47.2 |
| rs2030746 | *AC073257.1* | 2 | 121,309,488 | T/C | LDL | 0.021 | 0.004 | 8.61E-09 | 173,024 |  | -0.005 | 0.014 | 0.696 | 80,610 | 1.83E-04 | 31.7 |
| rs2710642 | *EHBP1* | 2 | 63,149,557 | A/G | LDL | 0.024 | 0.004 | 6.09E-09 | 172,994 |  | 0.009 | 0.014 | 0.535 | 80,610 | 2.29E-04 | 39.6 |
| rs6544713 | *ABCG8* | 2 | 44,073,881 | T/C | LDL | 0.081 | 0.004 | 4.84E-83 | 172,940 |  | 0.010 | 0.015 | 0.527 | 80,610 | 2.23E-03 | 386.5 |
| rs6709904 | *ABCG8* | 2 | 44,080,324 | A/G | LDL | 0.055 | 0.009 | 4.58E-10 | 89,852 |  | -0.024 | 0.021 | 0.261 | 80,610 | 4.66E-04 | 41.9 |
| rs72902576 | *APOB* | 2 | 21,275,480 | T/G | LDL | 0.093 | 0.013 | 9.58E-12 | 82,068 |  | 0.032 | 0.037 | 0.389 | 80,610 | 5.99E-04 | 49.2 |
| rs17404153 | *DNAJC13* | 3 | 132,163,200 | G/T | LDL | 0.034 | 0.005 | 1.83E-09 | 172,898 |  | -0.009 | 0.021 | 0.683 | 80,610 | 2.24E-04 | 38.7 |
| rs7640978 | *CMTM6* | 3 | 32,533,010 | C/T | LDL | 0.039 | 0.007 | 9.84E-09 | 172,228 |  | 0.011 | 0.024 | 0.645 | 80,610 | 1.87E-04 | 32.3 |
| rs9875338 | *GSTM5P1* | 3 | 12,296,469 | G/A | LDL | 0.027 | 0.004 | 2.21E-11 | 172,895 |  | 0.015 | 0.014 | 0.286 | 80,610 | 3.08E-04 | 53.2 |
| rs6818397 | *RGS12* | 4 | 3,434,885 | T/G | LDL | 0.022 | 0.004 | 1.68E-08 | 172,685 |  | 0.004 | 0.014 | 0.762 | 80,610 | 1.82E-04 | 31.4 |
| rs12916 | *HMGCR* | 5 | 74,656,539 | C/T | LDL | 0.073 | 0.004 | 7.79E-78 | 168,357 |  | -0.006 | 0.014 | 0.668 | 80,610 | 2.21E-03 | 372.1 |
| rs4530754 | *CSNK1G3* | 5 | 122,855,416 | A/G | LDL | 0.028 | 0.004 | 3.58E-12 | 173,003 |  | 0.005 | 0.014 | 0.702 | 80,610 | 3.37E-04 | 58.4 |
| rs6882076 | *TIMD4* | 5 | 156,390,297 | C/T | LDL | 0.046 | 0.004 | 3.31E-31 | 173,006 |  | 0.011 | 0.014 | 0.434 | 80,610 | 8.32E-04 | 144.0 |
| rs10947332 | *MTCO3P1* | 6 | 32,677,440 | A/G | LDL | 0.050 | 0.006 | 6.97E-18 | 169,263 |  | 0.025 | 0.021 | 0.231 | 80,610 | 4.78E-04 | 81.0 |
| rs112201728 | *SLC22A1* | 6 | 160,551,486 | T/C | LDL | 0.068 | 0.010 | 8.51E-10 | 83,124 |  | 0.020 | 0.027 | 0.450 | 80,610 | 5.07E-04 | 42.1 |
| rs13206249 | *MRPL42P2* | 6 | 16,175,022 | G/A | LDL | 0.038 | 0.006 | 4.53E-08 | 87,149 |  | 0.023 | 0.017 | 0.194 | 80,610 | 4.26E-04 | 37.2 |
| rs1408272 | *SLC17A3* | 6 | 25,842,951 | T/G | LDL | 0.052 | 0.008 | 3.68E-09 | 167,888 |  | 0.031 | 0.030 | 0.298 | 80,610 | 2.34E-04 | 39.3 |
| rs1564348 | *SLC22A1* | 6 | 160,578,860 | C/T | LDL | 0.048 | 0.005 | 2.76E-21 | 172,989 |  | 0.015 | 0.019 | 0.438 | 80,610 | 5.35E-04 | 92.5 |
| rs16891156 | *SLC22A2* | 6 | 160,608,804 | C/A | LDL | 0.097 | 0.017 | 8.23E-09 | 90,466 |  | 0.064 | 0.052 | 0.216 | 80,610 | 3.52E-04 | 31.8 |
| rs2315065 | *RP1-81D8.3* | 6 | 161,108,144 | A/C | LDL | 0.110 | 0.016 | 5.23E-12 | 67,446 |  | 0.043 | 0.027 | 0.114 | 80,610 | 7.21E-04 | 48.6 |
| rs3757354 | *MYLIP* | 6 | 16,127,407 | C/T | LDL | 0.038 | 0.004 | 2.09E-17 | 172,987 |  | -0.010 | 0.017 | 0.570 | 80,610 | 4.36E-04 | 75.4 |
| rs6909746 | *FRK* | 6 | 116,352,750 | C/T | LDL | 0.026 | 0.004 | 7.86E-11 | 170,097 |  | 0.001 | 0.014 | 0.962 | 80,610 | 2.97E-04 | 50.5 |
| rs2073547 | *NPC1L1* | 7 | 44,582,331 | G/A | LDL | 0.049 | 0.005 | 1.92E-21 | 169,889 |  | 0.006 | 0.018 | 0.728 | 80,610 | 5.76E-04 | 98.0 |
| rs2390536 | *SP4* | 7 | 21,485,397 | A/G | LDL | 0.022 | 0.004 | 2.04E-08 | 172,981 |  | 0.006 | 0.014 | 0.695 | 80,610 | 1.99E-04 | 34.4 |
| rs4722551 | *MIR148A* | 7 | 25,991,826 | C/T | LDL | 0.039 | 0.005 | 3.95E-14 | 172,946 |  | -0.002 | 0.018 | 0.936 | 80,610 | 3.68E-04 | 63.7 |
| rs13277801 | *UBXN2B* | 8 | 59,353,534 | C/T | LDL | 0.034 | 0.004 | 3.99E-17 | 173,010 |  | 0.002 | 0.014 | 0.888 | 80,610 | 4.57E-04 | 79.1 |
| rs2737252 | *TRPS1* | 8 | 116,663,898 | G/A | LDL | 0.031 | 0.004 | 7.04E-14 | 172,950 |  | 0.025 | 0.016 | 0.114 | 80,610 | 3.39E-04 | 58.7 |
| rs2954029 | *RP11-136O12.2* | 8 | 126,490,972 | A/T | LDL | 0.056 | 0.004 | 2.10E-50 | 172,963 |  | 0.002 | 0.014 | 0.892 | 80,610 | 1.42E-03 | 245.4 |
| rs7832643 | *PLEC* | 8 | 145,022,657 | T/G | LDL | 0.034 | 0.004 | 2.67E-17 | 164,854 |  | 0.011 | 0.014 | 0.456 | 80,610 | 4.83E-04 | 79.6 |
| rs9987289 | *RP11-115J16.1* | 8 | 9,183,358 | G/A | LDL | 0.071 | 0.007 | 8.53E-24 | 160,102 |  | -0.023 | 0.025 | 0.358 | 80,610 | 7.30E-04 | 117.0 |
| rs1883025 | *ABCA1* | 9 | 107,664,301 | C/T | LDL | 0.030 | 0.004 | 6.14E-11 | 172,330 |  | -0.013 | 0.016 | 0.408 | 80,610 | 2.63E-04 | 45.3 |
| rs3780181 | *VLDLR* | 9 | 2,640,759 | A/G | LDL | 0.045 | 0.007 | 1.76E-09 | 171,976 |  | 0.083 | 0.029 | 0.005 | 80,610 | 2.10E-04 | 36.2 |
| rs579459 | *ABO* | 9 | 136,154,168 | C/T | LDL | 0.067 | 0.005 | 2.42E-44 | 172,706 |  | 0.033 | 0.017 | 0.045 | 80,610 | 1.26E-03 | 218.4 |
| rs2419604 | *GPAM* | 10 | 113,944,271 | A/G | LDL | 0.030 | 0.004 | 7.49E-14 | 172,807 |  | 0.033 | 0.015 | 0.026 | 80,610 | 3.30E-04 | 57.0 |
| rs10832962 | *SPTY2D1* | 11 | 18,656,271 | T/C | LDL | 0.032 | 0.004 | 6.62E-14 | 172,920 |  | -0.001 | 0.016 | 0.944 | 80,610 | 3.70E-04 | 64.0 |
| rs10893499 | *ST3GAL4* | 11 | 126,241,979 | A/G | LDL | 0.052 | 0.005 | 3.86E-21 | 172,980 |  | -0.044 | 0.020 | 0.029 | 80,610 | 5.58E-04 | 96.6 |
| rs174583 | *FADS1* | 11 | 61,609,750 | C/T | LDL | 0.052 | 0.004 | 7.00E-41 | 172,982 |  | -0.003 | 0.014 | 0.856 | 80,610 | 1.09E-03 | 188.7 |
| rs964184 | *ZNF259* | 11 | 116,648,917 | G/C | LDL | 0.086 | 0.008 | 2.01E-26 | 89,866 |  | 0.037 | 0.020 | 0.064 | 80,610 | 1.34E-03 | 120.2 |
| rs1169288 | *HNF1A-AS1* | 12 | 121,416,650 | C/A | LDL | 0.038 | 0.004 | 6.45E-21 | 163,086 |  | 0.006 | 0.015 | 0.696 | 80,610 | 5.39E-04 | 87.9 |
| rs3184504 | *SH2B3* | 12 | 111,884,608 | C/T | LDL | 0.027 | 0.004 | 4.20E-12 | 164,996 |  | 0.046 | 0.014 | 0.001 | 80,610 | 3.01E-04 | 49.7 |
| rs4942486 | *BRCA2* | 13 | 32,953,388 | T/C | LDL | 0.024 | 0.004 | 2.26E-11 | 171,930 |  | -0.020 | 0.014 | 0.139 | 80,610 | 2.51E-04 | 43.1 |
| rs8017377 | *NYNRIN* | 14 | 24,883,887 | A/G | LDL | 0.030 | 0.004 | 2.52E-15 | 172,866 |  | -0.013 | 0.014 | 0.336 | 80,610 | 3.68E-04 | 63.6 |
| rs2000999 | *TXNL4B* | 16 | 72,108,093 | A/G | LDL | 0.065 | 0.005 | 4.22E-41 | 171,510 |  | 0.014 | 0.017 | 0.427 | 80,610 | 1.16E-03 | 199.7 |
| rs247616 | *AC012181.1* | 16 | 56,989,590 | C/T | LDL | 0.055 | 0.004 | 2.57E-37 | 171,458 |  | 0.015 | 0.015 | 0.310 | 80,610 | 1.04E-03 | 178.0 |
| rs1801689 | *APOH* | 17 | 64,210,580 | C/A | LDL | 0.103 | 0.014 | 9.81E-12 | 111,143 |  | -0.033 | 0.047 | 0.487 | 80,610 | 4.92E-04 | 54.7 |
| rs2886232 | *ABCA10* | 17 | 67,150,176 | T/C | LDL | 0.045 | 0.006 | 3.88E-11 | 162,498 |  | 0.024 | 0.023 | 0.297 | 80,610 | 3.06E-04 | 49.7 |
| rs314253 | *ASGR1* | 17 | 7,091,650 | T/C | LDL | 0.024 | 0.004 | 3.44E-10 | 169,706 |  | -0.020 | 0.014 | 0.173 | 80,610 | 2.39E-04 | 40.6 |
| rs6504872 | *C17orf57* | 17 | 45,438,952 | T/C | LDL | 0.027 | 0.004 | 3.48E-13 | 171,519 |  | -0.038 | 0.014 | 0.006 | 80,610 | 3.20E-04 | 54.8 |
| rs12721109 | *APOC4* | 19 | 45,447,221 | G/A | LDL | 0.446 | 0.018 | 2.99E-122 | 99,409 |  | 0.037 | 0.055 | 0.499 | 80,610 | 5.94E-03 | 594.5 |
| rs2228603 | *NCAN* | 19 | 19,329,924 | C/T | LDL | 0.104 | 0.007 | 4.43E-44 | 158,643 |  | 0.021 | 0.029 | 0.470 | 80,610 | 1.31E-03 | 208.6 |
| rs2965157 | *CEACAM19* | 19 | 45,176,340 | T/C | LDL | 0.189 | 0.011 | 7.29E-62 | 170,260 |  | 0.011 | 0.046 | 0.813 | 80,610 | 1.66E-03 | 283.6 |
| rs6511720 | *LDLR* | 19 | 11,202,306 | G/T | LDL | 0.221 | 0.006 | 3.85E-262 | 170,608 |  | 0.011 | 0.021 | 0.608 | 80,610 | 7.63E-03 | 1311.4 |
| rs676388 | *MAMSTR* | 19 | 49,211,969 | C/T | LDL | 0.027 | 0.004 | 1.31E-11 | 166,830 |  | -0.006 | 0.014 | 0.656 | 80,610 | 2.77E-04 | 46.2 |
| rs7254892 | *PVRL2* | 19 | 45,389,596 | G/A | LDL | 0.485 | 0.012 | 0.00E+00 | 139,198 |  | 0.050 | 0.039 | 0.196 | 80,610 | 1.18E-02 | 1663.1 |
| rs75687619 | *TOMM40* | 19 | 45,399,344 | T/G | LDL | 0.174 | 0.016 | 8.05E-24 | 82,004 |  | 0.122 | 0.046 | 0.007 | 80,610 | 1.41E-03 | 116.1 |
| rs1800961 | *HNF4A* | 20 | 43,042,364 | C/T | LDL | 0.069 | 0.011 | 6.03E-10 | 142,698 |  | -0.038 | 0.041 | 0.347 | 80,610 | 2.93E-04 | 41.8 |
| rs2328223 | *RP5-905G11.3* | 20 | 17,845,921 | C/A | LDL | 0.030 | 0.005 | 5.63E-09 | 170,762 |  | -0.012 | 0.018 | 0.486 | 80,610 | 2.09E-04 | 35.8 |
| rs364585 | *RP11-157E14.1* | 20 | 12,962,718 | G/A | LDL | 0.025 | 0.004 | 4.28E-10 | 171,526 |  | 0.017 | 0.014 | 0.219 | 80,610 | 2.50E-04 | 42.9 |
| rs6016373 | *SNORD112* | 20 | 39,154,095 | A/G | LDL | 0.035 | 0.004 | 7.95E-19 | 171,559 |  | -0.003 | 0.014 | 0.829 | 80,610 | 5.18E-04 | 89.0 |
| rs6065311 | *TOP1* | 20 | 39,724,338 | C/T | LDL | 0.042 | 0.004 | 1.66E-30 | 171,333 |  | 0.027 | 0.014 | 0.048 | 80,610 | 7.83E-04 | 134.2 |
| rs4253776 | *PPARA* | 22 | 46,629,479 | G/A | LDL | 0.031 | 0.006 | 3.35E-08 | 171,071 |  | -0.021 | 0.021 | 0.331 | 80,610 | 1.62E-04 | 27.8 |
| rs5763662 | *MTMR3* | 22 | 30,378,703 | T/C | LDL | 0.077 | 0.012 | 1.19E-08 | 162,777 |  | 0.054 | 0.046 | 0.248 | 80,610 | 2.47E-04 | 40.2 |
| rs11591147 | *PCSK9* | 1 | 55,505,647 | G/T | TC | 0.334 | 0.017 | 8.83E-86 | 85,729 |  | -0.075 | 0.057 | 0.188 | 80,610 | 4.33E-03 | 373.0 |
| rs11802413 | *TMEM57* | 1 | 25,760,920 | T/C | TC | 0.029 | 0.004 | 1.58E-14 | 187,138 |  | 0.014 | 0.014 | 0.290 | 80,610 | 3.59E-04 | 67.2 |
| rs2642438 | *MARC1* | 1 | 220,970,028 | G/A | TC | 0.037 | 0.004 | 1.28E-18 | 179,599 |  | 0.001 | 0.015 | 0.957 | 80,610 | 4.76E-04 | 85.6 |
| rs2902875 | *RP11-101C11.1* | 1 | 55,695,535 | T/C | TC | 0.071 | 0.012 | 1.79E-08 | 94,590 |  | 0.037 | 0.035 | 0.293 | 80,610 | 3.49E-04 | 33.0 |
| rs558971 | *RP4-781K5.7* | 1 | 234,853,406 | G/A | TC | 0.040 | 0.004 | 7.03E-28 | 187,254 |  | 0.007 | 0.014 | 0.618 | 80,610 | 6.52E-04 | 122.2 |
| rs646776 | *PSRC1* | 1 | 109,818,530 | T/C | TC | 0.127 | 0.004 | 4.77E-187 | 187,288 |  | 0.017 | 0.017 | 0.318 | 80,610 | 4.87E-03 | 917.2 |
| rs6603981 | *EVI5* | 1 | 92,993,807 | T/C | TC | 0.035 | 0.004 | 7.85E-15 | 187,329 |  | -0.005 | 0.017 | 0.787 | 80,610 | 3.56E-04 | 66.6 |
| rs7534572 | *DOCK7* | 1 | 62,999,675 | G/C | TC | 0.063 | 0.006 | 3.60E-28 | 83,151 |  | 0.008 | 0.015 | 0.600 | 80,610 | 1.57E-03 | 130.8 |
| rs7551981 | *RP11-101C11.1* | 1 | 55,719,166 | T/G | TC | 0.036 | 0.004 | 7.50E-22 | 187,282 |  | 0.025 | 0.014 | 0.071 | 80,610 | 5.00E-04 | 93.6 |
| rs11563251 | *UGT1A1* | 2 | 234,679,384 | T/C | TC | 0.037 | 0.006 | 1.27E-09 | 187,107 |  | -0.031 | 0.023 | 0.174 | 80,610 | 2.08E-04 | 38.9 |
| rs11694172 | *FAM117B* | 2 | 203,532,304 | G/A | TC | 0.028 | 0.004 | 1.95E-09 | 187,092 |  | 0.018 | 0.016 | 0.248 | 80,610 | 2.44E-04 | 45.6 |
| rs17526895 | *INSIG2* | 2 | 118,815,958 | A/G | TC | 0.042 | 0.007 | 5.78E-09 | 184,199 |  | 0.026 | 0.026 | 0.325 | 80,610 | 2.13E-04 | 39.3 |
| rs2030746 | *AC073257.1* | 2 | 121,309,488 | T/C | TC | 0.020 | 0.004 | 3.60E-08 | 187,289 |  | -0.005 | 0.014 | 0.696 | 80,610 | 1.54E-04 | 28.9 |
| rs2287623 | *ABCB11* | 2 | 169,830,155 | G/A | TC | 0.027 | 0.004 | 4.09E-12 | 184,257 |  | -0.028 | 0.014 | 0.047 | 80,610 | 3.12E-04 | 57.5 |
| rs4988235 | *MCM6* | 2 | 136,608,646 | G/A | TC | 0.031 | 0.004 | 3.98E-14 | 183,761 |  | 0.028 | 0.019 | 0.135 | 80,610 | 3.23E-04 | 59.3 |
| rs515135 | *APOB* | 2 | 21,286,057 | C/T | TC | 0.124 | 0.005 | 6.38E-151 | 187,291 |  | 0.036 | 0.018 | 0.043 | 80,610 | 3.85E-03 | 724.3 |
| rs6544713 | *ABCG8* | 2 | 44,073,881 | T/C | TC | 0.077 | 0.004 | 1.69E-81 | 187,199 |  | 0.010 | 0.015 | 0.527 | 80,610 | 1.99E-03 | 373.5 |
| rs6709904 | *ABCG8* | 2 | 44,080,324 | A/G | TC | 0.055 | 0.008 | 8.40E-10 | 94,558 |  | -0.024 | 0.021 | 0.261 | 80,610 | 4.56E-04 | 43.1 |
| rs780093 | *GCKR* | 2 | 27,742,603 | T/C | TC | 0.052 | 0.004 | 2.59E-42 | 186,446 |  | 0.010 | 0.014 | 0.456 | 80,610 | 1.10E-03 | 204.6 |
| rs9306897 | *AC012361.1* | 2 | 21,138,066 | T/C | TC | 0.049 | 0.004 | 7.52E-37 | 185,808 |  | 0.003 | 0.015 | 0.837 | 80,610 | 9.35E-04 | 174.0 |
| rs13315871 | *PXK* | 3 | 58,381,287 | G/A | TC | 0.036 | 0.006 | 3.48E-08 | 187,287 |  | 0.023 | 0.024 | 0.347 | 80,610 | 1.81E-04 | 33.9 |
| rs7616006 | *GSTM5P1* | 3 | 12,267,648 | A/G | TC | 0.032 | 0.004 | 8.41E-17 | 187,246 |  | 0.017 | 0.014 | 0.227 | 80,610 | 4.09E-04 | 76.6 |
| rs7640978 | *CMTM6* | 3 | 32,533,010 | C/T | TC | 0.038 | 0.007 | 1.66E-08 | 186,485 |  | 0.011 | 0.024 | 0.645 | 80,610 | 1.74E-04 | 32.5 |
| rs6818397 | *RGS12* | 4 | 3,434,885 | T/G | TC | 0.025 | 0.004 | 9.51E-11 | 186,903 |  | 0.004 | 0.014 | 0.762 | 80,610 | 2.27E-04 | 42.4 |
| rs12916 | *HMGCR* | 5 | 74,656,539 | C/T | TC | 0.068 | 0.004 | 4.55E-74 | 182,530 |  | -0.006 | 0.014 | 0.668 | 80,610 | 1.97E-03 | 361.0 |
| rs4530754 | *CSNK1G3* | 5 | 122,855,416 | A/G | TC | 0.023 | 0.004 | 1.68E-09 | 187,272 |  | 0.005 | 0.014 | 0.702 | 80,610 | 2.27E-04 | 42.4 |
| rs6882076 | *TIMD4* | 5 | 156,390,297 | C/T | TC | 0.051 | 0.004 | 5.35E-41 | 187,270 |  | 0.011 | 0.014 | 0.434 | 80,610 | 1.01E-03 | 188.5 |
| rs11153594 | *FRK* | 6 | 116,354,591 | C/T | TC | 0.029 | 0.004 | 1.27E-14 | 187,230 |  | 0.001 | 0.014 | 0.969 | 80,610 | 3.46E-04 | 64.9 |
| rs112201728 | *SLC22A1* | 6 | 160,551,486 | T/C | TC | 0.058 | 0.010 | 1.20E-08 | 92,668 |  | 0.020 | 0.027 | 0.450 | 80,610 | 3.72E-04 | 34.4 |
| rs11753995 | *SLC22A1* | 6 | 160,575,366 | A/G | TC | 0.049 | 0.005 | 1.84E-23 | 187,264 |  | 0.012 | 0.019 | 0.526 | 80,610 | 5.54E-04 | 103.8 |
| rs1800562 | *HFE* | 6 | 26,093,141 | G/A | TC | 0.057 | 0.008 | 1.91E-12 | 185,469 |  | 0.023 | 0.030 | 0.449 | 80,610 | 2.90E-04 | 53.8 |
| rs2315065 | *RP1-81D8.3* | 6 | 161,108,144 | A/C | TC | 0.110 | 0.016 | 1.10E-11 | 68,430 |  | 0.043 | 0.027 | 0.114 | 80,610 | 7.10E-04 | 48.6 |
| rs2814982 | *RP3-391O22.3* | 6 | 34,546,560 | C/T | TC | 0.044 | 0.006 | 3.68E-15 | 187,263 |  | -0.030 | 0.022 | 0.161 | 80,610 | 3.20E-04 | 59.9 |
| rs3757354 | *MYLIP* | 6 | 16,127,407 | C/T | TC | 0.035 | 0.004 | 2.22E-15 | 187,247 |  | -0.010 | 0.017 | 0.570 | 80,610 | 3.67E-04 | 68.7 |
| rs9272775 | *HLA-DQA1* | 6 | 32,610,257 | C/T | TC | 0.032 | 0.006 | 2.13E-08 | 89,534 |  | -0.021 | 0.020 | 0.299 | 80,610 | 3.71E-04 | 33.2 |
| rs9376090 | *HBS1L* | 6 | 135,411,228 | T/C | TC | 0.025 | 0.004 | 2.60E-09 | 187,263 |  | -0.011 | 0.016 | 0.502 | 80,610 | 2.15E-04 | 40.3 |
| rs9391858 | *C6orf10* | 6 | 32,341,398 | G/A | TC | 0.050 | 0.005 | 7.20E-22 | 176,743 |  | 0.016 | 0.019 | 0.408 | 80,610 | 5.54E-04 | 98.0 |
| rs12670798 | *DNAH11* | 7 | 21,607,352 | C/T | TC | 0.036 | 0.004 | 9.48E-17 | 187,287 |  | -0.005 | 0.016 | 0.746 | 80,610 | 4.21E-04 | 78.8 |
| rs1997243 | *C7orf50* | 7 | 1,083,777 | G/A | TC | 0.033 | 0.005 | 2.72E-10 | 183,314 |  | 0.006 | 0.019 | 0.759 | 80,610 | 2.40E-04 | 44.1 |
| rs2073547 | *NPC1L1* | 7 | 44,582,331 | G/A | TC | 0.046 | 0.005 | 3.83E-21 | 184,098 |  | 0.006 | 0.018 | 0.728 | 80,610 | 5.11E-04 | 94.1 |
| rs10088180 | *AACP* | 8 | 18,228,116 | A/G | TC | 0.023 | 0.004 | 6.02E-10 | 187,142 |  | 0.002 | 0.016 | 0.910 | 80,610 | 1.74E-04 | 32.5 |
| rs2737252 | *TRPS1* | 8 | 116,663,898 | G/A | TC | 0.033 | 0.004 | 1.63E-16 | 187,202 |  | 0.025 | 0.016 | 0.114 | 80,610 | 3.85E-04 | 72.0 |
| rs2954029 | *RP11-136O12.2* | 8 | 126,490,972 | A/T | TC | 0.062 | 0.004 | 2.42E-65 | 187,216 |  | 0.002 | 0.014 | 0.892 | 80,610 | 1.68E-03 | 315.8 |
| rs4738684 | *CYP7A1* | 8 | 59,393,273 | A/G | TC | 0.039 | 0.004 | 1.12E-23 | 187,285 |  | -0.005 | 0.014 | 0.728 | 80,610 | 5.99E-04 | 112.2 |
| rs7832643 | *PLEC* | 8 | 145,022,657 | T/G | TC | 0.029 | 0.004 | 3.12E-13 | 178,980 |  | 0.011 | 0.014 | 0.456 | 80,610 | 3.41E-04 | 61.0 |
| rs9987289 | *RP11-115J16.1* | 8 | 9,183,358 | G/A | TC | 0.084 | 0.006 | 1.84E-36 | 173,502 |  | -0.023 | 0.025 | 0.358 | 80,610 | 1.03E-03 | 178.6 |
| rs11789603 | *ABCA1* | 9 | 107,647,019 | T/C | TC | 0.043 | 0.006 | 1.44E-11 | 186,565 |  | -0.003 | 0.022 | 0.899 | 80,610 | 2.54E-04 | 47.4 |
| rs1883025 | *ABCA1* | 9 | 107,664,301 | C/T | TC | 0.067 | 0.004 | 5.75E-53 | 186,557 |  | -0.013 | 0.016 | 0.408 | 80,610 | 1.37E-03 | 255.2 |
| rs2066714 | *ABCA1* | 9 | 107,586,753 | C/T | TC | 0.044 | 0.008 | 1.14E-08 | 93,811 |  | -0.001 | 0.020 | 0.957 | 80,610 | 3.60E-04 | 33.8 |
| rs3780181 | *VLDLR* | 9 | 2,640,759 | A/G | TC | 0.044 | 0.007 | 6.67E-10 | 186,134 |  | 0.083 | 0.029 | 0.005 | 80,610 | 2.08E-04 | 38.8 |
| rs579459 | *ABO* | 9 | 136,154,168 | C/T | TC | 0.062 | 0.004 | 8.83E-42 | 186,925 |  | 0.033 | 0.017 | 0.045 | 80,610 | 1.06E-03 | 198.6 |
| rs581080 | *TTC39B* | 9 | 15,305,378 | C/G | TC | 0.038 | 0.005 | 1.02E-13 | 187,121 |  | 0.034 | 0.018 | 0.058 | 80,610 | 3.44E-04 | 64.3 |
| rs10900221 | *MARCH8* | 10 | 45,988,597 | A/G | TC | 0.026 | 0.004 | 7.96E-09 | 186,785 |  | 0.000 | 0.016 | 0.988 | 80,610 | 2.07E-04 | 38.7 |
| rs10904908 | *RP11-124N14.4* | 10 | 17,260,290 | G/A | TC | 0.025 | 0.004 | 2.60E-11 | 187,112 |  | -0.016 | 0.015 | 0.289 | 80,610 | 2.58E-04 | 48.2 |
| rs12412743 | *TECTB* | 10 | 114,045,333 | C/T | TC | 0.030 | 0.005 | 6.98E-10 | 187,282 |  | 0.021 | 0.018 | 0.242 | 80,610 | 2.15E-04 | 40.2 |
| rs2255141 | *GPAM* | 10 | 113,933,886 | A/G | TC | 0.031 | 0.004 | 6.51E-16 | 187,266 |  | 0.029 | 0.015 | 0.053 | 80,610 | 3.46E-04 | 64.8 |
| rs10832962 | *SPTY2D1* | 11 | 18,656,271 | T/C | TC | 0.032 | 0.004 | 1.54E-14 | 187,161 |  | -0.001 | 0.016 | 0.944 | 80,610 | 3.48E-04 | 65.2 |
| rs11220462 | *ST3GAL4* | 11 | 126,243,952 | A/G | TC | 0.047 | 0.006 | 5.49E-15 | 156,953 |  | -0.046 | 0.020 | 0.024 | 80,610 | 4.25E-04 | 66.8 |
| rs1535 | *FADS2* | 11 | 61,597,972 | A/G | TC | 0.050 | 0.004 | 8.62E-39 | 182,527 |  | 0.000 | 0.015 | 0.980 | 80,610 | 9.88E-04 | 180.4 |
| rs4752805 | *PTPRJ* | 11 | 48,018,355 | G/A | TC | 0.025 | 0.004 | 1.62E-09 | 187,233 |  | 0.031 | 0.016 | 0.046 | 80,610 | 2.00E-04 | 37.5 |
| rs964184 | *ZNF259* | 11 | 116,648,917 | G/C | TC | 0.121 | 0.008 | 2.84E-55 | 94,573 |  | 0.037 | 0.020 | 0.064 | 80,610 | 2.69E-03 | 255.2 |
| rs10773003 | *SBNO1* | 12 | 123,775,127 | A/G | TC | 0.037 | 0.006 | 4.08E-09 | 187,101 |  | 0.007 | 0.024 | 0.759 | 80,610 | 2.16E-04 | 40.5 |
| rs2244608 | *HNF1A-AS1* | 12 | 121,416,988 | G/A | TC | 0.031 | 0.004 | 9.62E-18 | 187,223 |  | 0.008 | 0.015 | 0.594 | 80,610 | 3.82E-04 | 71.6 |
| rs3184504 | *SH2B3* | 12 | 111,884,608 | C/T | TC | 0.032 | 0.004 | 1.62E-17 | 177,714 |  | 0.046 | 0.014 | 0.001 | 80,610 | 4.15E-04 | 73.9 |
| rs6573778 | *NYNRIN* | 14 | 24,872,209 | T/C | TC | 0.026 | 0.004 | 2.96E-11 | 187,079 |  | -0.013 | 0.014 | 0.360 | 80,610 | 2.43E-04 | 45.5 |
| rs10468017 | *ALDH1A2* | 15 | 58,678,512 | T/C | TC | 0.062 | 0.004 | 7.23E-48 | 181,378 |  | -0.012 | 0.015 | 0.421 | 80,610 | 1.31E-03 | 237.9 |
| rs633695 | *LIPC* | 15 | 58,725,839 | G/A | TC | 0.043 | 0.006 | 1.05E-14 | 93,067 |  | -0.002 | 0.015 | 0.916 | 80,610 | 5.98E-04 | 55.7 |
| rs2000999 | *TXNL4B* | 16 | 72,108,093 | A/G | TC | 0.062 | 0.004 | 6.80E-41 | 185,692 |  | 0.014 | 0.017 | 0.427 | 80,610 | 1.06E-03 | 196.6 |
| rs247616 | *AC012181.1* | 16 | 56,989,590 | T/C | TC | 0.050 | 0.004 | 4.47E-32 | 185,621 |  | -0.015 | 0.015 | 0.310 | 80,610 | 8.38E-04 | 155.6 |
| rs2886232 | *ABCA10* | 17 | 67,150,176 | T/C | TC | 0.036 | 0.006 | 3.87E-08 | 176,571 |  | 0.024 | 0.023 | 0.297 | 80,610 | 1.89E-04 | 33.3 |
| rs314253 | *ASGR1* | 17 | 7,091,650 | T/C | TC | 0.023 | 0.004 | 2.81E-10 | 183,868 |  | -0.020 | 0.014 | 0.173 | 80,610 | 2.16E-04 | 39.7 |
| rs6504872 | *C17orf57* | 17 | 45,438,952 | T/C | TC | 0.025 | 0.004 | 6.99E-12 | 185,712 |  | -0.038 | 0.014 | 0.006 | 80,610 | 2.75E-04 | 51.0 |
| rs2156552 | *LIPG* | 18 | 47,181,668 | T/A | TC | 0.057 | 0.005 | 1.25E-31 | 183,439 |  | -0.030 | 0.019 | 0.109 | 80,610 | 8.01E-04 | 147.1 |
| rs2228603 | *NCAN* | 19 | 19,329,924 | C/T | TC | 0.122 | 0.007 | 1.05E-62 | 170,510 |  | 0.021 | 0.029 | 0.470 | 80,610 | 1.82E-03 | 311.1 |
| rs281393 | *RASIP1* | 19 | 49,224,484 | C/T | TC | 0.032 | 0.006 | 4.26E-08 | 93,067 |  | -0.019 | 0.014 | 0.191 | 80,610 | 3.68E-04 | 34.3 |
| rs6511720 | *LDLR* | 19 | 11,202,306 | G/T | TC | 0.185 | 0.006 | 5.43E-202 | 184,764 |  | 0.011 | 0.021 | 0.608 | 80,610 | 5.30E-03 | 984.2 |
| rs7412 | *APOE* | 19 | 45,412,079 | C/T | TC | 0.374 | 0.010 | 1.56E-283 | 92,046 |  | 0.026 | 0.026 | 0.329 | 80,610 | 1.62E-02 | 1514.5 |
| rs75687619 | *TOMM40* | 19 | 45,399,344 | T/G | TC | 0.159 | 0.015 | 3.61E-22 | 91,544 |  | 0.122 | 0.046 | 0.007 | 80,610 | 1.18E-03 | 108.3 |
| rs8103315 | *BCL3* | 19 | 45,254,168 | A/C | TC | 0.042 | 0.006 | 5.94E-15 | 156,370 |  | -0.014 | 0.023 | 0.543 | 80,610 | 3.76E-04 | 58.9 |
| rs1800961 | *HNF4A* | 20 | 43,042,364 | C/T | TC | 0.106 | 0.010 | 1.34E-24 | 156,406 |  | -0.038 | 0.041 | 0.347 | 80,610 | 7.06E-04 | 110.6 |
| rs2235367 | *ZHX3* | 20 | 39,830,122 | G/A | TC | 0.036 | 0.004 | 7.22E-25 | 185,691 |  | 0.029 | 0.014 | 0.035 | 80,610 | 5.60E-04 | 104.0 |
| rs2277862 | *FER1L4* | 20 | 34,152,782 | C/T | TC | 0.035 | 0.005 | 5.26E-11 | 185,738 |  | 0.007 | 0.019 | 0.725 | 80,610 | 2.42E-04 | 45.0 |
| rs6016373 | *SNORD112* | 20 | 39,154,095 | A/G | TC | 0.032 | 0.004 | 1.00E-17 | 185,730 |  | -0.003 | 0.014 | 0.829 | 80,610 | 4.23E-04 | 78.5 |
| rs138777 | *TOM1* | 22 | 35,711,098 | A/G | TC | 0.021 | 0.004 | 4.74E-08 | 185,274 |  | 0.022 | 0.014 | 0.126 | 80,610 | 1.81E-04 | 33.5 |
| rs181360 | *UBE2L3* | 22 | 21,928,916 | T/G | TC | 0.028 | 0.004 | 7.32E-10 | 178,322 |  | 0.032 | 0.018 | 0.071 | 80,610 | 2.34E-04 | 41.8 |
| rs4253772 | *PPARA* | 22 | 46,627,603 | T/C | TC | 0.032 | 0.006 | 9.85E-09 | 185,188 |  | -0.020 | 0.022 | 0.354 | 80,610 | 1.66E-04 | 30.8 |
| rs12748152 | *AL034380.1* | 1 | 27,138,393 | T/C | TG | 0.037 | 0.006 | 1.10E-09 | 177,762 |  | -0.017 | 0.026 | 0.509 | 80,610 | 2.24E-04 | 39.8 |
| rs1321257 | *GALNT2* | 1 | 230,305,312 | G/A | TG | 0.040 | 0.003 | 5.99E-31 | 177,758 |  | 0.016 | 0.014 | 0.250 | 80,610 | 7.86E-04 | 139.8 |
| rs17513135 | *RP11-15J6.1* | 1 | 40,035,686 | T/C | TG | 0.022 | 0.004 | 1.63E-08 | 174,742 |  | 0.017 | 0.016 | 0.310 | 80,610 | 1.82E-04 | 31.8 |
| rs4587594 | *DOCK7* | 1 | 63,133,930 | G/A | TG | 0.069 | 0.004 | 3.50E-82 | 177,772 |  | 0.005 | 0.015 | 0.749 | 80,610 | 2.21E-03 | 393.2 |
| rs1260326 | *GCKR* | 2 | 27,730,940 | T/C | TG | 0.115 | 0.003 | 2.29E-239 | 177,765 |  | 0.009 | 0.014 | 0.535 | 80,610 | 6.37E-03 | 1140.0 |
| rs13389219 | *COBLL1* | 2 | 165,528,876 | C/T | TG | 0.027 | 0.003 | 2.60E-15 | 177,783 |  | -0.008 | 0.014 | 0.571 | 80,610 | 3.57E-04 | 63.5 |
| rs2972146 | *AC068138.1* | 2 | 227,100,698 | T/G | TG | 0.028 | 0.003 | 2.97E-15 | 174,704 |  | -0.028 | 0.014 | 0.046 | 80,610 | 3.91E-04 | 68.3 |
| rs676210 | *APOB* | 2 | 21,231,524 | G/A | TG | 0.073 | 0.004 | 3.28E-71 | 177,782 |  | 0.038 | 0.016 | 0.020 | 80,610 | 1.98E-03 | 353.2 |
| rs10440120 | *U6* | 3 | 12,486,964 | C/A | TG | 0.031 | 0.004 | 5.34E-11 | 174,886 |  | -0.022 | 0.018 | 0.226 | 80,610 | 2.76E-04 | 48.4 |
| rs645040 | *RP11-463H24.1* | 3 | 135,926,622 | T/G | TG | 0.029 | 0.004 | 1.83E-12 | 177,779 |  | -0.009 | 0.016 | 0.585 | 80,610 | 3.02E-04 | 53.7 |
| rs442177 | *AFF1* | 4 | 88,030,261 | T/G | TG | 0.031 | 0.003 | 1.32E-18 | 177,798 |  | 0.010 | 0.014 | 0.480 | 80,610 | 4.93E-04 | 87.7 |
| rs6831256 | *DOK7* | 4 | 3,473,139 | G/A | TG | 0.026 | 0.004 | 1.60E-12 | 177,495 |  | 0.002 | 0.014 | 0.868 | 80,610 | 3.06E-04 | 54.3 |
| rs6882076 | *TIMD4* | 5 | 156,390,297 | C/T | TG | 0.029 | 0.004 | 1.51E-15 | 177,778 |  | 0.011 | 0.014 | 0.434 | 80,610 | 3.75E-04 | 66.8 |
| rs9686661 | *AC022431.2* | 5 | 55,861,786 | T/C | TG | 0.038 | 0.004 | 2.54E-16 | 177,050 |  | 0.008 | 0.018 | 0.663 | 80,610 | 4.19E-04 | 74.2 |
| rs2239520 | *PSORS1C1* | 6 | 31,088,922 | G/A | TG | 0.024 | 0.004 | 4.14E-10 | 151,047 |  | -0.021 | 0.014 | 0.119 | 80,610 | 2.69E-04 | 40.7 |
| rs2247056 | *XXbac-BPG248L24.13* | 6 | 31,265,490 | C/T | TG | 0.038 | 0.004 | 3.86E-21 | 174,062 |  | -0.001 | 0.016 | 0.975 | 80,610 | 5.39E-04 | 93.9 |
| rs2665357 | *SLC22A3* | 6 | 160,848,167 | C/A | TG | 0.021 | 0.003 | 8.33E-10 | 172,850 |  | 0.013 | 0.014 | 0.336 | 80,610 | 2.39E-04 | 41.3 |
| rs634869 | *RP11-12A2.3* | 6 | 139,831,757 | T/C | TG | 0.027 | 0.003 | 1.78E-14 | 177,755 |  | -0.013 | 0.014 | 0.342 | 80,610 | 3.82E-04 | 67.9 |
| rs719726 | *RP11-73O6.4* | 6 | 127,414,801 | T/C | TG | 0.020 | 0.004 | 2.49E-08 | 168,319 |  | -0.016 | 0.014 | 0.253 | 80,610 | 1.92E-04 | 32.3 |
| rs998584 | *VEGFA* | 6 | 43,757,896 | A/C | TG | 0.029 | 0.004 | 3.42E-15 | 174,573 |  | -0.002 | 0.015 | 0.911 | 80,610 | 3.59E-04 | 62.7 |
| rs11974409 | *TBL2* | 7 | 72,989,390 | A/G | TG | 0.090 | 0.004 | 1.36E-100 | 177,786 |  | 0.005 | 0.018 | 0.799 | 80,610 | 2.57E-03 | 458.2 |
| rs287621 | *KLF14* | 7 | 130,435,181 | T/C | TG | 0.022 | 0.004 | 7.67E-09 | 177,813 |  | 0.011 | 0.015 | 0.461 | 80,610 | 2.02E-04 | 36.0 |
| rs38855 | *MET* | 7 | 116,358,044 | A/G | TG | 0.019 | 0.003 | 2.11E-08 | 177,825 |  | -0.018 | 0.014 | 0.188 | 80,610 | 1.81E-04 | 32.1 |
| rs4719841 | *MIR148A* | 7 | 25,997,536 | G/A | TG | 0.023 | 0.003 | 8.86E-11 | 177,775 |  | -0.009 | 0.014 | 0.533 | 80,610 | 2.62E-04 | 46.6 |
| rs12676857 | *NAT2* | 8 | 18,266,572 | C/T | TG | 0.033 | 0.005 | 7.29E-12 | 177,732 |  | 0.005 | 0.019 | 0.784 | 80,610 | 2.93E-04 | 52.1 |
| rs12678919 | *LPL* | 8 | 19,844,222 | A/G | TG | 0.170 | 0.006 | 1.82E-199 | 177,750 |  | -0.011 | 0.022 | 0.627 | 80,610 | 5.17E-03 | 923.7 |
| rs2954022 | *RP11-136O12.2* | 8 | 126,482,621 | C/A | TG | 0.078 | 0.003 | 2.23E-113 | 177,750 |  | 0.003 | 0.014 | 0.844 | 80,610 | 3.13E-03 | 558.7 |
| rs4738684 | *CYP7A1* | 8 | 59,393,273 | A/G | TG | 0.021 | 0.004 | 8.82E-09 | 177,790 |  | -0.005 | 0.014 | 0.728 | 80,610 | 1.93E-04 | 34.3 |
| rs6995541 | *PINX1* | 8 | 10,671,260 | G/A | TG | 0.027 | 0.004 | 1.34E-12 | 177,486 |  | -0.008 | 0.015 | 0.604 | 80,610 | 2.89E-04 | 51.3 |
| rs10761762 | *JMJD1C* | 10 | 65,184,717 | T/C | TG | 0.027 | 0.003 | 1.06E-17 | 177,823 |  | 0.004 | 0.014 | 0.779 | 80,610 | 3.76E-04 | 66.9 |
| rs1832007 | *AKR1C4* | 10 | 5,254,847 | A/G | TG | 0.033 | 0.005 | 1.72E-12 | 177,504 |  | -0.018 | 0.019 | 0.365 | 80,610 | 2.73E-04 | 48.4 |
| rs2068888 | *CYP26A1* | 10 | 94,839,642 | G/A | TG | 0.024 | 0.003 | 1.68E-11 | 177,712 |  | -0.030 | 0.014 | 0.027 | 80,610 | 2.83E-04 | 50.2 |
| rs2250802 | *GPAM* | 10 | 113,921,354 | A/G | TG | 0.023 | 0.004 | 1.21E-10 | 174,734 |  | -0.029 | 0.015 | 0.050 | 80,610 | 2.21E-04 | 38.6 |
| rs10501321 | *MADD* | 11 | 47,294,626 | T/C | TG | 0.022 | 0.004 | 1.41E-08 | 177,680 |  | -0.025 | 0.015 | 0.091 | 80,610 | 2.14E-04 | 38.1 |
| rs10790162 | *BUD13* | 11 | 116,639,104 | A/G | TG | 0.231 | 0.007 | 1.10E-249 | 177,771 |  | 0.057 | 0.027 | 0.031 | 80,610 | 7.02E-03 | 1257.5 |
| rs11820504 | *AP000770.1* | 11 | 116,529,442 | C/T | TG | 0.060 | 0.004 | 1.10E-39 | 172,813 |  | -0.004 | 0.018 | 0.833 | 80,610 | 1.09E-03 | 188.4 |
| rs174535 | *RP11-467L20.9* | 11 | 61,551,356 | C/T | TG | 0.047 | 0.003 | 1.73E-41 | 177,773 |  | 0.000 | 0.014 | 0.981 | 80,610 | 1.07E-03 | 191.1 |
| rs11057408 | *ZNF664* | 12 | 124,464,836 | G/T | TG | 0.026 | 0.004 | 2.05E-12 | 174,454 |  | -0.015 | 0.015 | 0.317 | 80,610 | 3.11E-04 | 54.3 |
| rs16948098 | *FRMD5* | 15 | 44,219,607 | A/G | TG | 0.080 | 0.009 | 4.84E-17 | 163,328 |  | -0.017 | 0.032 | 0.589 | 80,610 | 4.94E-04 | 80.8 |
| rs2043085 | *ALDH1A2* | 15 | 58,680,954 | T/C | TG | 0.033 | 0.003 | 7.81E-20 | 176,147 |  | -0.011 | 0.014 | 0.452 | 80,610 | 5.25E-04 | 92.5 |
| rs588136 | *RP11-355N15.1* | 15 | 58,730,498 | C/T | TG | 0.050 | 0.004 | 3.37E-30 | 176,173 |  | 0.004 | 0.017 | 0.837 | 80,610 | 8.27E-04 | 145.8 |
| rs1800775 | *CETP* | 16 | 56,995,236 | C/A | TG | 0.040 | 0.004 | 1.33E-26 | 172,715 |  | -0.011 | 0.014 | 0.402 | 80,610 | 7.41E-04 | 128.0 |
| rs3198697 | *PDXDC1* | 16 | 15,129,940 | C/T | TG | 0.020 | 0.003 | 2.21E-08 | 175,934 |  | 0.008 | 0.014 | 0.554 | 80,610 | 1.93E-04 | 33.9 |
| rs749671 | *ZNF646* | 16 | 31,088,347 | G/A | TG | 0.021 | 0.003 | 6.11E-10 | 176,205 |  | 0.002 | 0.014 | 0.907 | 80,610 | 2.19E-04 | 38.5 |
| rs8077889 | *MPP3* | 17 | 41,878,166 | C/A | TG | 0.025 | 0.004 | 9.88E-09 | 176,194 |  | -0.006 | 0.017 | 0.701 | 80,610 | 2.04E-04 | 36.0 |
| rs10401969 | *SUGP1* | 19 | 19,407,718 | T/C | TG | 0.121 | 0.007 | 9.70E-70 | 176,173 |  | 0.030 | 0.027 | 0.269 | 80,610 | 1.96E-03 | 346.5 |
| rs3760627 | *APOC2* | 19 | 45,457,180 | C/T | TG | 0.019 | 0.003 | 5.29E-09 | 176,201 |  | 0.006 | 0.014 | 0.669 | 80,610 | 1.75E-04 | 30.9 |
| rs439401 | *APOE* | 19 | 45,414,451 | C/T | TG | 0.066 | 0.004 | 1.42E-66 | 152,584 |  | -0.004 | 0.014 | 0.778 | 80,610 | 1.97E-03 | 300.7 |
| rs7248104 | *INSR* | 19 | 7,224,431 | G/A | TG | 0.022 | 0.003 | 5.05E-10 | 176,083 |  | 0.025 | 0.014 | 0.064 | 80,610 | 2.42E-04 | 42.6 |
| rs731839 | *PEPD* | 19 | 33,899,065 | G/A | TG | 0.022 | 0.004 | 2.65E-09 | 176,161 |  | 0.024 | 0.015 | 0.117 | 80,610 | 2.20E-04 | 38.7 |
| rs4810479 | *PLTP* | 20 | 44,545,048 | C/T | TG | 0.047 | 0.004 | 2.07E-34 | 176,192 |  | 0.000 | 0.016 | 0.978 | 80,610 | 8.82E-04 | 155.6 |
| rs6029143 | *SNORD112* | 20 | 39,118,662 | C/T | TG | 0.039 | 0.007 | 4.93E-08 | 176,257 |  | 0.040 | 0.028 | 0.156 | 80,610 | 1.69E-04 | 29.9 |
| rs3761445 | *MAFF* | 22 | 38,595,411 | A/G | TG | 0.023 | 0.003 | 8.06E-12 | 175,846 |  | 0.018 | 0.014 | 0.205 | 80,610 | 2.65E-04 | 46.6 |

Supplementary Table S7. Summary information of instrumental variables for lipids and ALS in the European population. Note: SNP: the label of single-nucleotide polymorphism; CHR: chromosome; BP: base position; A1: effect allele; A2: alternative allele; BETA: SNP effect size; SE: standard error of the SNP effect size; PVE: proportion of variance explained by the SNP; *p*, *N*, and *F* represent *p* value, sample size, and *F* statistic, respectively. All the selected instruments collectively explain about 5.85% HDL variation, 8.75% LDL variation, 8.05% TC variation and 4.89% TG variation at the observed scale. For these instrumental variables, all the *F* statistics are above 10 (ranging from 29.9 to 1674.2 for HDL; ranging from 27.8 to 1663.1 for LDL; ranging from 28.9 to 1514.5 for TC and ranging from 29.9 to 1257.5 for TG) with an average *F* statistic of (121.0 for HDL; 162.3 for LDL; 140.6 for TC and 162.8 for TG) and an overall *F* statistic of (130.2 for HDL; 191.1 for LDL; 172.6 for TC and 169.9 for TG), suggesting that the selected genetic variants have sufficient strong effects for instrument and the bias of weak instrument is unlikely.

| SNP | GENE | CHR | BP | A1/A2 | lipids | | | | |  | FTD | | | | PVE | *F* |
| --- | --- | --- | --- | --- | --- | --- | --- | --- | --- | --- | --- | --- | --- | --- | --- | --- |
|  |  |  |  |  | Subtype | BETA | SE | *p* | *N* |  | BETA | SE | *p* | *N* |  |  |
| rs12133576 | *DR1* | 1 | 93,816,400 | A/G | HDL | 0.024 | 0.004 | 6.15E-11 | 187,123 |  | 0.011 | 0.048 | 0.824 | 12,928 | 2.58E-04 | 48.2 |
| rs12145743 | *RRNAD1* | 1 | 156,700,651 | G/T | HDL | 0.020 | 0.004 | 1.80E-08 | 181,336 |  | -0.063 | 0.041 | 0.128 | 12,928 | 1.75E-04 | 31.8 |
| rs12740374 | *CELSR2* | 1 | 109,817,590 | T/G | HDL | 0.034 | 0.004 | 1.69E-15 | 186,888 |  | 0.018 | 0.048 | 0.700 | 12,928 | 3.74E-04 | 70.0 |
| rs12748152 | *AL034380.1* | 1 | 27,138,393 | C/T | HDL | 0.051 | 0.006 | 9.74E-16 | 187,057 |  | 0.121 | 0.083 | 0.145 | 12,928 | 3.56E-04 | 66.6 |
| rs1689797 | *GS1-122H1.2* | 1 | 182,150,978 | C/A | HDL | 0.036 | 0.004 | 2.85E-21 | 187,126 |  | 0.033 | 0.044 | 0.455 | 12,928 | 5.28E-04 | 98.9 |
| rs2642438 | *MARC1* | 1 | 220,970,028 | G/A | HDL | 0.030 | 0.004 | 7.78E-14 | 179,439 |  | -0.038 | 0.053 | 0.474 | 12,928 | 3.36E-04 | 60.4 |
| rs4650994 | *C1orf220* | 1 | 178,515,312 | G/A | HDL | 0.021 | 0.003 | 6.70E-09 | 186,927 |  | 0.032 | 0.039 | 0.414 | 12,928 | 2.04E-04 | 38.1 |
| rs4660293 | *PABPC4* | 1 | 40,028,180 | A/G | HDL | 0.035 | 0.004 | 2.86E-18 | 187,027 |  | -0.025 | 0.048 | 0.608 | 12,928 | 4.16E-04 | 77.9 |
| rs4846914 | *GALNT2* | 1 | 230,295,691 | A/G | HDL | 0.048 | 0.003 | 3.51E-41 | 186,995 |  | 0.037 | 0.040 | 0.352 | 12,928 | 1.06E-03 | 198.5 |
| rs1047891 | *CPS1* | 2 | 211,540,507 | C/A | HDL | 0.027 | 0.004 | 8.73E-10 | 182,043 |  | -0.014 | 0.057 | 0.801 | 12,928 | 2.61E-04 | 47.6 |
| rs1515110 | *AC068138.1* | 2 | 227,122,216 | G/T | HDL | 0.032 | 0.004 | 8.04E-18 | 187,081 |  | 0.062 | 0.041 | 0.126 | 12,928 | 4.55E-04 | 85.2 |
| rs7607980 | *COBLL1* | 2 | 165,551,201 | C/T | HDL | 0.045 | 0.005 | 1.81E-15 | 187,036 |  | -0.100 | 0.059 | 0.090 | 12,928 | 3.95E-04 | 73.9 |
| rs13076253 | *CPNE4* | 3 | 131,751,775 | A/C | HDL | 0.028 | 0.005 | 4.96E-09 | 186,809 |  | 0.020 | 0.056 | 0.722 | 12,928 | 1.86E-04 | 34.8 |
| rs13099479 | *PBRM1* | 3 | 52,677,478 | A/G | HDL | 0.036 | 0.006 | 1.82E-08 | 187,132 |  | -0.037 | 0.072 | 0.610 | 12,928 | 1.80E-04 | 33.7 |
| rs2013208 | *RBM5* | 3 | 50,129,399 | T/C | HDL | 0.025 | 0.004 | 8.92E-12 | 169,708 |  | 0.007 | 0.038 | 0.861 | 12,928 | 2.93E-04 | 49.8 |
| rs2606736 | *ATG7* | 3 | 11,400,249 | C/T | HDL | 0.025 | 0.004 | 4.80E-08 | 129,328 |  | 0.065 | 0.044 | 0.139 | 12,928 | 2.53E-04 | 32.7 |
| rs6805251 | *GSK3B* | 3 | 119,560,606 | T/C | HDL | 0.020 | 0.004 | 1.33E-08 | 186,301 |  | 0.014 | 0.040 | 0.728 | 12,928 | 1.75E-04 | 32.7 |
| rs687339 | *RP11-463H24.1* | 3 | 135,932,359 | C/T | HDL | 0.032 | 0.004 | 7.11E-13 | 187,105 |  | -0.024 | 0.054 | 0.663 | 12,928 | 3.02E-04 | 56.6 |
| rs10019888 | *RP11-324H7.1* | 4 | 26,062,990 | A/G | HDL | 0.027 | 0.005 | 4.90E-08 | 187,077 |  | 0.108 | 0.051 | 0.036 | 12,928 | 1.84E-04 | 34.5 |
| rs13107325 | *SLC39A8* | 4 | 103,188,709 | C/T | HDL | 0.071 | 0.008 | 1.07E-15 | 179,316 |  | 0.022 | 0.075 | 0.774 | 12,928 | 4.59E-04 | 82.4 |
| rs2602836 | *RP11-696N14.1* | 4 | 100,014,805 | A/G | HDL | 0.019 | 0.003 | 4.96E-08 | 187,102 |  | -0.069 | 0.044 | 0.113 | 12,928 | 1.70E-04 | 31.9 |
| rs3822072 | *FAM13A* | 4 | 89,741,269 | G/A | HDL | 0.025 | 0.003 | 4.06E-12 | 187,115 |  | 0.001 | 0.042 | 0.989 | 12,928 | 2.91E-04 | 54.5 |
| rs6450176 | *ARL15* | 5 | 53,298,025 | G/A | HDL | 0.025 | 0.004 | 6.88E-10 | 187,132 |  | 0.083 | 0.048 | 0.086 | 12,928 | 2.27E-04 | 42.4 |
| rs1936800 | *RP11-73O6.4* | 6 | 127,436,064 | C/T | HDL | 0.020 | 0.003 | 3.06E-10 | 187,111 |  | 0.001 | 0.045 | 0.988 | 12,928 | 1.85E-04 | 34.6 |
| rs205262 | *C6orf106* | 6 | 34,563,164 | A/G | HDL | 0.028 | 0.004 | 3.88E-13 | 181,707 |  | 0.022 | 0.043 | 0.611 | 12,928 | 2.90E-04 | 52.7 |
| rs3861397 | *RP11-12A2.3* | 6 | 139,828,916 | A/G | HDL | 0.024 | 0.004 | 8.40E-11 | 187,084 |  | -0.003 | 0.042 | 0.938 | 12,928 | 2.38E-04 | 44.4 |
| rs9457931 | *LPAL2* | 6 | 160,929,904 | A/G | HDL | 0.055 | 0.007 | 7.30E-13 | 171,669 |  | 0.021 | 0.095 | 0.830 | 12,928 | 3.33E-04 | 57.2 |
| rs998584 | *VEGFA* | 6 | 43,757,896 | C/A | HDL | 0.026 | 0.004 | 2.27E-11 | 183,791 |  | 0.040 | 0.045 | 0.370 | 12,928 | 2.55E-04 | 46.8 |
| rs11765979 | *KLF14* | 7 | 130,445,877 | C/A | HDL | 0.041 | 0.005 | 3.11E-17 | 94,311 |  | -0.026 | 0.039 | 0.505 | 12,928 | 7.81E-04 | 73.7 |
| rs17173637 | *ABP1* | 7 | 150,529,449 | T/C | HDL | 0.036 | 0.006 | 1.90E-08 | 183,901 |  | 0.121 | 0.067 | 0.072 | 12,928 | 2.20E-04 | 40.6 |
| rs4142995 | *SNX13* | 7 | 17,919,258 | G/T | HDL | 0.026 | 0.004 | 9.37E-12 | 165,161 |  | -0.021 | 0.048 | 0.658 | 12,928 | 3.06E-04 | 50.5 |
| rs4917014 | *AC020743.4* | 7 | 50,305,863 | G/T | HDL | 0.022 | 0.004 | 1.03E-08 | 186,868 |  | -0.088 | 0.041 | 0.032 | 12,928 | 2.03E-04 | 38.0 |
| rs702485 | *DAGLB* | 7 | 6,449,272 | G/A | HDL | 0.024 | 0.003 | 6.45E-12 | 186,974 |  | 0.002 | 0.041 | 0.953 | 12,928 | 2.73E-04 | 51.1 |
| rs10087900 | *GPIHBP1* | 8 | 144,303,418 | G/A | HDL | 0.023 | 0.004 | 2.17E-09 | 183,672 |  | -0.030 | 0.040 | 0.452 | 12,928 | 2.24E-04 | 41.2 |
| rs10808546 | *RP11-136O12.2* | 8 | 126,495,818 | T/C | HDL | 0.041 | 0.003 | 4.11E-30 | 185,835 |  | -0.006 | 0.039 | 0.879 | 12,928 | 7.78E-04 | 144.7 |
| rs13702 | *LPL* | 8 | 19,824,492 | C/T | HDL | 0.106 | 0.004 | 1.28E-160 | 187,044 |  | -0.040 | 0.045 | 0.378 | 12,928 | 4.13E-03 | 775.2 |
| rs1866956 | *AC107964.1* | 8 | 19,748,921 | T/C | HDL | 0.022 | 0.004 | 7.96E-10 | 187,031 |  | 0.001 | 0.043 | 0.976 | 12,928 | 1.84E-04 | 34.4 |
| rs2293889 | *TRPS1* | 8 | 116,599,199 | G/T | HDL | 0.031 | 0.004 | 4.27E-17 | 180,102 |  | -0.022 | 0.041 | 0.598 | 12,928 | 4.41E-04 | 79.5 |
| rs4240624 | *RP11-115J16.1* | 8 | 9,184,231 | A/G | HDL | 0.082 | 0.006 | 1.32E-45 | 185,696 |  | 0.109 | 0.077 | 0.155 | 12,928 | 1.07E-03 | 198.9 |
| rs11789603 | *ABCA1* | 9 | 107,647,019 | T/C | HDL | 0.060 | 0.006 | 3.70E-21 | 184,432 |  | -0.083 | 0.078 | 0.292 | 12,928 | 5.42E-04 | 100.0 |
| rs1883025 | *ABCA1* | 9 | 107,664,301 | C/T | HDL | 0.070 | 0.004 | 1.50E-65 | 186,365 |  | -0.005 | 0.045 | 0.917 | 12,928 | 1.55E-03 | 289.8 |
| rs2066714 | *ABCA1* | 9 | 107,586,753 | C/T | HDL | 0.045 | 0.007 | 7.26E-10 | 93,528 |  | 0.069 | 0.075 | 0.359 | 12,928 | 4.35E-04 | 40.7 |
| rs686030 | *TTC39B* | 9 | 15,304,782 | A/C | HDL | 0.055 | 0.005 | 4.29E-27 | 187,035 |  | 0.106 | 0.056 | 0.058 | 12,928 | 6.73E-04 | 126.0 |
| rs10761771 | *RP11-351O1.3* | 10 | 65,230,164 | C/T | HDL | 0.020 | 0.003 | 4.12E-09 | 182,895 |  | 0.057 | 0.041 | 0.167 | 12,928 | 1.85E-04 | 33.9 |
| rs12412743 | *TECTB* | 10 | 114,045,333 | C/T | HDL | 0.029 | 0.005 | 1.31E-09 | 187,095 |  | 0.037 | 0.053 | 0.483 | 12,928 | 2.23E-04 | 41.8 |
| rs2250802 | *GPAM* | 10 | 113,921,354 | G/A | HDL | 0.034 | 0.004 | 2.02E-17 | 184,088 |  | 0.002 | 0.044 | 0.973 | 12,928 | 4.35E-04 | 80.1 |
| rs970548 | *MARCH8* | 10 | 46,013,277 | C/A | HDL | 0.026 | 0.004 | 1.71E-10 | 186,596 |  | -0.032 | 0.046 | 0.483 | 12,928 | 2.34E-04 | 43.8 |
| rs102275 | *C11orf10* | 11 | 61,557,803 | T/C | HDL | 0.039 | 0.004 | 6.40E-28 | 187,085 |  | -0.004 | 0.041 | 0.914 | 12,928 | 6.67E-04 | 124.8 |
| rs10501321 | *MADD* | 11 | 47,294,626 | C/T | HDL | 0.048 | 0.004 | 3.54E-38 | 186,984 |  | 0.013 | 0.043 | 0.758 | 12,928 | 9.62E-04 | 180.0 |
| rs12801636 | *PCNXL3* | 11 | 65,391,317 | A/G | HDL | 0.024 | 0.004 | 3.15E-08 | 187,099 |  | 0.009 | 0.047 | 0.854 | 12,928 | 1.67E-04 | 31.3 |
| rs499974 | *MOGAT2* | 11 | 75,455,021 | C/A | HDL | 0.026 | 0.004 | 1.12E-08 | 186,749 |  | -0.111 | 0.076 | 0.146 | 12,928 | 1.91E-04 | 35.7 |
| rs7112577 | *PAFAH1B2* | 11 | 117,044,603 | G/C | HDL | 0.083 | 0.013 | 2.34E-10 | 89,235 |  | -0.057 | 0.113 | 0.619 | 12,928 | 4.59E-04 | 41.0 |
| rs11045163 | *RP11-284H19.1* | 12 | 20,463,526 | G/A | HDL | 0.022 | 0.004 | 3.20E-09 | 187,075 |  | 0.041 | 0.042 | 0.332 | 12,928 | 2.05E-04 | 38.4 |
| rs11065987 | *BRAP* | 12 | 112,072,424 | A/G | HDL | 0.022 | 0.004 | 1.23E-09 | 187,119 |  | -0.042 | 0.042 | 0.317 | 12,928 | 2.15E-04 | 40.2 |
| rs2241210 | *UBE3B* | 12 | 109,950,144 | G/A | HDL | 0.033 | 0.004 | 2.49E-20 | 174,782 |  | -0.066 | 0.039 | 0.088 | 12,928 | 5.15E-04 | 90.0 |
| rs4379922 | *RP11-592O2.1* | 12 | 125,351,116 | C/T | HDL | 0.025 | 0.004 | 9.56E-12 | 186,203 |  | 0.020 | 0.041 | 0.628 | 12,928 | 2.53E-04 | 47.1 |
| rs838876 | *SCARB1* | 12 | 125,259,888 | A/G | HDL | 0.049 | 0.004 | 7.33E-33 | 173,066 |  | 0.037 | 0.045 | 0.411 | 12,928 | 9.22E-04 | 159.8 |
| rs4983559 | *ZBTB42* | 14 | 105,277,209 | G/A | HDL | 0.020 | 0.004 | 9.57E-09 | 183,672 |  | 0.051 | 0.043 | 0.228 | 12,928 | 1.63E-04 | 29.9 |
| rs10468017 | *ALDH1A2* | 15 | 58,678,512 | T/C | HDL | 0.118 | 0.004 | 1.21E-188 | 181,223 |  | -0.035 | 0.056 | 0.539 | 12,928 | 5.28E-03 | 962.6 |
| rs424346 | *ADAM10* | 15 | 59,010,962 | T/C | HDL | 0.068 | 0.011 | 4.84E-08 | 128,346 |  | 0.040 | 0.112 | 0.719 | 12,928 | 2.81E-04 | 36.1 |
| rs492571 | *FRMD5* | 15 | 44,211,273 | T/C | HDL | 0.066 | 0.009 | 1.27E-12 | 177,352 |  | 0.001 | 0.089 | 0.988 | 12,928 | 3.06E-04 | 54.3 |
| rs633695 | *LIPC* | 15 | 58,725,839 | G/A | HDL | 0.089 | 0.005 | 7.82E-58 | 92,820 |  | 0.078 | 0.042 | 0.062 | 12,928 | 2.89E-03 | 268.6 |
| rs16942887 | *PSKH1* | 16 | 67,928,042 | A/G | HDL | 0.083 | 0.005 | 8.28E-54 | 185,604 |  | 0.032 | 0.065 | 0.621 | 12,928 | 1.43E-03 | 265.5 |
| rs16965220 | *NLRC5* | 16 | 57,065,121 | A/C | HDL | 0.022 | 0.004 | 7.91E-09 | 185,512 |  | 0.058 | 0.040 | 0.151 | 12,928 | 1.89E-04 | 35.0 |
| rs2241770 | *NUP93* | 16 | 56,866,196 | T/C | HDL | 0.099 | 0.006 | 6.78E-60 | 185,537 |  | -0.018 | 0.066 | 0.784 | 12,928 | 1.62E-03 | 301.0 |
| rs2925979 | *CMIP* | 16 | 81,534,790 | C/T | HDL | 0.035 | 0.004 | 1.32E-19 | 185,553 |  | -0.069 | 0.042 | 0.099 | 12,928 | 4.85E-04 | 90.0 |
| rs9989419 | *AC012181.1* | 16 | 56,985,139 | G/A | HDL | 0.147 | 0.004 | 0.00E+00 | 177,532 |  | -0.133 | 0.065 | 0.041 | 12,928 | 9.34E-03 | 1674.2 |
| rs1877031 | *STARD3* | 17 | 37,814,080 | A/G | HDL | 0.034 | 0.004 | 1.20E-19 | 185,471 |  | 0.061 | 0.042 | 0.144 | 12,928 | 4.69E-04 | 87.1 |
| rs4148005 | *ABCA8* | 17 | 66,882,466 | T/G | HDL | 0.028 | 0.004 | 5.74E-14 | 184,431 |  | -0.069 | 0.044 | 0.123 | 12,928 | 3.35E-04 | 61.8 |
| rs4969178 | *PGS1* | 17 | 76,388,202 | G/A | HDL | 0.026 | 0.004 | 1.53E-12 | 185,426 |  | 0.032 | 0.042 | 0.446 | 12,928 | 3.04E-04 | 56.5 |
| rs4939883 | *LIPG* | 18 | 47,167,214 | C/T | HDL | 0.080 | 0.005 | 1.80E-66 | 185,576 |  | 0.032 | 0.056 | 0.572 | 12,928 | 1.70E-03 | 315.3 |
| rs6567160 | *U4* | 18 | 57,829,135 | T/C | HDL | 0.026 | 0.004 | 2.92E-09 | 185,608 |  | 0.062 | 0.047 | 0.186 | 12,928 | 2.12E-04 | 39.3 |
| rs103294 | *AC010518.3* | 19 | 54,797,848 | T/C | HDL | 0.052 | 0.004 | 3.995E-30 | 175,917 |  | 0.025 | 0.051 | 0.623 | 12,928 | 8.02E-04 | 141.3 |
| rs2278236 | *ANGPTL4* | 19 | 8,431,581 | A/G | HDL | 0.033 | 0.004 | 3.19E-18 | 185,450 |  | -0.052 | 0.038 | 0.177 | 12,928 | 4.82E-04 | 89.4 |
| rs2288912 | *APOC4* | 19 | 45,449,199 | G/C | HDL | 0.030 | 0.004 | 7.15E-15 | 178,910 |  | 0.017 | 0.044 | 0.689 | 12,928 | 3.80E-04 | 68.1 |
| rs737337 | *DOCK6* | 19 | 11,347,493 | T/C | HDL | 0.057 | 0.006 | 4.56E-17 | 185,432 |  | 0.066 | 0.072 | 0.356 | 12,928 | 4.62E-04 | 85.8 |
| rs4465830 | *ZNF335* | 20 | 44,585,420 | A/G | HDL | 0.060 | 0.004 | 5.18E-40 | 185,505 |  | -0.089 | 0.049 | 0.071 | 12,928 | 9.91E-04 | 184.1 |
| rs6031587 | *HNF4A* | 20 | 43,038,249 | C/T | HDL | 0.049 | 0.007 | 1.92E-09 | 163,095 |  | 0.078 | 0.082 | 0.344 | 12,928 | 2.67E-04 | 43.5 |
| rs181360 | *UBE2L3* | 22 | 21,928,916 | T/G | HDL | 0.038 | 0.004 | 9.24E-18 | 178,216 |  | -0.060 | 0.050 | 0.227 | 12,928 | 4.50E-04 | 80.1 |
| rs10903129 | *TMEM57* | 1 | 25,768,937 | G/A | LDL | 0.033 | 0.004 | 3.03E-17 | 169,920 |  | 0.004 | 0.039 | 0.914 | 12,928 | 4.62E-04 | 78.6 |
| rs12748152 | *AL034380.1* | 1 | 27,138,393 | T/C | LDL | 0.050 | 0.007 | 3.21E-12 | 172,988 |  | -0.121 | 0.083 | 0.145 | 12,928 | 3.30E-04 | 57.2 |
| rs2587534 | *RP4-781K5.7* | 1 | 234,849,339 | A/G | LDL | 0.039 | 0.004 | 8.06E-25 | 172,966 |  | -0.030 | 0.039 | 0.441 | 12,928 | 6.45E-04 | 111.7 |
| rs2642438 | *MARC1* | 1 | 220,970,028 | G/A | LDL | 0.035 | 0.004 | 7.32E-16 | 165,470 |  | -0.038 | 0.053 | 0.474 | 12,928 | 4.24E-04 | 70.2 |
| rs267733 | *ANXA9* | 1 | 150,958,836 | A/G | LDL | 0.033 | 0.005 | 5.29E-09 | 164,562 |  | 0.006 | 0.054 | 0.916 | 12,928 | 2.37E-04 | 39.0 |
| rs2902875 | *RP11-101C11.1* | 1 | 55,695,535 | T/C | LDL | 0.076 | 0.013 | 2.19E-09 | 89,883 |  | -0.193 | 0.133 | 0.145 | 12,928 | 4.00E-04 | 36.0 |
| rs4970712 | *EVI5* | 1 | 92,993,547 | C/A | LDL | 0.034 | 0.004 | 2.46E-13 | 173,036 |  | -0.001 | 0.051 | 0.987 | 12,928 | 3.43E-04 | 59.4 |
| rs646776 | *PSRC1* | 1 | 109,818,530 | T/C | LDL | 0.160 | 0.004 | 1.63E-272 | 173,021 |  | -0.024 | 0.047 | 0.606 | 12,928 | 7.60E-03 | 1325.6 |
| rs7534572 | *DOCK7* | 1 | 62,999,675 | G/C | LDL | 0.041 | 0.006 | 1.29E-11 | 75,145 |  | 0.041 | 0.043 | 0.347 | 12,928 | 6.55E-04 | 49.2 |
| rs7551981 | *RP11-101C11.1* | 1 | 55,719,166 | T/G | LDL | 0.047 | 0.004 | 1.36E-33 | 173,021 |  | 0.001 | 0.039 | 0.971 | 12,928 | 8.91E-04 | 154.3 |
| rs10195252 | *COBLL1* | 2 | 165,513,091 | T/C | LDL | 0.024 | 0.004 | 3.81E-08 | 157,208 |  | 0.066 | 0.048 | 0.169 | 12,928 | 2.37E-04 | 37.2 |
| rs10490626 | *INSIG2* | 2 | 118,835,841 | G/A | LDL | 0.051 | 0.007 | 1.70E-12 | 173,044 |  | 0.024 | 0.075 | 0.749 | 12,928 | 3.13E-04 | 54.2 |
| rs11563251 | *UGT1A1* | 2 | 234,679,384 | T/C | LDL | 0.035 | 0.006 | 4.50E-08 | 172,855 |  | -0.100 | 0.072 | 0.167 | 12,928 | 1.79E-04 | 31.0 |
| rs1250229 | *AC012462.1* | 2 | 216,304,384 | C/T | LDL | 0.024 | 0.004 | 3.13E-08 | 173,032 |  | 0.011 | 0.050 | 0.826 | 12,928 | 1.93E-04 | 33.5 |
| rs1367117 | *APOB* | 2 | 21,263,900 | A/G | LDL | 0.119 | 0.004 | 9.48E-183 | 173,007 |  | 0.054 | 0.059 | 0.366 | 12,928 | 5.06E-03 | 879.1 |
| rs2030746 | *AC073257.1* | 2 | 121,309,488 | T/C | LDL | 0.021 | 0.004 | 8.61E-09 | 173,024 |  | 0.034 | 0.039 | 0.384 | 12,928 | 1.83E-04 | 31.7 |
| rs2710642 | *EHBP1* | 2 | 63,149,557 | A/G | LDL | 0.024 | 0.004 | 6.09E-09 | 172,994 |  | -0.023 | 0.041 | 0.582 | 12,928 | 2.29E-04 | 39.6 |
| rs6544713 | *ABCG8* | 2 | 44,073,881 | T/C | LDL | 0.081 | 0.004 | 4.84E-83 | 172,940 |  | -0.015 | 0.042 | 0.724 | 12,928 | 2.23E-03 | 386.5 |
| rs6709904 | *ABCG8* | 2 | 44,080,324 | A/G | LDL | 0.055 | 0.009 | 4.58E-10 | 89,852 |  | -0.040 | 0.061 | 0.506 | 12,928 | 4.66E-04 | 41.9 |
| rs17404153 | *DNAJC13* | 3 | 132,163,200 | G/T | LDL | 0.034 | 0.005 | 1.83E-09 | 172,898 |  | -0.024 | 0.060 | 0.695 | 12,928 | 2.24E-04 | 38.7 |
| rs7640978 | *CMTM6* | 3 | 32,533,010 | C/T | LDL | 0.039 | 0.007 | 9.84E-09 | 172,228 |  | 0.080 | 0.075 | 0.284 | 12,928 | 1.87E-04 | 32.3 |
| rs9875338 | *GSTM5P1* | 3 | 12,296,469 | G/A | LDL | 0.027 | 0.004 | 2.21E-11 | 172,895 |  | 0.066 | 0.041 | 0.108 | 12,928 | 3.08E-04 | 53.2 |
| rs6818397 | *RGS12* | 4 | 3,434,885 | T/G | LDL | 0.022 | 0.004 | 1.68E-08 | 172,685 |  | -0.015 | 0.044 | 0.731 | 12,928 | 1.82E-04 | 31.4 |
| rs12916 | *HMGCR* | 5 | 74,656,539 | C/T | LDL | 0.073 | 0.004 | 7.79E-78 | 168,357 |  | -0.030 | 0.040 | 0.462 | 12,928 | 2.21E-03 | 372.1 |
| rs4530754 | *CSNK1G3* | 5 | 122,855,416 | A/G | LDL | 0.028 | 0.004 | 3.58E-12 | 173,003 |  | 0.028 | 0.039 | 0.483 | 12,928 | 3.37E-04 | 58.4 |
| rs6882076 | *TIMD4* | 5 | 156,390,297 | C/T | LDL | 0.046 | 0.004 | 3.31E-31 | 173,006 |  | 0.023 | 0.068 | 0.736 | 12,928 | 8.32E-04 | 144.0 |
| rs1408272 | *SLC17A3* | 6 | 25,842,951 | T/G | LDL | 0.052 | 0.008 | 3.68E-09 | 167,888 |  | 0.120 | 0.113 | 0.286 | 12,928 | 2.34E-04 | 39.3 |
| rs1564348 | *SLC22A1* | 6 | 160,578,860 | C/T | LDL | 0.048 | 0.005 | 2.76E-21 | 172,989 |  | -0.002 | 0.053 | 0.976 | 12,928 | 5.35E-04 | 92.5 |
| rs16891156 | *SLC22A2* | 6 | 160,608,804 | C/A | LDL | 0.097 | 0.017 | 8.23E-09 | 90,466 |  | 0.046 | 0.185 | 0.805 | 12,928 | 3.52E-04 | 31.8 |
| rs3757354 | *MYLIP* | 6 | 16,127,407 | C/T | LDL | 0.038 | 0.004 | 2.09E-17 | 172,987 |  | -0.092 | 0.047 | 0.051 | 12,928 | 4.36E-04 | 75.4 |
| rs6909746 | *FRK* | 6 | 116,352,750 | C/T | LDL | 0.026 | 0.004 | 7.86E-11 | 170,097 |  | 0.021 | 0.041 | 0.618 | 12,928 | 2.97E-04 | 50.5 |
| rs2073547 | *NPC1L1* | 7 | 44,582,331 | G/A | LDL | 0.049 | 0.005 | 1.92E-21 | 169,889 |  | 0.031 | 0.051 | 0.537 | 12,928 | 5.76E-04 | 98.0 |
| rs2390536 | *SP4* | 7 | 21,485,397 | A/G | LDL | 0.022 | 0.004 | 2.04E-08 | 172,981 |  | -0.015 | 0.040 | 0.713 | 12,928 | 1.99E-04 | 34.4 |
| rs4722551 | *MIR148A* | 7 | 25,991,826 | C/T | LDL | 0.039 | 0.005 | 3.95E-14 | 172,946 |  | -0.064 | 0.052 | 0.222 | 12,928 | 3.68E-04 | 63.7 |
| rs13277801 | *UBXN2B* | 8 | 59,353,534 | C/T | LDL | 0.034 | 0.004 | 3.99E-17 | 173,010 |  | 0.060 | 0.041 | 0.146 | 12,928 | 4.57E-04 | 79.1 |
| rs2737252 | *TRPS1* | 8 | 116,663,898 | G/A | LDL | 0.031 | 0.004 | 7.04E-14 | 172,950 |  | -0.050 | 0.046 | 0.281 | 12,928 | 3.39E-04 | 58.7 |
| rs2954029 | *RP11-136O12.2* | 8 | 126,490,972 | A/T | LDL | 0.056 | 0.004 | 2.10E-50 | 172,963 |  | 0.010 | 0.040 | 0.814 | 12,928 | 1.42E-03 | 245.4 |
| rs7832643 | *PLEC* | 8 | 145,022,657 | T/G | LDL | 0.034 | 0.004 | 2.67E-17 | 164,854 |  | -0.025 | 0.045 | 0.571 | 12,928 | 4.83E-04 | 79.6 |
| rs1883025 | *ABCA1* | 9 | 107,664,301 | C/T | LDL | 0.030 | 0.004 | 6.14E-11 | 172,330 |  | -0.005 | 0.045 | 0.917 | 12,928 | 2.63E-04 | 45.3 |
| rs3780181 | *VLDLR* | 9 | 2,640,759 | A/G | LDL | 0.045 | 0.007 | 1.76E-09 | 171,976 |  | -0.094 | 0.080 | 0.244 | 12,928 | 2.10E-04 | 36.2 |
| rs579459 | *ABO* | 9 | 136,154,168 | C/T | LDL | 0.067 | 0.005 | 2.42E-44 | 172,706 |  | -0.051 | 0.055 | 0.348 | 12,928 | 1.26E-03 | 218.4 |
| rs2419604 | *GPAM* | 10 | 113,944,271 | A/G | LDL | 0.030 | 0.004 | 7.49E-14 | 172,807 |  | 0.008 | 0.042 | 0.845 | 12,928 | 3.30E-04 | 57.0 |
| rs10832962 | *SPTY2D1* | 11 | 18,656,271 | T/C | LDL | 0.032 | 0.004 | 6.62E-14 | 172,920 |  | 0.075 | 0.068 | 0.273 | 12,928 | 3.70E-04 | 64.0 |
| rs10893499 | *ST3GAL4* | 11 | 126,241,979 | A/G | LDL | 0.052 | 0.005 | 3.86E-21 | 172,980 |  | -0.137 | 0.058 | 0.018 | 12,928 | 5.58E-04 | 96.6 |
| rs174583 | *FADS1* | 11 | 61,609,750 | C/T | LDL | 0.052 | 0.004 | 7.00E-41 | 172,982 |  | -0.014 | 0.042 | 0.745 | 12,928 | 1.09E-03 | 188.7 |
| rs964184 | *ZNF259* | 11 | 116,648,917 | G/C | LDL | 0.086 | 0.008 | 2.01E-26 | 89,866 |  | -0.040 | 0.059 | 0.504 | 12,928 | 1.34E-03 | 120.2 |
| rs1169288 | *HNF1A-AS1* | 12 | 121,416,650 | C/A | LDL | 0.038 | 0.004 | 6.45E-21 | 163,086 |  | -0.026 | 0.043 | 0.546 | 12,928 | 5.39E-04 | 87.9 |
| rs3184504 | *SH2B3* | 12 | 111,884,608 | C/T | LDL | 0.027 | 0.004 | 4.20E-12 | 164,996 |  | -0.011 | 0.042 | 0.791 | 12,928 | 3.01E-04 | 49.7 |
| rs4942486 | *BRCA2* | 13 | 32,953,388 | T/C | LDL | 0.024 | 0.004 | 2.26E-11 | 171,930 |  | -0.013 | 0.039 | 0.743 | 12,928 | 2.51E-04 | 43.1 |
| rs8017377 | *NYNRIN* | 14 | 24,883,887 | A/G | LDL | 0.030 | 0.004 | 2.52E-15 | 172,866 |  | 0.040 | 0.049 | 0.413 | 12,928 | 3.68E-04 | 63.6 |
| rs2000999 | *TXNL4B* | 16 | 72,108,093 | A/G | LDL | 0.065 | 0.005 | 4.22E-41 | 171,510 |  | 0.037 | 0.056 | 0.508 | 12,928 | 1.16E-03 | 199.7 |
| rs247616 | *AC012181.1* | 16 | 56,989,590 | C/T | LDL | 0.055 | 0.004 | 2.57E-37 | 171,458 |  | 0.017 | 0.044 | 0.694 | 12,928 | 1.04E-03 | 178.0 |
| rs2886232 | *ABCA10* | 17 | 67,150,176 | T/C | LDL | 0.045 | 0.006 | 3.88E-11 | 162,498 |  | 0.012 | 0.084 | 0.890 | 12,928 | 3.06E-04 | 49.7 |
| rs314253 | *ASGR1* | 17 | 7,091,650 | T/C | LDL | 0.024 | 0.004 | 3.44E-10 | 169,706 |  | 0.013 | 0.041 | 0.752 | 12,928 | 2.39E-04 | 40.6 |
| rs6504872 | *C17orf57* | 17 | 45,438,952 | T/C | LDL | 0.027 | 0.004 | 3.48E-13 | 171,519 |  | 0.023 | 0.045 | 0.613 | 12,928 | 3.20E-04 | 54.8 |
| rs2965157 | *CEACAM19* | 19 | 45,176,340 | T/C | LDL | 0.189 | 0.011 | 7.29E-62 | 170,260 |  | 0.233 | 0.143 | 0.103 | 12,928 | 1.66E-03 | 283.6 |
| rs6511720 | *LDLR* | 19 | 11,202,306 | G/T | LDL | 0.221 | 0.006 | 3.85E-262 | 170,608 |  | 0.084 | 0.060 | 0.161 | 12,928 | 7.63E-03 | 1311.4 |
| rs676388 | *MAMSTR* | 19 | 49,211,969 | C/T | LDL | 0.027 | 0.004 | 1.31E-11 | 166,830 |  | 0.008 | 0.043 | 0.854 | 12,928 | 2.77E-04 | 46.2 |
| rs7254892 | *PVRL2* | 19 | 45,389,596 | G/A | LDL | 0.485 | 0.012 | 0.00E+00 | 139,198 |  | 0.333 | 0.135 | 0.014 | 12,928 | 1.18E-02 | 1663.1 |
| rs1800961 | *HNF4A* | 20 | 43,042,364 | C/T | LDL | 0.069 | 0.011 | 6.03E-10 | 142,698 |  | 0.096 | 0.159 | 0.545 | 12,928 | 2.93E-04 | 41.8 |
| rs2328223 | *RP5-905G11.3* | 20 | 17,845,921 | C/A | LDL | 0.030 | 0.005 | 5.63E-09 | 170,762 |  | -0.006 | 0.049 | 0.910 | 12,928 | 2.09E-04 | 35.8 |
| rs364585 | *RP11-157E14.1* | 20 | 12,962,718 | G/A | LDL | 0.025 | 0.004 | 4.28E-10 | 171,526 |  | 0.008 | 0.040 | 0.834 | 12,928 | 2.50E-04 | 42.9 |
| rs6016373 | *SNORD112* | 20 | 39,154,095 | A/G | LDL | 0.035 | 0.004 | 7.95E-19 | 171,559 |  | 0.013 | 0.041 | 0.754 | 12,928 | 5.18E-04 | 89.0 |
| rs6065311 | *TOP1* | 20 | 39,724,338 | C/T | LDL | 0.042 | 0.004 | 1.66E-30 | 171,333 |  | 0.001 | 0.041 | 0.981 | 12,928 | 7.83E-04 | 134.2 |
| rs4253776 | *PPARA* | 22 | 46,629,479 | G/A | LDL | 0.031 | 0.006 | 3.35E-08 | 171,071 |  | 0.084 | 0.060 | 0.164 | 12,928 | 1.62E-04 | 27.8 |
| rs5763662 | *MTMR3* | 22 | 30,378,703 | T/C | LDL | 0.077 | 0.012 | 1.19E-08 | 162,777 |  | 0.119 | 0.138 | 0.386 | 12,928 | 2.47E-04 | 40.2 |
| rs11802413 | *TMEM57* | 1 | 25,760,920 | T/C | TC | 0.029 | 0.004 | 1.58E-14 | 187,138 |  | 0.003 | 0.041 | 0.936 | 12,928 | 3.59E-04 | 67.2 |
| rs2642438 | *MARC1* | 1 | 220,970,028 | G/A | TC | 0.037 | 0.004 | 1.28E-18 | 179,599 |  | -0.038 | 0.053 | 0.474 | 12,928 | 4.76E-04 | 85.6 |
| rs2902875 | *RP11-101C11.1* | 1 | 55,695,535 | T/C | TC | 0.071 | 0.012 | 1.79E-08 | 94,590 |  | -0.193 | 0.133 | 0.145 | 12,928 | 3.49E-04 | 33.0 |
| rs558971 | *RP4-781K5.7* | 1 | 234,853,406 | G/A | TC | 0.040 | 0.004 | 7.03E-28 | 187,254 |  | -0.034 | 0.039 | 0.387 | 12,928 | 6.52E-04 | 122.2 |
| rs646776 | *PSRC1* | 1 | 109,818,530 | T/C | TC | 0.127 | 0.004 | 4.77E-187 | 187,288 |  | -0.024 | 0.047 | 0.606 | 12,928 | 4.87E-03 | 917.2 |
| rs6603981 | *EVI5* | 1 | 92,993,807 | T/C | TC | 0.035 | 0.004 | 7.85E-15 | 187,329 |  | 0.000 | 0.089 | 0.999 | 12,928 | 3.56E-04 | 66.6 |
| rs7534572 | *DOCK7* | 1 | 62,999,675 | G/C | TC | 0.063 | 0.006 | 3.60E-28 | 83,151 |  | 0.041 | 0.043 | 0.347 | 12,928 | 1.57E-03 | 130.8 |
| rs7551981 | *RP11-101C11.1* | 1 | 55,719,166 | T/G | TC | 0.036 | 0.004 | 7.50E-22 | 187,282 |  | 0.001 | 0.039 | 0.971 | 12,928 | 5.00E-04 | 93.6 |
| rs11563251 | *UGT1A1* | 2 | 234,679,384 | T/C | TC | 0.037 | 0.006 | 1.27E-09 | 187,107 |  | -0.100 | 0.072 | 0.167 | 12,928 | 2.08E-04 | 38.9 |
| rs11694172 | *FAM117B* | 2 | 203,532,304 | G/A | TC | 0.028 | 0.004 | 1.95E-09 | 187,092 |  | -0.007 | 0.047 | 0.875 | 12,928 | 2.44E-04 | 45.6 |
| rs17526895 | *INSIG2* | 2 | 118,815,958 | A/G | TC | 0.042 | 0.007 | 5.777E-09 | 184,199 |  | 0.029 | 0.078 | 0.708 | 12,928 | 2.13E-04 | 39.3 |
| rs2030746 | *AC073257.1* | 2 | 121,309,488 | T/C | TC | 0.020 | 0.004 | 3.60E-08 | 187,289 |  | 0.034 | 0.039 | 0.384 | 12,928 | 1.54E-04 | 28.9 |
| rs2287623 | *ABCB11* | 2 | 169,830,155 | G/A | TC | 0.027 | 0.004 | 4.09E-12 | 184,257 |  | 0.006 | 0.042 | 0.887 | 12,928 | 3.12E-04 | 57.5 |
| rs515135 | *APOB* | 2 | 21,286,057 | C/T | TC | 0.124 | 0.005 | 6.38E-151 | 187,291 |  | 0.022 | 0.052 | 0.671 | 12,928 | 3.85E-03 | 724.3 |
| rs6544713 | *ABCG8* | 2 | 44,073,881 | T/C | TC | 0.077 | 0.004 | 1.69E-81 | 187,199 |  | -0.015 | 0.042 | 0.724 | 12,928 | 1.99E-03 | 373.5 |
| rs6709904 | *ABCG8* | 2 | 44,080,324 | A/G | TC | 0.055 | 0.008 | 8.40E-10 | 94,558 |  | -0.040 | 0.061 | 0.506 | 12,928 | 4.56E-04 | 43.1 |
| rs780093 | *GCKR* | 2 | 27,742,603 | T/C | TC | 0.052 | 0.004 | 2.59E-42 | 186,446 |  | -0.032 | 0.039 | 0.422 | 12,928 | 1.10E-03 | 204.6 |
| rs9306897 | *AC012361.1* | 2 | 21,138,066 | T/C | TC | 0.049 | 0.004 | 7.52E-37 | 185,808 |  | 0.024 | 0.042 | 0.571 | 12,928 | 9.35E-04 | 174.0 |
| rs13315871 | *PXK* | 3 | 58,381,287 | G/A | TC | 0.036 | 0.006 | 3.48E-08 | 187,287 |  | 0.072 | 0.068 | 0.287 | 12,928 | 1.81E-04 | 33.9 |
| rs7616006 | *GSTM5P1* | 3 | 12,267,648 | A/G | TC | 0.032 | 0.004 | 8.41E-17 | 187,246 |  | 0.035 | 0.039 | 0.375 | 12,928 | 4.09E-04 | 76.6 |
| rs7640978 | *CMTM6* | 3 | 32,533,010 | C/T | TC | 0.038 | 0.007 | 1.66E-08 | 186,485 |  | 0.080 | 0.075 | 0.284 | 12,928 | 1.74E-04 | 32.5 |
| rs6818397 | *RGS12* | 4 | 3,434,885 | T/G | TC | 0.025 | 0.004 | 9.51E-11 | 186,903 |  | -0.015 | 0.044 | 0.731 | 12,928 | 2.27E-04 | 42.4 |
| rs12916 | *HMGCR* | 5 | 74,656,539 | C/T | TC | 0.068 | 0.004 | 4.55E-74 | 182,530 |  | -0.030 | 0.040 | 0.462 | 12,928 | 1.97E-03 | 361.0 |
| rs4530754 | *CSNK1G3* | 5 | 122,855,416 | A/G | TC | 0.023 | 0.004 | 1.68E-09 | 187,272 |  | 0.028 | 0.039 | 0.483 | 12,928 | 2.27E-04 | 42.4 |
| rs6882076 | *TIMD4* | 5 | 156,390,297 | C/T | TC | 0.051 | 0.004 | 5.35E-41 | 187,270 |  | 0.023 | 0.068 | 0.736 | 12,928 | 1.01E-03 | 188.5 |
| rs11153594 | *FRK* | 6 | 116,354,591 | C/T | TC | 0.029 | 0.004 | 1.27E-14 | 187,230 |  | 0.020 | 0.040 | 0.627 | 12,928 | 3.46E-04 | 64.9 |
| rs11753995 | *SLC22A1* | 6 | 160,575,366 | A/G | TC | 0.049 | 0.005 | 1.84E-23 | 187,264 |  | -0.017 | 0.053 | 0.743 | 12,928 | 5.54E-04 | 103.8 |
| rs1800562 | *HFE* | 6 | 26,093,141 | G/A | TC | 0.057 | 0.008 | 1.91E-12 | 185,469 |  | 0.104 | 0.091 | 0.254 | 12,928 | 2.90E-04 | 53.8 |
| rs2814982 | *RP3-391O22.3* | 6 | 34,546,560 | C/T | TC | 0.044 | 0.006 | 3.68E-15 | 187,263 |  | 0.047 | 0.063 | 0.453 | 12,928 | 3.20E-04 | 59.9 |
| rs3757354 | *MYLIP* | 6 | 16,127,407 | C/T | TC | 0.035 | 0.004 | 2.22E-15 | 187,247 |  | -0.092 | 0.047 | 0.051 | 12,928 | 3.67E-04 | 68.7 |
| rs9376090 | *HBS1L* | 6 | 135,411,228 | T/C | TC | 0.025 | 0.004 | 2.60E-09 | 187,263 |  | -0.025 | 0.046 | 0.592 | 12,928 | 2.15E-04 | 40.3 |
| rs12670798 | *DNAH11* | 7 | 21,607,352 | C/T | TC | 0.036 | 0.004 | 9.48E-17 | 187,287 |  | -0.077 | 0.048 | 0.109 | 12,928 | 4.21E-04 | 78.8 |
| rs1997243 | *C7orf50* | 7 | 1,083,777 | G/A | TC | 0.033 | 0.005 | 2.72E-10 | 183,314 |  | 0.145 | 0.071 | 0.041 | 12,928 | 2.40E-04 | 44.1 |
| rs2073547 | *NPC1L1* | 7 | 44,582,331 | G/A | TC | 0.046 | 0.005 | 3.83E-21 | 184,098 |  | 0.031 | 0.051 | 0.537 | 12,928 | 5.11E-04 | 94.1 |
| rs10088180 | *AACP* | 8 | 18,228,116 | A/G | TC | 0.023 | 0.004 | 6.02E-10 | 187,142 |  | -0.006 | 0.046 | 0.900 | 12,928 | 1.74E-04 | 32.5 |
| rs2737252 | *TRPS1* | 8 | 116,663,898 | G/A | TC | 0.033 | 0.004 | 1.63E-16 | 187,202 |  | -0.050 | 0.046 | 0.281 | 12,928 | 3.85E-04 | 72.0 |
| rs2954029 | *RP11-136O12.2* | 8 | 126,490,972 | A/T | TC | 0.062 | 0.004 | 2.42E-65 | 187,216 |  | 0.010 | 0.040 | 0.814 | 12,928 | 1.68E-03 | 315.8 |
| rs7832643 | *PLEC* | 8 | 145,022,657 | T/G | TC | 0.029 | 0.004 | 3.12E-13 | 178,980 |  | -0.025 | 0.045 | 0.571 | 12,928 | 3.41E-04 | 61.0 |
| rs11789603 | *ABCA1* | 9 | 107,647,019 | T/C | TC | 0.043 | 0.006 | 1.44E-11 | 186,565 |  | -0.083 | 0.078 | 0.292 | 12,928 | 2.54E-04 | 47.4 |
| rs1883025 | *ABCA1* | 9 | 107,664,301 | C/T | TC | 0.067 | 0.004 | 5.75E-53 | 186,557 |  | -0.005 | 0.045 | 0.917 | 12,928 | 1.37E-03 | 255.2 |
| rs2066714 | *ABCA1* | 9 | 107,586,753 | C/T | TC | 0.044 | 0.008 | 1.14E-08 | 93,811 |  | 0.069 | 0.075 | 0.359 | 12,928 | 3.60E-04 | 33.8 |
| rs3780181 | *VLDLR* | 9 | 2,640,759 | A/G | TC | 0.044 | 0.007 | 6.67E-10 | 186,134 |  | -0.094 | 0.080 | 0.244 | 12,928 | 2.08E-04 | 38.8 |
| rs579459 | *ABO* | 9 | 136,154,168 | C/T | TC | 0.062 | 0.004 | 8.83E-42 | 186,925 |  | -0.051 | 0.055 | 0.348 | 12,928 | 1.06E-03 | 198.6 |
| rs581080 | *TTC39B* | 9 | 15,305,378 | C/G | TC | 0.038 | 0.005 | 1.02E-13 | 187,121 |  | 0.031 | 0.054 | 0.560 | 12,928 | 3.44E-04 | 64.3 |
| rs10900221 | *MARCH8* | 10 | 45,988,597 | A/G | TC | 0.026 | 0.004 | 7.96E-09 | 186,785 |  | -0.036 | 0.045 | 0.423 | 12,928 | 2.07E-04 | 38.7 |
| rs10904908 | *RP11-124N14.4* | 10 | 17,260,290 | G/A | TC | 0.025 | 0.004 | 2.60E-11 | 187,112 |  | -0.003 | 0.044 | 0.948 | 12,928 | 2.58E-04 | 48.2 |
| rs12412743 | *TECTB* | 10 | 114,045,333 | C/T | TC | 0.030 | 0.005 | 6.98E-10 | 187,282 |  | 0.037 | 0.053 | 0.483 | 12,928 | 2.15E-04 | 40.2 |
| rs2255141 | *GPAM* | 10 | 113,933,886 | A/G | TC | 0.031 | 0.004 | 6.51E-16 | 187,266 |  | 0.003 | 0.042 | 0.940 | 12,928 | 3.46E-04 | 64.8 |
| rs10832962 | *SPTY2D1* | 11 | 18,656,271 | T/C | TC | 0.032 | 0.004 | 1.54E-14 | 187,161 |  | 0.075 | 0.068 | 0.273 | 12,928 | 3.48E-04 | 65.2 |
| rs11220462 | *ST3GAL4* | 11 | 126,243,952 | A/G | TC | 0.047 | 0.006 | 5.49E-15 | 156,953 |  | -0.130 | 0.056 | 0.021 | 12,928 | 4.25E-04 | 66.8 |
| rs1535 | *FADS2* | 11 | 61,597,972 | A/G | TC | 0.050 | 0.004 | 8.62E-39 | 182,527 |  | -0.008 | 0.041 | 0.840 | 12,928 | 9.88E-04 | 180.4 |
| rs4752805 | *PTPRJ* | 11 | 48,018,355 | G/A | TC | 0.025 | 0.004 | 1.62E-09 | 187,233 |  | 0.009 | 0.047 | 0.848 | 12,928 | 2.00E-04 | 37.5 |
| rs964184 | *ZNF259* | 11 | 116,648,917 | G/C | TC | 0.121 | 0.008 | 2.84E-55 | 94,573 |  | -0.040 | 0.059 | 0.504 | 12,928 | 2.69E-03 | 255.2 |
| rs10773003 | *SBNO1* | 12 | 123,775,127 | A/G | TC | 0.037 | 0.006 | 4.08E-09 | 187,101 |  | 0.077 | 0.067 | 0.255 | 12,928 | 2.16E-04 | 40.5 |
| rs2244608 | *HNF1A-AS1* | 12 | 121,416,988 | G/A | TC | 0.031 | 0.004 | 9.62E-18 | 187,223 |  | -0.026 | 0.043 | 0.541 | 12,928 | 3.82E-04 | 71.6 |
| rs3184504 | *SH2B3* | 12 | 111,884,608 | C/T | TC | 0.032 | 0.004 | 1.62E-17 | 177,714 |  | -0.011 | 0.042 | 0.791 | 12,928 | 4.15E-04 | 73.9 |
| rs6573778 | *NYNRIN* | 14 | 24,872,209 | T/C | TC | 0.026 | 0.004 | 2.96E-11 | 187,079 |  | 0.023 | 0.051 | 0.644 | 12,928 | 2.43E-04 | 45.5 |
| rs10468017 | *ALDH1A2* | 15 | 58,678,512 | T/C | TC | 0.062 | 0.004 | 7.23E-48 | 181,378 |  | -0.035 | 0.056 | 0.539 | 12,928 | 1.31E-03 | 237.9 |
| rs633695 | *LIPC* | 15 | 58,725,839 | G/A | TC | 0.043 | 0.006 | 1.05E-14 | 93,067 |  | 0.078 | 0.042 | 0.062 | 12,928 | 5.98E-04 | 55.7 |
| rs2000999 | *TXNL4B* | 16 | 72,108,093 | A/G | TC | 0.062 | 0.004 | 6.80E-41 | 185,692 |  | 0.037 | 0.056 | 0.508 | 12,928 | 1.06E-03 | 196.6 |
| rs247616 | *AC012181.1* | 16 | 56,989,590 | T/C | TC | 0.050 | 0.004 | 4.47E-32 | 185,621 |  | -0.017 | 0.044 | 0.694 | 12,928 | 8.38E-04 | 155.6 |
| rs2886232 | *ABCA10* | 17 | 67,150,176 | T/C | TC | 0.036 | 0.006 | 3.87E-08 | 176,571 |  | 0.012 | 0.084 | 0.890 | 12,928 | 1.89E-04 | 33.3 |
| rs314253 | *ASGR1* | 17 | 7,091,650 | T/C | TC | 0.023 | 0.004 | 2.81E-10 | 183,868 |  | 0.013 | 0.041 | 0.752 | 12,928 | 2.16E-04 | 39.7 |
| rs6504872 | *C17orf57* | 17 | 45,438,952 | T/C | TC | 0.025 | 0.004 | 6.99E-12 | 185,712 |  | 0.023 | 0.045 | 0.613 | 12,928 | 2.75E-04 | 51.0 |
| rs2156552 | *LIPG* | 18 | 47,181,668 | T/A | TC | 0.057 | 0.005 | 1.25E-31 | 183,439 |  | 0.035 | 0.056 | 0.530 | 12,928 | 8.01E-04 | 147.1 |
| rs281393 | *RASIP1* | 19 | 49,224,484 | C/T | TC | 0.032 | 0.006 | 4.26E-08 | 93,067 |  | 0.021 | 0.049 | 0.674 | 12,928 | 3.68E-04 | 34.3 |
| rs6511720 | *LDLR* | 19 | 11,202,306 | G/T | TC | 0.185 | 0.006 | 5.43E-202 | 184,764 |  | 0.084 | 0.060 | 0.161 | 12,928 | 5.30E-03 | 984.2 |
| rs7412 | *APOE* | 19 | 45,412,079 | C/T | TC | 0.374 | 0.010 | 1.56E-283 | 92,046 |  | 0.253 | 0.091 | 0.005 | 12,928 | 1.62E-02 | 1514.5 |
| rs8103315 | *BCL3* | 19 | 45,254,168 | A/C | TC | 0.042 | 0.006 | 5.94E-15 | 156,370 |  | 0.051 | 0.079 | 0.519 | 12,928 | 3.76E-04 | 58.9 |
| rs1800961 | *HNF4A* | 20 | 43,042,364 | C/T | TC | 0.106 | 0.010 | 1.34E-24 | 156,406 |  | 0.096 | 0.159 | 0.545 | 12,928 | 7.06E-04 | 110.6 |
| rs2235367 | *ZHX3* | 20 | 39,830,122 | G/A | TC | 0.036 | 0.004 | 7.22E-25 | 185,691 |  | 0.000 | 0.038 | 0.998 | 12,928 | 5.60E-04 | 104.0 |
| rs2277862 | *FER1L4* | 20 | 34,152,782 | C/T | TC | 0.035 | 0.005 | 5.26E-11 | 185,738 |  | 0.006 | 0.054 | 0.914 | 12,928 | 2.42E-04 | 45.0 |
| rs6016373 | *SNORD112* | 20 | 39,154,095 | A/G | TC | 0.032 | 0.004 | 1.00E-17 | 185,730 |  | 0.013 | 0.041 | 0.754 | 12,928 | 4.23E-04 | 78.5 |
| rs138777 | *TOM1* | 22 | 35,711,098 | A/G | TC | 0.021 | 0.004 | 4.74E-08 | 185,274 |  | 0.025 | 0.041 | 0.534 | 12,928 | 1.81E-04 | 33.5 |
| rs181360 | *UBE2L3* | 22 | 21,928,916 | T/G | TC | 0.028 | 0.004 | 7.32E-10 | 178,322 |  | -0.060 | 0.050 | 0.227 | 12,928 | 2.34E-04 | 41.8 |
| rs4253772 | *PPARA* | 22 | 46,627,603 | T/C | TC | 0.032 | 0.006 | 9.85E-09 | 185,188 |  | 0.076 | 0.059 | 0.198 | 12,928 | 1.66E-04 | 30.8 |
| rs12748152 | *AL034380.1* | 1 | 27,138,393 | T/C | TG | 0.037 | 0.006 | 1.10E-09 | 177,762 |  | -0.121 | 0.083 | 0.145 | 12,928 | 2.24E-04 | 39.8 |
| rs1321257 | *GALNT2* | 1 | 230,305,312 | G/A | TG | 0.040 | 0.003 | 5.99E-31 | 177,758 |  | -0.035 | 0.042 | 0.401 | 12,928 | 7.86E-04 | 139.8 |
| rs17513135 | *RP11-15J6.1* | 1 | 40,035,686 | T/C | TG | 0.022 | 0.004 | 1.63E-08 | 174,742 |  | 0.023 | 0.047 | 0.624 | 12,928 | 1.82E-04 | 31.8 |
| rs4587594 | *DOCK7* | 1 | 63,133,930 | G/A | TG | 0.069 | 0.004 | 3.50E-82 | 177,772 |  | 0.051 | 0.049 | 0.301 | 12,928 | 2.21E-03 | 393.2 |
| rs1260326 | *GCKR* | 2 | 27,730,940 | T/C | TG | 0.115 | 0.003 | 2.29E-239 | 177,765 |  | -0.033 | 0.039 | 0.395 | 12,928 | 6.37E-03 | 1140.0 |
| rs13389219 | *COBLL1* | 2 | 165,528,876 | C/T | TG | 0.027 | 0.003 | 2.60E-15 | 177,783 |  | 0.056 | 0.048 | 0.248 | 12,928 | 3.57E-04 | 63.5 |
| rs2972146 | *AC068138.1* | 2 | 227,100,698 | T/G | TG | 0.028 | 0.003 | 2.97E-15 | 174,704 |  | -0.063 | 0.040 | 0.112 | 12,928 | 3.91E-04 | 68.3 |
| rs10440120 | *U6* | 3 | 12,486,964 | C/A | TG | 0.031 | 0.004 | 5.34E-11 | 174,886 |  | 0.011 | 0.055 | 0.836 | 12,928 | 2.76E-04 | 48.4 |
| rs442177 | *AFF1* | 4 | 88,030,261 | T/G | TG | 0.031 | 0.003 | 1.32E-18 | 177,798 |  | 0.028 | 0.040 | 0.483 | 12,928 | 4.93E-04 | 87.7 |
| rs6831256 | *DOK7* | 4 | 3,473,139 | G/A | TG | 0.026 | 0.004 | 1.60E-12 | 177,495 |  | -0.028 | 0.045 | 0.525 | 12,928 | 3.06E-04 | 54.3 |
| rs6882076 | *TIMD4* | 5 | 156,390,297 | C/T | TG | 0.029 | 0.004 | 1.51E-15 | 177,778 |  | 0.023 | 0.068 | 0.736 | 12,928 | 3.75E-04 | 66.8 |
| rs9686661 | *AC022431.2* | 5 | 55,861,786 | T/C | TG | 0.038 | 0.004 | 2.54E-16 | 177,050 |  | 0.104 | 0.092 | 0.261 | 12,928 | 4.19E-04 | 74.2 |
| rs2239520 | *PSORS1C1* | 6 | 31,088,922 | G/A | TG | 0.024 | 0.004 | 4.14E-10 | 151,047 |  | 0.059 | 0.039 | 0.135 | 12,928 | 2.69E-04 | 40.7 |
| rs2247056 | *XXbac-BPG248L24.13* | 6 | 31,265,490 | C/T | TG | 0.038 | 0.004 | 3.86E-21 | 174,062 |  | 0.057 | 0.047 | 0.219 | 12,928 | 5.39E-04 | 93.9 |
| rs2665357 | *SLC22A3* | 6 | 160,848,167 | C/A | TG | 0.021 | 0.003 | 8.33E-10 | 172,850 |  | 0.016 | 0.038 | 0.669 | 12,928 | 2.39E-04 | 41.3 |
| rs634869 | *RP11-12A2.3* | 6 | 139,831,757 | T/C | TG | 0.027 | 0.003 | 1.78E-14 | 177,755 |  | 0.000 | 0.044 | 0.998 | 12,928 | 3.82E-04 | 67.9 |
| rs719726 | *RP11-73O6.4* | 6 | 127,414,801 | T/C | TG | 0.020 | 0.004 | 2.49E-08 | 168,319 |  | -0.008 | 0.042 | 0.842 | 12,928 | 1.92E-04 | 32.3 |
| rs998584 | *VEGFA* | 6 | 43,757,896 | A/C | TG | 0.029 | 0.004 | 3.42E-15 | 174,573 |  | -0.040 | 0.045 | 0.370 | 12,928 | 3.59E-04 | 62.7 |
| rs287621 | *KLF14* | 7 | 130,435,181 | T/C | TG | 0.022 | 0.004 | 7.67E-09 | 177,813 |  | 0.021 | 0.044 | 0.644 | 12,928 | 2.02E-04 | 36.0 |
| rs38855 | *MET* | 7 | 116,358,044 | A/G | TG | 0.019 | 0.003 | 2.11E-08 | 177,825 |  | 0.071 | 0.048 | 0.142 | 12,928 | 1.81E-04 | 32.1 |
| rs4719841 | *MIR148A* | 7 | 25,997,536 | G/A | TG | 0.023 | 0.003 | 8.86E-11 | 177,775 |  | -0.016 | 0.039 | 0.680 | 12,928 | 2.62E-04 | 46.6 |
| rs12676857 | *NAT2* | 8 | 18,266,572 | C/T | TG | 0.033 | 0.005 | 7.29E-12 | 177,732 |  | 0.027 | 0.054 | 0.623 | 12,928 | 2.93E-04 | 52.1 |
| rs12678919 | *LPL* | 8 | 19,844,222 | A/G | TG | 0.170 | 0.006 | 1.82E-199 | 177,750 |  | -0.023 | 0.063 | 0.712 | 12,928 | 5.17E-03 | 923.7 |
| rs2954022 | *RP11-136O12.2* | 8 | 126,482,621 | C/A | TG | 0.078 | 0.003 | 2.23E-113 | 177,750 |  | 0.012 | 0.041 | 0.777 | 12,928 | 3.13E-03 | 558.7 |
| rs6995541 | *PINX1* | 8 | 10,671,260 | G/A | TG | 0.027 | 0.004 | 1.34E-12 | 177,486 |  | 0.105 | 0.048 | 0.029 | 12,928 | 2.89E-04 | 51.3 |
| rs10761762 | *JMJD1C* | 10 | 65,184,717 | T/C | TG | 0.027 | 0.003 | 1.06E-17 | 177,823 |  | -0.055 | 0.040 | 0.170 | 12,928 | 3.76E-04 | 66.9 |
| rs1832007 | *AKR1C4* | 10 | 5,254,847 | A/G | TG | 0.033 | 0.005 | 1.72E-12 | 177,504 |  | -0.026 | 0.055 | 0.635 | 12,928 | 2.73E-04 | 48.4 |
| rs2068888 | *CYP26A1* | 10 | 94,839,642 | G/A | TG | 0.024 | 0.003 | 1.68E-11 | 177,712 |  | 0.051 | 0.039 | 0.185 | 12,928 | 2.83E-04 | 50.2 |
| rs2250802 | *GPAM* | 10 | 113,921,354 | A/G | TG | 0.023 | 0.004 | 1.21E-10 | 174,734 |  | -0.002 | 0.044 | 0.973 | 12,928 | 2.21E-04 | 38.6 |
| rs10501321 | *MADD* | 11 | 47,294,626 | T/C | TG | 0.022 | 0.004 | 1.41E-08 | 177,680 |  | -0.013 | 0.043 | 0.758 | 12,928 | 2.14E-04 | 38.1 |
| rs10790162 | *BUD13* | 11 | 116,639,104 | A/G | TG | 0.231 | 0.007 | 1.10E-249 | 177,771 |  | -0.017 | 0.076 | 0.818 | 12,928 | 7.02E-03 | 1257.5 |
| rs11820504 | *AP000770.1* | 11 | 116,529,442 | C/T | TG | 0.060 | 0.004 | 1.10E-39 | 172,813 |  | 0.088 | 0.063 | 0.167 | 12,928 | 1.09E-03 | 188.4 |
| rs174535 | *RP11-467L20.9* | 11 | 61,551,356 | C/T | TG | 0.047 | 0.003 | 1.73E-41 | 177,773 |  | 0.005 | 0.041 | 0.902 | 12,928 | 1.07E-03 | 191.1 |
| rs11057408 | *ZNF664* | 12 | 124,464,836 | G/T | TG | 0.026 | 0.004 | 2.05E-12 | 174,454 |  | 0.014 | 0.055 | 0.795 | 12,928 | 3.11E-04 | 54.3 |
| rs16948098 | *FRMD5* | 15 | 44,219,607 | A/G | TG | 0.080 | 0.009 | 4.84E-17 | 163,328 |  | -0.006 | 0.088 | 0.946 | 12,928 | 4.94E-04 | 80.8 |
| rs2043085 | *ALDH1A2* | 15 | 58,680,954 | T/C | TG | 0.033 | 0.003 | 7.81E-20 | 176,147 |  | -0.015 | 0.041 | 0.722 | 12,928 | 5.25E-04 | 92.5 |
| rs588136 | *RP11-355N15.1* | 15 | 58,730,498 | C/T | TG | 0.050 | 0.004 | 3.37E-30 | 176,173 |  | 0.076 | 0.055 | 0.171 | 12,928 | 8.27E-04 | 145.8 |
| rs1800775 | *CETP* | 16 | 56,995,236 | C/A | TG | 0.040 | 0.004 | 1.33E-26 | 172,715 |  | 0.014 | 0.038 | 0.718 | 12,928 | 7.41E-04 | 128.0 |
| rs3198697 | *PDXDC1* | 16 | 15,129,940 | C/T | TG | 0.020 | 0.003 | 2.21E-08 | 175,934 |  | 0.005 | 0.039 | 0.907 | 12,928 | 1.93E-04 | 33.9 |
| rs749671 | *ZNF646* | 16 | 31,088,347 | G/A | TG | 0.021 | 0.003 | 6.11E-10 | 176,205 |  | -0.014 | 0.042 | 0.729 | 12,928 | 2.19E-04 | 38.5 |
| rs8077889 | *MPP3* | 17 | 41,878,166 | C/A | TG | 0.025 | 0.004 | 9.88E-09 | 176,194 |  | -0.045 | 0.048 | 0.351 | 12,928 | 2.04E-04 | 36.0 |
| rs3760627 | *APOC2* | 19 | 45,457,180 | C/T | TG | 0.019 | 0.003 | 5.29E-09 | 176,201 |  | 0.009 | 0.039 | 0.820 | 12,928 | 1.75E-04 | 30.9 |
| rs439401 | *APOE* | 19 | 45,414,451 | C/T | TG | 0.066 | 0.004 | 1.42E-66 | 152,584 |  | -0.046 | 0.040 | 0.250 | 12,928 | 1.97E-03 | 300.7 |
| rs7248104 | *INSR* | 19 | 7,224,431 | G/A | TG | 0.022 | 0.003 | 5.05E-10 | 176,083 |  | -0.020 | 0.040 | 0.611 | 12,928 | 2.42E-04 | 42.6 |
| rs4810479 | *PLTP* | 20 | 44,545,048 | C/T | TG | 0.047 | 0.004 | 2.07E-34 | 176,192 |  | 0.045 | 0.044 | 0.310 | 12,928 | 8.82E-04 | 155.6 |
| rs6029143 | *SNORD112* | 20 | 39,118,662 | C/T | TG | 0.039 | 0.007 | 4.93E-08 | 176,257 |  | -0.085 | 0.114 | 0.458 | 12,928 | 1.69E-04 | 29.9 |
| rs3761445 | *MAFF* | 22 | 38,595,411 | A/G | TG | 0.023 | 0.003 | 8.06E-12 | 175,846 |  | -0.002 | 0.049 | 0.974 | 12,928 | 2.65E-04 | 46.6 |

Supplementary Table S8. Summary information of instrumental variables for lipids and FTD in the European population. Note: SNP: the label of single-nucleotide polymorphism; CHR: chromosome; BP: base position; A1: effect allele; A2: alternative allele; BETA: SNP effect size; SE: standard error of the SNP effect size; PVE: proportion of variance explained by the SNP; *p*, *N*, and *F* represent *p* value, sample size, and *F* statistic, respectively. All the selected instruments collectively explain about 5.51% HDL variation, 6.48% LDL variation, 6.92% TC variation and 4.17% TG variation at the observed scale. For these instrumental variables, all the *F* statistics are above 10 (ranging from 29.9 to 1674.2 for HDL; ranging from 27.8 to 1663.1 for LDL; ranging from 28.9 to 1514.5 for TC and ranging from 29.9 to 1257.5 for TG) with an average *F* statistic of (122.3 for HDL; 159.1 for LDL; 141.2 for TC and 156.2 for TG) and an overall *F* statistic of (131.2 for HDL; 171.7 for LDL; 170.8 for TC and 161.8 for TG), suggesting that the selected genetic variants have sufficient strong effects for instrument and the bias of weak instrument is unlikely.

| SNP | GENE | CHR | BP | A1/A2 | lipids | | | | |  | ALS | | | | PVE | *F* |
| --- | --- | --- | --- | --- | --- | --- | --- | --- | --- | --- | --- | --- | --- | --- | --- | --- |
|  |  |  |  |  | Subtype | BETA | SE | *p* | N |  | BETA | SE | *p* | N |  |  |
| rs2144300 | *GALNT2* | 1 | 230,294,916 | C/T | HDL | 0.038 | 0.007 | 4.25E-08 | 70,657 |  | 0.068 | 0.064 | 0.286 | 4,084 | 4.22E-04 | 29.8 |
| rs6685271 | *TMED5* | 1 | 93,634,590 | C/A | HDL | -0.041 | 0.006 | 1.37E-10 | 70,657 |  | 0.007 | 0.050 | 0.889 | 4,084 | 5.88E-04 | 41.6 |
| rs117350179 | *PPARG* | 3 | 12,374,332 | C/G | HDL | -0.036 | 0.007 | 2.96E-08 | 70,657 |  | 0.039 | 0.055 | 0.472 | 4,084 | 4.33E-04 | 30.6 |
| rs1358980 | *VEGFA* | 6 | 43,764,551 | C/T | HDL | -0.033 | 0.006 | 2.69E-09 | 70,657 |  | -0.051 | 0.048 | 0.293 | 4,084 | 4.93E-04 | 34.9 |
| rs28366301 | *HLA-DRB1* | 6 | 32,560,883 | G/A | HDL | 0.039 | 0.006 | 7.58E-11 | 70,657 |  | -0.036 | 0.056 | 0.517 | 4,084 | 5.93E-04 | 41.9 |
| rs1026422 | *AC020743.4* | 7 | 50,319,807 | G/A | HDL | 0.034 | 0.005 | 4.84E-10 | 70,657 |  | -0.044 | 0.055 | 0.425 | 4,084 | 5.59E-04 | 39.5 |
| rs7778167 | *MIR129-1* | 7 | 127,851,628 | G/A | HDL | 0.058 | 0.009 | 1.99E-11 | 70,657 |  | -0.042 | 0.082 | 0.606 | 4,084 | 6.35E-04 | 44.9 |
| rs325 | *LPL* | 8 | 19,819,328 | T/C | HDL | 0.156 | 0.008 | 2.09E-79 | 70,657 |  | -0.052 | 0.088 | 0.562 | 4,084 | 4.96E-03 | 352.4 |
| rs3808447 | *TRPS1* | 8 | 116,575,459 | G/A | HDL | 0.051 | 0.007 | 3.64E-15 | 70,657 |  | -0.136 | 0.063 | 0.031 | 4,084 | 8.65E-04 | 61.2 |
| rs1883025 | *ABCA1* | 9 | 107,664,301 | C/T | HDL | -0.114 | 0.006 | 3.03E-76 | 70,657 |  | 0.079 | 0.060 | 0.188 | 4,084 | 4.73E-03 | 335.7 |
| rs7847628 | *PHF19* | 9 | 123,631,225 | A/G | HDL | -0.034 | 0.006 | 1.62E-08 | 70,657 |  | -0.075 | 0.049 | 0.126 | 4,084 | 4.46E-04 | 31.5 |
| rs2257129 | *RP11-159H3.1* | 10 | 122,898,697 | T/C | HDL | -0.038 | 0.006 | 1.85E-10 | 70,657 |  | -0.059 | 0.050 | 0.237 | 4,084 | 5.80E-04 | 41.0 |
| rs4917630 | *TECTB* | 10 | 114,019,830 | G/A | HDL | -0.041 | 0.006 | 7.56E-13 | 70,657 |  | 0.037 | 0.050 | 0.463 | 4,084 | 7.34E-04 | 51.9 |
| rs7895716 | *EXOC6* | 10 | 94,783,777 | C/G | HDL | 0.037 | 0.006 | 1.24E-10 | 70,657 |  | 0.003 | 0.061 | 0.961 | 4,084 | 5.83E-04 | 41.2 |
| rs11236520 | *CTD-2530H12.1* | 11 | 75,460,669 | G/A | HDL | -0.043 | 0.008 | 7.97E-09 | 70,657 |  | -0.132 | 0.076 | 0.083 | 4,084 | 4.68E-04 | 33.1 |
| rs3741297 | *ZNF259* | 11 | 116,657,667 | C/T | HDL | -0.271 | 0.010 | 2.52E-157 | 70,657 |  | -0.149 | 0.106 | 0.162 | 4,084 | 9.91E-03 | 706.9 |
| rs11067592 | *AC007623.1* | 12 | 110,069,190 | G/T | HDL | -0.093 | 0.011 | 5.12E-18 | 70,657 |  | 0.088 | 0.116 | 0.449 | 4,084 | 1.07E-03 | 75.4 |
| rs28577594 | *RILPL2* | 12 | 123,895,906 | G/C | HDL | -0.038 | 0.006 | 1.67E-09 | 70,657 |  | 0.053 | 0.052 | 0.313 | 4,084 | 5.09E-04 | 36.0 |
| rs67053123 | *RP11-592O2.1* | 12 | 125,353,810 | T/A | HDL | 0.074 | 0.006 | 1.38E-32 | 70,657 |  | -0.047 | 0.058 | 0.417 | 4,084 | 2.02E-03 | 143.2 |
| rs79105258 | *CUX2* | 12 | 111,718,231 | C/A | HDL | -0.130 | 0.007 | 1.69E-80 | 70,657 |  | -0.006 | 0.066 | 0.934 | 4,084 | 5.12E-03 | 363.3 |
| rs76213020 | *RP11-463J10.3* | 14 | 52,436,005 | A/C | HDL | 0.047 | 0.008 | 1.19E-08 | 70,657 |  | -0.071 | 0.069 | 0.302 | 4,084 | 4.63E-04 | 32.7 |
| rs2070895 | *LIPC* | 15 | 58,723,939 | G/A | HDL | 0.139 | 0.005 | 1.13E-140 | 70,657 |  | -0.017 | 0.050 | 0.739 | 4,084 | 9.06E-03 | 646.3 |
| rs2925979 | *CMIP* | 16 | 81,534,790 | T/C | HDL | 0.042 | 0.006 | 7.40E-13 | 70,657 |  | -0.083 | 0.050 | 0.094 | 4,084 | 7.37E-04 | 52.1 |
| rs56156922 | *AC012181.1* | 16 | 56,987,369 | T/C | HDL | 0.279 | 0.007 | 2.18E-371 | 70,657 |  | 0.000 | 0.066 | 0.996 | 4,084 | 2.38E-02 | 1722.2 |
| rs56303487 | *DPEP2* | 16 | 68,029,739 | C/T | HDL | 0.073 | 0.008 | 3.09E-19 | 70,657 |  | -0.031 | 0.072 | 0.664 | 4,084 | 1.14E-03 | 80.8 |
| rs11082764 | *LIPG* | 18 | 47,119,579 | A/G | HDL | 0.082 | 0.005 | 5.63E-51 | 70,657 |  | -0.050 | 0.049 | 0.312 | 4,084 | 3.21E-03 | 227.3 |
| rs429358 | *APOE* | 19 | 45,411,941 | T/C | HDL | -0.094 | 0.009 | 2.43E-24 | 70,657 |  | 0.022 | 0.082 | 0.785 | 4,084 | 1.46E-03 | 103.4 |
| rs76083992 | *PLTP* | 20 | 44,544,798 | C/T | HDL | -0.089 | 0.012 | 3.53E-14 | 70,657 |  | -0.082 | 0.100 | 0.412 | 4,084 | 8.16E-04 | 57.7 |
| rs235314 | *PTTG1IP* | 21 | 46,271,452 | C/T | HDL | -0.035 | 0.006 | 3.30E-10 | 70,657 |  | -0.005 | 0.049 | 0.911 | 4,084 | 5.55E-04 | 39.2 |
| rs7445 | *UBE2L3* | 22 | 21,977,047 | C/T | HDL | -0.043 | 0.006 | 1.25E-14 | 70,657 |  | -0.015 | 0.049 | 0.753 | 4,084 | 8.42E-04 | 59.6 |
| rs553427 | *RP4-781K5.7* | 1 | 234,852,760 | C/T | LDL | 0.039 | 0.006 | 1.46E-09 | 72,866 |  | 0.015 | 0.055 | 0.788 | 4,084 | 4.94E-04 | 36.0 |
| rs660240 | *CELSR2* | 1 | 109,817,838 | T/C | LDL | 0.125 | 0.011 | 1.79E-28 | 72,866 |  | 0.142 | 0.098 | 0.144 | 4,084 | 1.69E-03 | 123.4 |
| rs75352129 | *HS1BP3* | 2 | 20,848,192 | C/T | LDL | 0.233 | 0.014 | 5.64E-59 | 72,866 |  | 0.547 | 0.190 | 0.004 | 4,084 | 3.57E-03 | 261.2 |
| rs3846661 | *HMGCR* | 5 | 74,639,178 | A/G | LDL | 0.070 | 0.006 | 4.70E-31 | 72,866 |  | 0.092 | 0.048 | 0.054 | 4,084 | 1.81E-03 | 132.0 |
| rs6882076 | *TIMD4* | 5 | 156,390,297 | T/C | LDL | 0.048 | 0.008 | 1.05E-08 | 72,866 |  | 0.035 | 0.056 | 0.522 | 4,084 | 4.47E-04 | 32.6 |
| rs17145738 | *TBL2* | 7 | 72,982,874 | C/T | LDL | 0.056 | 0.009 | 4.83E-09 | 72,866 |  | 0.166 | 0.075 | 0.027 | 4,084 | 4.75E-04 | 34.6 |
| rs28537499 | *MIR148A* | 7 | 26,015,392 | A/C | LDL | -0.038 | 0.006 | 1.84E-10 | 72,866 |  | 0.028 | 0.049 | 0.571 | 4,084 | 5.61E-04 | 40.9 |
| rs2001846 | *TRIB1* | 8 | 126,478,450 | T/C | LDL | -0.034 | 0.006 | 6.34E-09 | 72,866 |  | -0.036 | 0.049 | 0.464 | 4,084 | 4.72E-04 | 34.4 |
| rs75214121 | *CYP7A1* | 8 | 59,398,276 | C/T | LDL | -0.037 | 0.006 | 8.88E-09 | 72,866 |  | -0.048 | 0.059 | 0.412 | 4,084 | 4.52E-04 | 33.0 |
| rs9411378 | *ABO* | 9 | 136,145,425 | C/A | LDL | 0.062 | 0.007 | 1.16E-19 | 72,866 |  | -0.056 | 0.060 | 0.346 | 4,084 | 1.13E-03 | 82.2 |
| rs41280378 | *OIT3* | 10 | 74,692,646 | T/G | LDL | -0.054 | 0.007 | 9.19E-15 | 72,866 |  | -0.074 | 0.053 | 0.162 | 4,084 | 8.13E-04 | 59.3 |
| rs113932726 | *ZNF259* | 11 | 116,650,638 | C/T | LDL | -0.061 | 0.011 | 1.70E-08 | 72,866 |  | -0.149 | 0.106 | 0.162 | 4,084 | 4.35E-04 | 31.7 |
| rs7315593 | *RPH3A* | 12 | 113,277,970 | G/T | LDL | -0.034 | 0.006 | 8.45E-09 | 72,866 |  | 0.013 | 0.049 | 0.795 | 4,084 | 4.63E-04 | 33.7 |
| rs75061399 | *NR1H4* | 12 | 100,942,077 | G/A | LDL | 0.038 | 0.007 | 4.80E-08 | 72,866 |  | -0.054 | 0.053 | 0.307 | 4,084 | 4.13E-04 | 30.1 |
| rs79105258 | *CUX2* | 12 | 111,718,231 | C/A | LDL | 0.066 | 0.007 | 2.84E-20 | 72,866 |  | -0.006 | 0.066 | 0.934 | 4,084 | 1.16E-03 | 84.6 |
| rs56903760 | *ALDH1A2* | 15 | 58,692,681 | C/T | LDL | 0.037 | 0.007 | 1.65E-08 | 72,866 |  | -0.042 | 0.052 | 0.417 | 4,084 | 4.40E-04 | 32.0 |
| rs77303550 | *TXNL4B* | 16 | 72,079,657 | C/T | LDL | -0.063 | 0.008 | 6.85E-16 | 72,866 |  | -0.135 | 0.056 | 0.017 | 4,084 | 8.90E-04 | 64.9 |
| rs3093679 | *TNFAIP1* | 17 | 26,664,215 | G/A | LDL | -0.048 | 0.008 | 1.92E-09 | 72,866 |  | -0.019 | 0.058 | 0.745 | 4,084 | 4.98E-04 | 36.3 |
| rs2738464 | *LDLR* | 19 | 11,242,307 | G/C | LDL | 0.083 | 0.006 | 3.84E-40 | 72,866 |  | 0.045 | 0.053 | 0.402 | 4,084 | 2.43E-03 | 177.5 |
| rs769446 | *APOE* | 19 | 45,408,628 | T/C | LDL | -0.571 | 0.015 | 0.00E+00 | 72,866 |  | 0.020 | 0.090 | 0.823 | 4,084 | 1.98E-02 | 1471.9 |
| rs56668103 | *ZHX3* | 20 | 39,826,079 | T/C | LDL | 0.038 | 0.007 | 7.28E-09 | 72,866 |  | 0.002 | 0.056 | 0.973 | 4,084 | 4.50E-04 | 32.8 |
| rs45576433 | *PCSK9* | 1 | 55,517,301 | A/G | TC | -0.056 | 0.006 | 1.46E-23 | 128,305 |  | -0.112 | 0.077 | 0.145 | 4,084 | 7.76E-04 | 99.7 |
| rs557933 | *RP4-781K5.7* | 1 | 234,853,268 | A/C | TC | 0.026 | 0.004 | 1.96E-11 | 128,305 |  | 0.016 | 0.055 | 0.775 | 4,084 | 3.54E-04 | 45.5 |
| rs61775910 | *DOCK7* | 1 | 62,993,403 | G/A | TC | -0.032 | 0.005 | 3.72E-10 | 128,305 |  | 0.093 | 0.061 | 0.127 | 4,084 | 3.08E-04 | 39.5 |
| rs646776 | *PSRC1* | 1 | 109,818,530 | C/T | TC | 0.074 | 0.007 | 4.53E-27 | 128,305 |  | 0.109 | 0.093 | 0.241 | 4,084 | 8.98E-04 | 115.3 |
| rs1260326 | *GCKR* | 2 | 27,730,940 | T/C | TC | -0.032 | 0.004 | 2.80E-19 | 128,305 |  | -0.004 | 0.048 | 0.930 | 4,084 | 6.38E-04 | 81.9 |
| rs12713468 | *EHBP1* | 2 | 62,983,213 | G/A | TC | -0.023 | 0.004 | 4.68E-08 | 128,305 |  | 0.005 | 0.052 | 0.923 | 4,084 | 2.29E-04 | 29.4 |
| rs75352129 | *HS1BP3* | 2 | 20,848,192 | C/T | TC | 0.119 | 0.009 | 2.01E-39 | 128,305 |  | 0.547 | 0.190 | 0.004 | 4,084 | 1.35E-03 | 173.5 |
| rs58143210 | *RGS12* | 4 | 3,425,030 | C/T | TC | -0.020 | 0.004 | 2.83E-08 | 128,305 |  | 0.069 | 0.047 | 0.150 | 4,084 | 2.44E-04 | 31.4 |
| rs11745603 | *TIMD4* | 5 | 156,394,518 | C/T | TC | 0.041 | 0.005 | 3.17E-16 | 128,305 |  | 0.033 | 0.056 | 0.552 | 4,084 | 5.26E-04 | 67.5 |
| rs11748027 | *ANKDD1B* | 5 | 74,909,972 | C/T | TC | 0.047 | 0.004 | 3.49E-36 | 128,305 |  | 0.096 | 0.048 | 0.045 | 4,084 | 1.21E-03 | 155.9 |
| rs55935382 | *AC020743.4* | 7 | 50,289,669 | C/A | TC | 0.021 | 0.004 | 7.73E-09 | 128,305 |  | -0.027 | 0.056 | 0.627 | 4,084 | 2.64E-04 | 33.9 |
| rs6970673 | *MIR148A* | 7 | 26,014,642 | C/T | TC | -0.021 | 0.004 | 1.12E-08 | 128,305 |  | 0.027 | 0.049 | 0.585 | 4,084 | 2.51E-04 | 32.2 |
| rs116961202 | *CYP7A1* | 8 | 59,382,311 | A/G | TC | -0.034 | 0.004 | 4.63E-18 | 128,305 |  | -0.043 | 0.058 | 0.464 | 4,084 | 5.72E-04 | 73.4 |
| rs2737252 | *TRPS1* | 8 | 116,663,898 | G/A | TC | -0.024 | 0.004 | 1.43E-09 | 128,305 |  | 0.026 | 0.053 | 0.622 | 4,084 | 2.85E-04 | 36.5 |
| rs6982502 | *RP11-136O12.2* | 8 | 126,479,362 | C/T | TC | -0.029 | 0.004 | 1.95E-16 | 128,305 |  | -0.041 | 0.049 | 0.408 | 4,084 | 5.28E-04 | 67.7 |
| rs2575876 | *ABCA1* | 9 | 107,665,739 | G/A | TC | -0.051 | 0.004 | 3.83E-38 | 128,305 |  | 0.055 | 0.061 | 0.362 | 4,084 | 1.27E-03 | 163.1 |
| rs9411378 | *ABO* | 9 | 136,145,425 | C/A | TC | 0.047 | 0.004 | 2.77E-28 | 128,305 |  | -0.056 | 0.060 | 0.346 | 4,084 | 9.65E-04 | 123.9 |
| rs57594838 | *TECTB* | 10 | 114,025,182 | T/G | TC | -0.024 | 0.004 | 5.94E-10 | 128,305 |  | 0.016 | 0.052 | 0.761 | 4,084 | 3.03E-04 | 38.8 |
| rs75542613 | *AP006216.5* | 11 | 116,679,155 | G/A | TC | -0.062 | 0.008 | 3.40E-15 | 128,305 |  | 0.028 | 0.105 | 0.792 | 4,084 | 4.84E-04 | 62.2 |
| rs97384 | *FADS1* | 11 | 61,624,181 | T/C | TC | 0.033 | 0.004 | 1.06E-16 | 128,305 |  | 0.063 | 0.050 | 0.208 | 4,084 | 5.33E-04 | 68.4 |
| rs2243616 | *HNF1A-AS1* | 12 | 121,406,370 | G/T | TC | -0.023 | 0.004 | 5.50E-10 | 128,305 |  | -0.080 | 0.049 | 0.104 | 4,084 | 3.04E-04 | 39.1 |
| rs67053123 | *RP11-592O2.1* | 12 | 125,353,810 | T/A | TC | 0.023 | 0.004 | 6.18E-09 | 128,305 |  | -0.047 | 0.058 | 0.417 | 4,084 | 2.66E-04 | 34.1 |
| rs1800588 | *LIPC* | 15 | 58,723,675 | C/T | TC | 0.054 | 0.004 | 4.25E-53 | 128,305 |  | 0.011 | 0.050 | 0.830 | 4,084 | 1.82E-03 | 234.3 |
| rs56156922 | *AC012181.1* | 16 | 56,987,369 | T/C | TC | 0.052 | 0.004 | 2.31E-32 | 128,305 |  | 0.000 | 0.066 | 0.996 | 4,084 | 1.10E-03 | 141.0 |
| rs77303550 | *TXNL4B* | 16 | 72,079,657 | C/T | TC | -0.055 | 0.005 | 2.64E-31 | 128,305 |  | -0.135 | 0.056 | 0.017 | 4,084 | 1.06E-03 | 136.3 |
| rs62074014 | *NPEPPS* | 17 | 45,668,509 | A/G | TC | -0.024 | 0.004 | 2.99E-09 | 128,305 |  | -0.027 | 0.050 | 0.582 | 4,084 | 2.70E-04 | 34.7 |
| rs3786247 | *LIPG* | 18 | 47,118,923 | T/G | TC | 0.040 | 0.004 | 5.27E-30 | 128,305 |  | -0.052 | 0.049 | 0.290 | 4,084 | 1.00E-03 | 129.0 |
| rs2738464 | *LDLR* | 19 | 11,242,307 | G/C | TC | 0.056 | 0.004 | 8.48E-49 | 128,305 |  | 0.045 | 0.053 | 0.402 | 4,084 | 1.67E-03 | 215.1 |
| rs34010237 | *MIR150* | 19 | 50,012,574 | G/A | TC | -0.033 | 0.005 | 6.40E-10 | 128,305 |  | 0.102 | 0.063 | 0.102 | 4,084 | 3.01E-04 | 38.7 |
| rs769446 | *APOE* | 19 | 45,408,628 | T/C | TC | -0.259 | 0.009 | 5.86E-187 | 128,305 |  | 0.020 | 0.090 | 0.823 | 4,084 | 6.53E-03 | 842.8 |
| rs56668103 | *ZHX3* | 20 | 39,826,079 | T/C | TC | 0.023 | 0.004 | 3.91E-09 | 128,305 |  | 0.002 | 0.056 | 0.973 | 4,084 | 2.66E-04 | 34.1 |
| rs2114273 | *Y_RNA* | 1 | 93,854,517 | T/C | TG | 0.023 | 0.004 | 5.95E-10 | 105,597 |  | 0.039 | 0.050 | 0.439 | 4,084 | 3.69E-04 | 39.0 |
| rs2144300 | *GALNT2* | 1 | 230,294,916 | C/T | TG | -0.025 | 0.004 | 4.23E-08 | 105,597 |  | 0.068 | 0.064 | 0.286 | 4,084 | 2.90E-04 | 30.7 |
| rs35529421 | *DOCK7* | 1 | 62,965,621 | T/A | TG | -0.062 | 0.005 | 3.57E-32 | 105,597 |  | 0.102 | 0.062 | 0.100 | 4,084 | 1.33E-03 | 141.0 |
| rs1260326 | *GCKR* | 2 | 27,730,940 | T/C | TG | -0.074 | 0.004 | 1.69E-94 | 105,597 |  | -0.004 | 0.048 | 0.930 | 4,084 | 4.02E-03 | 426.5 |
| rs12992267 | *RP11-116D2.1* | 2 | 21,215,645 | C/T | TG | 0.042 | 0.006 | 1.46E-11 | 105,597 |  | 0.102 | 0.090 | 0.257 | 4,084 | 4.26E-04 | 45.0 |
| rs35469118 | *RP11-476C8.3* | 4 | 88,168,178 | C/T | TG | 0.029 | 0.004 | 9.04E-15 | 105,597 |  | -0.035 | 0.052 | 0.488 | 4,084 | 5.82E-04 | 61.5 |
| rs3752442 | *HGFAC* | 4 | 3,446,883 | A/G | TG | -0.028 | 0.004 | 1.27E-14 | 105,597 |  | 0.020 | 0.048 | 0.673 | 4,084 | 5.74E-04 | 60.7 |
| rs154254 | *AC022431.2* | 5 | 55,820,584 | G/C | TG | 0.021 | 0.004 | 7.59E-09 | 105,597 |  | 0.045 | 0.049 | 0.358 | 4,084 | 3.13E-04 | 33.1 |
| rs2146324 | *VEGFA* | 6 | 43,756,863 | A/C | TG | 0.040 | 0.006 | 1.19E-10 | 105,597 |  | -0.031 | 0.063 | 0.625 | 4,084 | 3.97E-04 | 42.0 |
| rs12531645 | *MLXIPL* | 7 | 73,023,881 | G/A | TG | -0.088 | 0.006 | 3.23E-49 | 105,597 |  | 0.171 | 0.076 | 0.024 | 4,084 | 2.03E-03 | 215.1 |
| rs4389834 | *MIR148A* | 7 | 25,987,129 | G/A | TG | 0.022 | 0.004 | 2.28E-09 | 105,597 |  | -0.002 | 0.048 | 0.965 | 4,084 | 3.33E-04 | 35.2 |
| rs1059611 | *LPL* | 8 | 19,824,563 | T/C | TG | -0.141 | 0.005 | 1.49E-154 | 105,597 |  | -0.016 | 0.087 | 0.860 | 4,084 | 6.62E-03 | 703.9 |
| rs1495743 | *NAT2* | 8 | 18,273,300 | G/C | TG | -0.022 | 0.004 | 1.30E-08 | 105,597 |  | 0.012 | 0.048 | 0.795 | 4,084 | 3.05E-04 | 32.2 |
| rs2954021 | *RP11-136O12.2* | 8 | 126,482,077 | A/G | TG | -0.048 | 0.004 | 7.43E-42 | 105,597 |  | -0.042 | 0.049 | 0.392 | 4,084 | 1.71E-03 | 180.7 |
| rs4411227 | *CYP26A1* | 10 | 94,831,513 | C/G | TG | -0.031 | 0.004 | 3.57E-16 | 105,597 |  | 0.044 | 0.062 | 0.476 | 4,084 | 6.36E-04 | 67.2 |
| rs7916868 | *JMJD1C* | 10 | 64,988,931 | A/T | TG | -0.027 | 0.004 | 1.41E-10 | 105,597 |  | -0.016 | 0.049 | 0.745 | 4,084 | 3.94E-04 | 41.6 |
| rs174551 | *FADS2* | 11 | 61,573,684 | T/C | TG | 0.029 | 0.004 | 8.15E-15 | 105,597 |  | -0.038 | 0.051 | 0.455 | 4,084 | 5.79E-04 | 61.2 |
| rs651821 | *APOA5* | 11 | 116,662,579 | C/T | TG | -0.183 | 0.004 | 1.07E-537 | 105,597 |  | 0.010 | 0.056 | 0.853 | 4,084 | 2.33E-02 | 2518.2 |
| rs75766425 | *NID2* | 14 | 52,511,911 | G/C | TG | -0.031 | 0.006 | 1.27E-08 | 105,597 |  | -0.130 | 0.071 | 0.068 | 4,084 | 3.03E-04 | 32.0 |
| rs1800588 | *LIPC* | 15 | 58,723,675 | C/T | TG | 0.053 | 0.004 | 6.22E-51 | 105,597 |  | 0.011 | 0.050 | 0.830 | 4,084 | 2.09E-03 | 221.5 |
| rs2679617 | *C15orf50* | 15 | 70,207,077 | A/G | TG | -0.022 | 0.004 | 2.38E-08 | 105,597 |  | -0.013 | 0.051 | 0.794 | 4,084 | 2.90E-04 | 30.6 |
| rs56156922 | *AC012181.1* | 16 | 56,987,369 | T/C | TG | -0.031 | 0.004 | 1.41E-12 | 105,597 |  | 0.000 | 0.066 | 0.996 | 4,084 | 4.69E-04 | 49.5 |
| rs2278426 | *C19orf80* | 19 | 11,350,488 | C/T | TG | -0.030 | 0.004 | 1.62E-13 | 105,597 |  | 0.096 | 0.055 | 0.081 | 4,084 | 5.24E-04 | 55.3 |
| rs58542926 | *TM6SF2* | 19 | 19,379,549 | C/T | TG | -0.053 | 0.007 | 9.10E-14 | 105,597 |  | 0.032 | 0.100 | 0.750 | 4,084 | 5.21E-04 | 55.0 |
| rs75627662 | *APOE* | 19 | 45,413,576 | C/T | TG | 0.075 | 0.006 | 5.65E-42 | 105,597 |  | 0.035 | 0.064 | 0.584 | 4,084 | 1.73E-03 | 183.3 |
| rs76083992 | *PLTP* | 20 | 44,544,798 | C/T | TG | 0.058 | 0.008 | 1.33E-14 | 105,597 |  | -0.082 | 0.100 | 0.412 | 4,084 | 5.66E-04 | 59.8 |

Supplementary Table S9. Summary information of instrumental variables for lipids and ALS in the Asian population. Note: SNP: the label of single-nucleotide polymorphism; CHR: chromosome; BP: base position; A1: effect allele; A2: alternative allele; BETA: SNP effect size; SE: standard error of the SNP effect size; PVE: proportion of variance explained by the SNP; *p*, *N*, and *F* represent *p* value, sample size, and *F* statistic, respectively. BETA and SE of the index SNPs of HDL, LDL, TC and TG are recomputed by multiplying a factor of 1.05 (= 15.4/14.7), 1.11 (= 41.0/37.0), 0.91 (= 38.6/42.6) and 0.83 (= 71.9/86.8), respectively. All the selected instruments collectively explain about 7.78% HDL variation, 3.89% LDL variation, 2.66% TC variation and 5.07% TG variation at the observed scale. For these instrumental variables, all the *F* statistics are above 10 (ranging from 29.8 to 1722.2 for HDL; ranging from 30.1 to 1471.9 for LDL; ranging from 29.4 to 842.8 for TC and ranging from 30.6 to 2518.2 for TG) with an average *F* statistic of (185.2 for HDL; 136.4 for LDL; 110.3 for TC and 208.5 for TG) and an overall *F* statistic of (198.6 for HDL; 140.4 for LDL; 113.0 for TC and 216.9 for TG), suggesting that the selected genetic variants have sufficient strong effects for instrument and the bias of weak instrument is unlikely.

| SNP | OR (95% CI, *p*) |
| --- | --- |
| rs11125529 | 1.12 (0.93 ~ 1.33, 0.224) |
| rs10936599 | 1.14 (0.92 ~ 1.39, 0.240) |
| rs7675998 | 1.14 (0.94 ~ 1.37, 0.181) |
| rs2736100 | 1.09 (0.89 ~ 1.33, 0.383) |
| rs9420907 | 1.03 (0.85 ~ 1.23, 0.760) |
| rs8105767 | 1.10 (0.92 ~ 1.32, 0.308) |
| rs755017 | 1.11 (0.93 ~ 1.33, 0.236) |

Supplementary Table S10. Leave one out analysis of LTL on ALS in the European population (inverse variance weighting method).

| SNP | OR (95% CI, *p*) |
| --- | --- |
| rs11125529 | 0.89 (0.47 ~ 1.67, 0.721) |
| rs10936599 | 1.03 (0.47 ~ 2.33, 0.933) |
| rs7675998 | 0.81 (0.41 ~ 1.59, 0.531) |
| rs9420907 | 0.67 (0.35 ~ 1.28, 0.226) |
| rs8105767 | 0.79 (0.43 ~ 1.47, 0.454) |
| rs755017 | 0.79 (0.42 ~ 1.49, 0.465) |

Supplementary Table S11. Leave one out analysis of LTL on FTD in the European population (inverse variance weighting method).

| SNP | OR (95% CI, *p*) |
| --- | --- |
| rs3219104 | 0.79 (0.55 ~ 1.15, 0.222) |
| rs2293607 | 0.78 (0.52 ~ 1.15, 0.214) |
| rs10857352 | 0.72 (0.50 ~ 1.03, 0.075) |
| rs7705526 | 0.83 (0.56 ~ 1.22, 0.334) |
| rs7776744 | 0.73 (0.51 ~ 1.05, 0.090) |
| rs12415148 | 0.69 (0.47 ~ 1.01, 0.059) |
| rs41293836 | 0.78 (0.52 ~ 1.18, 0.240) |
| rs41309367 | 0.73 (0.51 ~ 1.04, 0.085) |

Supplementary Table S12. Leave one out analysis of LTL on ALS in the Asian population (inverse variance weighting method).

| Cohort | Nationality | Sample size | Male (%) | Mean age | Mean T/S |
| --- | --- | --- | --- | --- | --- |
| British Heart Foundation Family Heart Study (BHF-FHS) | UK | 1,487 | 80.10 | 60.80 (7.90) | 1.35 (0.22) |
| Estonian Genome Center, University of Tartu (EGCUT_370) | Estonia | 2,309 | 48.20 | 40.10 (16.20) | 1.85 (0.33) |
| Estonian Genome Center, University of Tartu (EGCUT_OMNI) | Estonia | 1,251 | 38.70 | 58.90 (22.20) | 1.69 (0.30) |
| Erasmus Rucphen Family (ERF) | Netherlands | 2,581 | 44.50 | 49.76 (14.87) | 1.78 (0.36) |
| The National FINRISK Study (FINRISK) | Finland | 520 | 47.40 | 52.20 (13.80) | 0.15 (0.19) |
| Nicotine Addiction Genetics-Finland study (FTC/NAG-FIN) | Finland | 1,054 | 61.90 | 54.90 (4.50) | 0.91 (0.16) |
| Helsinki Birth Cohort Study (HBCS) | Finland | 1,582 | 43.40 | 61.50 (2.90) | 1.39 (0.29) |
| Cooperative Health Research in the Region of Augsburg (KORA F3) | Germany | 1,636 | 49.60 | 62.20 (10.10) | 1.70 (0.28) |
| Cooperative Health Research in the Region of Augsburg (KORA F4) | Germany | 1,801 | 48.60 | 60.90 (8.90) | 1.80 (0.31) |
| Leiden Longevity Study (LLS) | Netherlands | 2,266 | 45.70 | 59.24 (6.80) | 1.46 (0.27) |
| Northern Finland Birth Cohort 1966 (NFBC1966) | Finland | 5,146 | 48.20 | 31.00 (0.00) | 1.22 (0.48) |
| Netherlands Twin Register (NTRMRG3) | Netherlands | 2,532 | 33.30 | 43.60 (14.70) | 2.67 (0.49) |
| Netherlands Twin Register (NTR_DETECT) | Netherlands | 158 | 52.50 | 18.70 (3.90) | 2.99 (0.38) |
| Netherlands Twin Register (NTR_GODOT) | Netherlands | 1,435 | 37.80 | 35.70 (10.60) | 2.83 (0.47) |
| Prevention of REnal and Vascular ENdstage Disease (PREVEND) | Netherlands | 2,926 | 51.00 | 48.00 (11.10) | 0.004 (0.28) |
| Queensland Institute of Medical Research (QIMR) | Australia | 2,371 | 49.10 | 24.00 (14.90) | 3.49 (0.61) |
| The TwinGene project (TWINGENE) | Sweden | 300 | 0.00 | 71.70 (5.90) | 1.43 (0.25) |
| The TwinsUK cohort (TWINSUK) | UK | 4,899 | 9.00 | 51.00 (13.40) | 3.71 (0.68) |
| United Kingdom Blood Service (UKBS) | UK | 1,430 | 48.40 | 43.40 (12.40) | 1.80 (0.50) |

Supplementary Table S13. Cohort demographics for studies included in the LTL GWAS meta-analysis in the European population[^1^](#_ENREF_1). Note: The value in parentheses is standard deviation. Mean telomere length was measured in each cohort using a quantitative PCR method and expressed it as a ratio of telomere repeat length to copy number of a single-copy gene (T/S ratio). T/S distributions were given from the primary data for each study prior to *Z*-transformation for analysis.

| Study | Ethnicity | Sample size | Male (%) | Mean age | Mean T/S |
| --- | --- | --- | --- | --- | --- |
| SCHS Discovery | Singaporean Chinese | 18,114 | 44.75 | 55.10 (7.43) | 1.02 (0.22) |
| SCHS Replication | Singaporean Chinese | 7,159 | 44.51 | 55.07 (7.44) | 1.01 (0.22) |

Supplementary Table S14. Main clinical characteristics of datasets in the LTL GWAS in the Asian population[^2^](#_ENREF_2). Note: The value in parentheses is standard deviation. T/S: relative telomere length, which determined as the ratio of telomere repeat length to copy number of a single-copy gene. In both GWAS phases, the sample sizes are slightly larger than those reported in the original paper[^2^](#_ENREF_2) (i.e. 18,114/7,159 vs. 16,759/6,337) due to quality control.

| Method | LTL-Asian & ALS-European | |  | LTL-Asian & FTD-European | |  | LTL-European & ALS-Asian | |
| --- | --- | --- | --- | --- | --- | --- | --- | --- |
|  | OR (95% CI, *p*) | *k*_1_/ *k*_0_ |  | OR (95% CI, *p*) | *k*_1_/ *k*_0_ |  | OR (95% CI, *p*) | *k*_1_/ *k*_0_ |
| IVW-random | 1.14 (0.99 ~ 1.31, 0.073) | 7/10 |  | 0.66 (0.34 ~ 1.31, 0.233) | 4/10 |  | 0.68 (0.38 ~ 1.23, 0.201) | 6/7 |
| IVW-fixed | 1.14 (0.99 ~ 1.31, 0.073) |  |  | 0.66 (0.34 ~ 1.31, 0.233) |  |  | 0.68 (0.38 ~ 1.23, 0.201) |  |
| MR-Egger | 0.79 (0.50 ~ 1.24, 0.299) |  |  | 1.45 (0.00 ~ 81357801.28, 0.937) |  |  | 0.38 (0.02 ~ 5.99, 0.451) |  |
| Weighted Median | 1.03 (0.86 ~ 1.23, 0.782) |  |  | 0.70 (0.31 ~ 1.61, 0.400) |  |  | 0.63 (0.31 ~ 1.32, 0.221) |  |
| Likelihood | 1.14 (0.99 ~ 1.31, 0.072) |  |  | 0.66 (0.33 ~ 1.30, 0.229) |  |  | 0.68 (0.38 ~ 1.23, 0.203) |  |

Supplementary Table S15. Association of LTL with the risk of ALS or FTD (using the European-instruments in the Asian population or using the Asian-instruments in the European population). Note: Here *k*_1_ is the final number of instruments employed in the analysis while *k*_0_ is the number of candidate instruments; the intercept of the MR-Egger regression is 0.031 (95% CI -0.006 ~ 0.068, *p* = 0.091), -0.050 (95% CI -1.172 ~ 1.073, *p* = 0.867) or 0.044 (95% CI -0.156 ~ 0.245, *p* = 0.637), respectively.


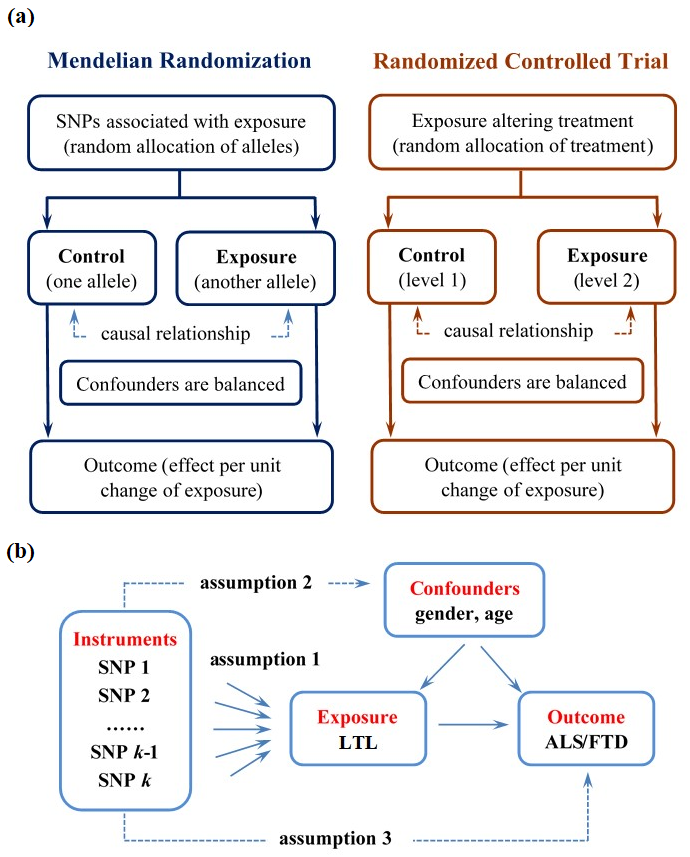


Supplementary Figure S1. (a) Similarities between Mendelian randomization and randomized controlled trial studies. (b) Illustration of three modeling assumptions of Mendelian randomization. Solid arrow or dotted arrow denotes the presence or absence of directional association. Valid Mendelian randomization requires selected SNPs to satisfy three assumptions. **Assumption 1**, known as the relevance assumption, assumes the selected SNPs are strongly associated with the exposure (i.e. LTL). **Assumption 2**, known as the independence assumption, assumes selected SNPs are not associated with the any known or known confounders of LTL and ALS/FTD. **Assumption 3**, known as the exclusion restriction assumption, assumes selected SNPs are independent of ALS/FTD conditional on LTL. SNP, single nucleotide polymorphisms; LTL: leukocyte telomere length; ALS, amyotrophic lateral sclerosis; FTD, frontotemporal dementia.


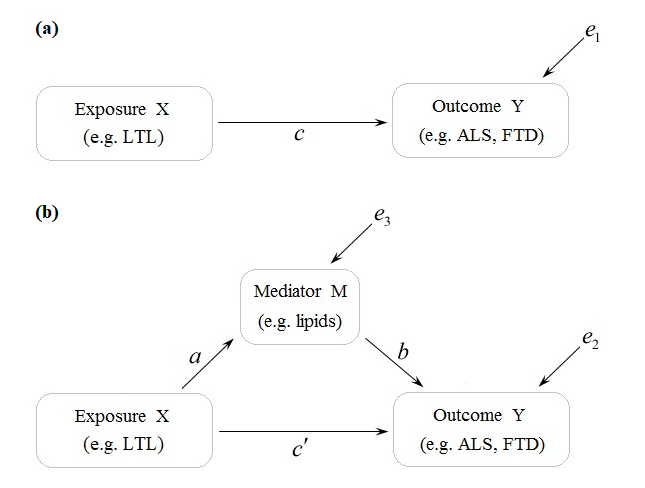


Supplementary Figure S2. Path diagram for the mediation model. Y is the outcome (e.g. ALS or FTD), X is the exposure (e.g. LTL), and M is the mediator (e.g. lipids), *e* is the residual. (a) Relationship between an exposure and an outcome; (b) Relationship between an exposure and an outcome while adjusting for a mediator.


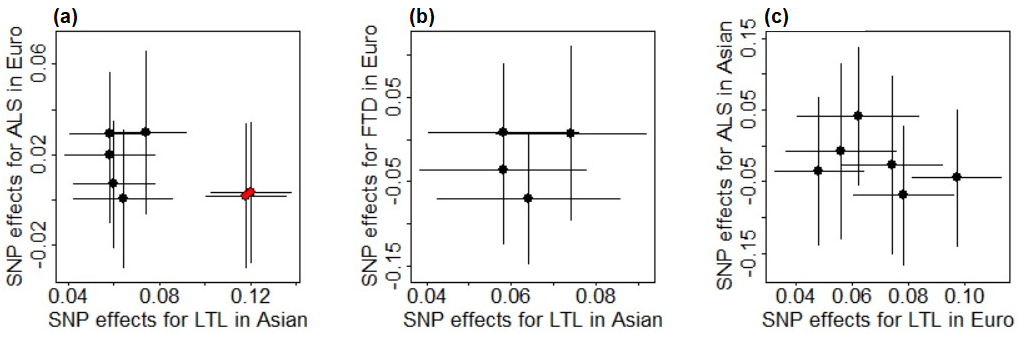


Supplementary Figure S3. Relationship between effect sizes on LTL and effect sizes on ALS/FTD for SNPs served as instrumental variables (using the European-instruments in Asians and using the Asian-instruments in Europeans). Results are shown for the seven SNPs on ALS (a) and the four SNPs on FTD (b) and the six SNPs on ALS (c). In each panel, horizontal/vertical lines represent the 95% confidence intervals. Two possible SNP outliers (i.e. rs2293607 on gene *TERC* and rs7705526 on gene *TERT*) are highlighted in red in the plot A.

**References**

1 Codd, V. *et al.* Identification of seven loci affecting mean telomere length and their association with disease. *Nat. Genet.* **45**, 422-427, 427e421-422 (2013).

2 Dorajoo, R. *et al.* Loci for human leukocyte telomere length in the Singaporean Chinese population and trans-ethnic genetic studies. *Nat Commun* **10**, 2491 (2019).
