# Supplementary material for: Mendelian randomization implies no direct causal association between leukocyte telomere length and amyotrophic lateral sclerosis
Source: Sci Rep. 2020 Jul 22;10:12184. doi: 10.1038/s41598-020-68848-9 (PMC7376149; doi:10.1038/s41598-020-68848-9)
Supplement: Supplementary file 3 — Supplementary Information 3. [file 41598_2020_68848_MOESM3_ESM.docx]

**Mendelian randomization implies no direct causal association between leukocyte telomere length and amyotrophic lateral sclerosis**

**Yixin Gao^1$^, Ting Wang^1$^, Xinghao Yu^1^, International FTD-Genomics Consortium (IFGC), Huashuo Zhao^1, 2*^, Ping Zeng^1, 2*^**

^1^ Department of Epidemiology and Biostatistics, School of Public Health, Xuzhou Medical University, Xuzhou, Jiangsu, 221004, PR China

^2^ Center for Medical Statistics and Data Analysis, School of Public Health, Xuzhou Medical University, Xuzhou, Jiangsu, 221004, PR China

^$^ Co-first authors

^*^ Corresponding authors: hszhao@xzhmu.edu.cn and [zpstat@xzhmu.edu.cn](mailto:zpstat@xzhmu.edu.cn)

**Supplementary Text**

# Description of GWAS Data Sets

**Leukocyte telomere length.** The genome-wide association study (GWAS) of leukocyte telomere length (LTL) was undertaken under the framework of European Network for Genetic and Genomic Epidemiology (ENGAGE) Project. A total of 37,684 individuals of European ancestry from 15 cohorts were included[^1^](#_ENREF_1) (Supplementary Table S13). Genotypes of single nucleotide polymorphism (SNP) were imputed with HapMap II CEU build 36 as reference panel. There were 2,362,330 SNPs left after quality control (SNP imputation accuracy < 0.50 and minor allele frequency [MAF] < 0.01). Linear additive regression was conducted in each cohort with the adjustment of age and gender as well as study-specific covariates (e.g. family and population structure)[^1^](#_ENREF_1). Finally, the meta-analysis was implemented for each genetic variant via the inverse variance-weighted method.

The Asian-LTL GWAS was first conducted within the Singapore Chinese Health Study (SCHS) study on 16,759 individuals in the Singaporean (Southern Han Chinese) population and later validated with additional 6,337 individuals[^2^](#_ENREF_2) (Supplementary Table S14). Genotypes of SNPs were imputed with IMPUTE v2 based on the 1000 Genomes Project Phase III[^3^](#_ENREF_3). After quality control (SNP genotype call rate < 0.95, MAF < 0.01, Hardy-Weinberg equilibrium [HWE] *p* value < 1.00E-6, impution information score < 0.8), 6,407,959 and 6,406,238 SNPs were available for the discovery and replication phases, respectively. Linear regression analysis was implemented for each SNP while controling for age, gender and the top three principal components of population stratification[^2^](#_ENREF_2). Finally, summary statistics from discovery and replication phases were combined using the inverse variance-weighted meta-analysis.

**Amyotrophic lateral sclerosis.** The GWAS of amyotrophic lateral sclerosis (ALS) used in the present study is the largest ALS Meta GWAS to date in the European population up on 80,610 individuals (20,806 cases and 59,804 controls)[^4^](#_ENREF_4). The imputation of SNP genotypes was conducted with minimac3[^5^](#_ENREF_5) using 1000 Genomes Project Phase III as reference panel[^3^](#_ENREF_3). Finally, 10,031,417 SNPs were left after quality control (SNP genotype call rate < 0.975, non-European ancestry, abnormal *F* inbreeding coefficient, mismatch between phenotypic and genotypic gender, cryptic relatedness defined as identity-by-descent proportion of inheritance > 0.125 and imputation accuracy < 0.30). Logistic regression was performed in each sub-study with principal components, age and gender as covariates. The association analysis was finally implemented using the fixed-effect weighted method across sub-studies.

The ALS GWAS in the Asian population was performed on 4,084 Chinese individuals (1,234 cases and 2,850 controls). The imputation of SNP genotypes was performed with IMPUTE[^6^](#_ENREF_6) using the 1000 Genomes Project Phase I as reference panel[^3^](#_ENREF_3). After quality control (SNP genotype call rate < 0.99, MAF < 0.01, HWE *p* value < 1.00E-6, differential missingness in genotypes between cases and controls with *p* value < 1.00E-6)[^7^](#_ENREF_7), 6,613,544 SNPs were left. Similarly, logistic regression was conducted for each SNP with the first five principal components as covariates.

**Frontotemporal dementia.** The GWAS of frontotemporal dementia (FTD) included 12,928 individuals (3,526 cases and 9,402 controls) of European ancestry. The SNP genotypes were imputed based on the 1000 Genomes Project[^3^](#_ENREF_3) via minimac and 6,026,385 SNPs were left for analysis after quality control (SNP genotype call rate < 0.95, NAF < 0.01, HWE *p* value < 1.00E-5, non-random missingness per SNP by case-control status/haplotype with *p* value < 1.00E-5 and imputation quality < 0.30)[^8^](#_ENREF_8). Logistic regression was performed with the first five eigenvectors from principal components analysis as covariates.

**Blood lipids.** We obtained summary statistics of four lipid traits from the Global Lipids Genetics Consortium (GLGC) study[^9^](#_ENREF_9), which was a meta-analysis that analyzed ~2.5 million genotyped and imputed SNPs on 188,577 individuals of European ancestry[^10^](#_ENREF_10). The GLGC study adjusted for the effect of gender, age (by incorporating age and age^2^) as well as population stratification[^9^](#_ENREF_9).

For the lipid traits in the Asian population, we obtained association summary statistics from the Japan biobank[^11^](#_ENREF_11), which analyzed about 128,000 individuals with a total of 5,961,600 SNPs. In the analysis the effects of gender, age, genetic ancestry, disease status and other trait-specific covariates were controlled whenever available.

# Model assumption of MR

Mendelian randomization (MR)[^12^](#_ENREF_12) is an instrumental variable statistical method to explore the causal relationship between an exposure (e.g. LTL) and an outcome (e.g. ALS or FTD) in observational studies with genetic variants (e.g. SNPs) as instruments. Each of selected SNPs should satisfy three model assumptions (Supplementary Fig. S1b)[^13^](#_ENREF_13),[^14^](#_ENREF_14): (**i**) the relevance assumption: SNP is strongly associated with exposure; (**ii**) the independence assumption: SNP is not associated with any other confounders which are potentially related to both exposure and outcome; (**iii**) the exclusion restriction assumption: SNP only influences outcome by the path of exposure alone and does not have horizontal pleiotropic effects.

Note that, while the first assumption (i.e. the relevance assumption) can be directly validated by examining the significance of SNPs on the exposure, the second two assumptions (i.e. the independence and exclusion restriction assumptions) are difficult to validate in practice. In the present we followed previous MR studies and performed extensive sensitivity analyses to validate the last two assumptions.

## Two-sample inverse variance weighted MR method

Let $\hat{\beta}_{i}^{\text{X}}$ and $\text{var}(\hat{\beta}_{i}^{\text{X}})$ be the effect size and variance of instrumental variable *i* for the exposure X; let the effect size and variance for the same instrumental variable *i* on the outcome Y be $\hat{\beta}_{i}^{\text{Y}}$ and $\text{var}(\hat{\beta}_{i}^{\text{Y}})$. The causal effect *θ_i_* for instrumental variable *i* is estimated by the Wald ratio method

$$\hat{\theta}_{i} = \hat{\beta}_{i}^{\text{Y}}/\hat{\beta}_{i}^{\text{X}}$$

Suppose both $\hat{\beta}_{i}^{\text{X}}$ and $\hat{\beta}_{i}^{\text{Y}}$ are normally distributed, the asymptotic variance of $\hat{\theta}_{i}$ is[^15^](#_ENREF_15),[^16^](#_ENREF_16)

$$\text{var}(\hat{\theta}_{i}) \approx\frac{\text{var}(\hat{\beta}_{i}^{\text{Y}})}{(\hat{\beta}_{i}^{\text{X}})^{2}} + \frac{(\hat{\beta}_{i}^{\text{Y}})^{2}\text{var}(\hat{\beta}_{i}^{\text{X}})}{(\hat{\beta}_{i}^{\text{X}})^{4}}$$

When there are *m* independent instrumental variables, one can combine the each Wald ratio estimate to generate a pooled causal effect through the inverse-variance weighted method[^17^](#_ENREF_17),[^18^](#_ENREF_18)

$$\hat{\theta} = \frac{1}{\sum_{i=1}^{m} \text{var}(\hat{\beta}_{i}^{\text{Y}})^{-1}(\hat{\beta}_{i}^{\text{X}})^{2}}\sum_{i=1}^{m} \text{var}(\hat{\beta}_{i}^{\text{Y}})^{-1}\hat{\beta}_{i}^{\text{Y}}\hat{\beta}_{i}^{\text{X}}$$

with the variance

$$\text{var}(\hat{\theta}) = \frac{1}{\sum_{i=1}^{m} \text{var}(\hat{\beta}_{i}^{\text{Y}})^{-1}(\hat{\beta}_{i}^{\text{X}})^{2}}$$

The causal effect estimate shown in is obtained by assuming no heterogeneity exists across instruments and is thus referred to as the fixed-effects IVW estimate. When the instrumental heterogeneity is observed, we account for such heterogeneity in the MR model by using the random-effects IVW estimate[^16^](#_ENREF_16),[^19^](#_ENREF_19),[^20^](#_ENREF_20), which allows for greater estimation uncertainty than the fixed-effects IVW estimate. The *Q* statistic[^19^](#_ENREF_19) is generally used to evaluate effect size heterogeneity.

## Mediation analysis with network MR analysis

The network MR analysis can be viewed as a special mediation analysis (Supplementary Fig. S2). First, assume that we want to study the relationship between the exposure variable X and the outcome variable Y, with *c* (equivalent to the causal effect *θ* given in ) denoted the total effect (Supplementary Fig. S2b)

$$\mathrm{Y}=i_{1}+c\text{X}+e_{1}$$

where *e*_1_ is the residual and represents the variation of Y that cannot be explained by X. The framework of the mediation model is shown in Supplementary Fig. S2b, representing the exposure variable X can directly influence the outcome variable Y or indirectly through the mediator (M)

$$\text{Y}=i_{2}+c'\text{X}+b\text{M}+e_{2}$$

$$\text{M}=i_{3}+a\text{X}+e_{3}$$

Network MR separately estimates the causal effect of the exposure on the mediator (i.e. *a*) and the causal effect of the mediator on the outcome (i.e. *b*) within the framework of two-sample IVW MR[^21^](#_ENREF_21). Then, the mediation effect (i.e. the indirect effect) is estimated with the product of the effects (i.e. *ab*).

**Test of the mediation effect**

Traditionally, the presence of a significant total effect (*c*) (Equation ) is the prerequisite for the mediation effect test[^22^](#_ENREF_22). However, the situation that the total effect is non-significant but a substantial mediation effect remains is not uncommon in practice, which is called suppression model[^23^](#_ENREF_23). Joint significance test is a variation of the mediation test, it ignores *c* and uses the significance of *a* and *b* to analyze the mediation effect[^23^](#_ENREF_23). Under this situation, we resort to an intuitive principle by separately testing *a* and *b*, and deem the mediation effect to be significant if *a* and *b* are simultaneously significant[^23-25^](#_ENREF_23).

Traditionally, we can use the Sobel test to assess the mediation effect[^22^](#_ENREF_22),[^26^](#_ENREF_26),[^27^](#_ENREF_27). However, the Sobel test is rather conservative and underpowered in practice[^23^](#_ENREF_23),[^28^](#_ENREF_28),[^29^](#_ENREF_29). Thus, we instead employ the Goodman test[^30^](#_ENREF_30). Specifically, let *S_a_* and *S_b_* be the standard errors of *a* and *b*, respectively; then the statistic of the Goodman test is defined as

$$u =\frac{ab}{S_{ab}}, S_{ab} = \sqrt{b^{2}S_{a}^{2} + a^{2}S_{b}^{2}-S_{a}^{2}S_{b}^{2}}$$

The null distribution of *u* is asymptotically a standard normal distribution. The Goodman test subtracts the third term in the denominator for an unbiased estimate of the variance of the mediated effect.

## Unify the unit of lipid levels in the European and Asian populations

In our main text we interpreted the causal effect of a continuous exposure (e.g. LTL or lipids) on ALS/FTD with odds ratio (OR) on the scale of one-unit change of the exposure. Because the effects of instruments were measured based on the standardized exposure, our OR estimates were also measured in terms of OR per one standard deviation (SD) change of the exposure. We referred to one SD of the exposure as one unit in our study.

During the network MR analysis, to make the estimated causal effects comparable between the European and Asian populations, following prior work[^31^](#_ENREF_31) we unified the unit of lipids in the two populations. Specifically, the SD of the four lipids in the European population[^32^](#_ENREF_32) was estimated to be 14.7 mg/dL, 37.0 mg/dL, 42.6 mg/dL or 86.8 mg/dL for high density lipoprotein (HDL), low density lipoprotein (LDL), total cholesterol (TC) and triglycerides (TG), respectively. The SD of the four lipids in the Asian population[^33^](#_ENREF_33) was estimated to be 15.4 mg/dL, 41.0 mg/dL, 38.6 mg/dL or 71.9 mg/dL for HDL, LDL, TC and TG, respectively. We applied the SD of lipids in the European population as the benchmark and transformed the information of lipids in the Asian population. We simply multiplied the marginal effect size and standard error of each instrument of a lipid trait by the ratio of SD in the Asian population and SD in the European population. For example, we recomputed the effect size and standard error of each instrument of LDL in the Asian lipid study by multiplying a factor of 1.11 (= 41.0/37.0).

Note that, the scale transformation does not influence the *p* value of the estimated causal effect, as the transformation changes the causal effect estimates and the SDs at the same scale. In our study we reported results based on the transformed summary statistics of blood lipids.

## Causal effect of LTL on ALS and FTD using the European-instruments in the Asian population or using the Asian-instruments in the European population

Fllowing the suggestion of one reviewer, we also made an additional exploration to estimate the causal effect of LTL on ALS/FTD using the European-instruments in the Asian population or using the Asian-instruments in the European population. With the fixed-effects IVW method, we again fail to detect statistically significant causal relationship (Supplementary Table S15) and the direction of the causality is similar to our main result shown in Table 4. However, a different association in direction and magnitude is found via the MR-Egger method. This may be due to the fact that MR-Egger method is developed based on a weaker assumption and is often less efficient compared with IVW and weighted median methods in causal inference. In addition, in terms of Supplementary Fig. S3a, when using the Asian-instruments of LTL to estimate causal association on ALS in the European population, two instruments (i.e. rs2293607 and rs7705526 with the large effect sizes of 0.120 and 0.118 on LTL in the Asian pouplation) seemed to be potential outliers and have substantial influence on the estimated causal effect in our MR analysis. Specifically, after removing them separately, the OR per SD decrease of LTL on ALS is 1.19 (95% CI: 1.00 ~ 1.41, *p* = 0.044) or 1.19 (95% CI: 1.01 ~ 1.40, *p* = 0.043); after removing them together, the OR per SD decrease of LTL on ALS is 1.31 (95% CI: 1.06 ~ 1.63, *p* = 0.013), which is supportive of the causal role of LTL on ALS in the European population.

Although the two sets of instruments of LTL share no common genetic variants between the two populations, we cannot exclude the probability that these index SNPs are transferable in the European and Asian populations. However, to ensure the validity of two-sample MR, one important assumption is that two sample sets should take from the same underlying population[^34^](#_ENREF_34). If such assumption is violated, MR may still provide evidence on whether a causal association exists but not necessarily on the precise magnitude of the causal effect[^35^](#_ENREF_35). Therefore, strictly speaking, the causative associations identified above are not definitive and need to be validated in future, if practical.

**References**

1 Codd, V. *et al.* Identification of seven loci affecting mean telomere length and their association with disease. *Nat. Genet.* **45**, 422-427, 427e421-422 (2013).

2 Dorajoo, R. *et al.* Loci for human leukocyte telomere length in the Singaporean Chinese population and trans-ethnic genetic studies. *Nat Commun* **10**, 2491 (2019).

3 1000 Genomes Project Consortium. An integrated map of genetic variation from 1,092 human genomes. *Nature* **491**, 56-65 (2012).

4 Nicolas, A. *et al.* Genome-wide Analyses Identify KIF5A as a Novel ALS Gene. *Neuron* **97** (2018).

5 Das, S. *et al.* Next-generation genotype imputation service and methods. *Nat. Genet.* **48**, 1284-1287 (2016).

6 Marchini, J. A new multipoint method for genome-wide association studies by imputation of genotypes. *Nat. Genet.* **39**, 906-913 (2007).

7 Benyamin, B. *et al.* Cross-ethnic meta-analysis identifies association of the GPX3-TNIP1 locus with amyotrophic lateral sclerosis. *Nat. Commun.* **8**, 611 (2017).

8 Ferrari, R. *et al.* Frontotemporal dementia and its subtypes: a genome-wide association study. *Lancet Neurol.* **13**, 686-699 (2014).

9 Willer, C. J. *et al.* Discovery and refinement of loci associated with lipid levels. *Nat. Genet.* **45**, 1274–1283 (2013).

10 Teslovich, T. M. *et al.* Biological, clinical and population relevance of 95 loci for blood lipids. *Nature* **466**, 707-713 (2010).

11 Kanai, M. *et al.* Genetic analysis of quantitative traits in the Japanese population links cell types to complex human diseases. *Nat. Genet.* **50**, 390-400 (2018).

12 Angrist, J. D., Imbens, G. W. & Rubin, D. B. Identification of Causal Effects Using Instrumental Variables. *J. Am. Stat. Assoc.* **91**, 444-455 (1996).

13 Lawlor, D. A., Harbord, R. M., Sterne, J. A., Timpson, N. & Davey Smith, G. Mendelian randomization: using genes as instruments for making causal inferences in epidemiology. *Stat. Med.* **27**, 1133-1163 (2008).

14 Sheehan, N. A., Didelez, V., Burton, P. R. & Tobin, M. D. Mendelian randomisation and causal inference in observational epidemiology. *PLoS Med.* **5**, e177 (2008).

15 Thomas, D. C., Lawlor, D. A. & Thompson, J. R. re: Estimation of bias in nongenetic observational studies using “Mendelian triangulation” by Bautista et al. *Ann. Epidemiol.* **17**, 511-513 (2007).

16 Burgess, S. & Thompson, S. G. Interpreting findings from Mendelian randomization using the MR-Egger method. *Eur. J. Epidemiol.* **32**, 377-389 (2017).

17 Burgess, S., Small, D. S. & Thompson, S. G. A review of instrumental variable estimators for Mendelian randomization. *Stat. Methods Med. Res.* **26**, 2333-2355 (2017).

18 Hartwig, F. P., Davey Smith, G. & Bowden, J. Robust inference in summary data Mendelian randomization via the zero modal pleiotropy assumption. *Int. J. Epidemiol.* **46**, 1985-1998 (2017).

19 Thompson, S. G. & Sharp, S. J. Explaining heterogeneity in meta-analysis: A comparison of methods. *Stat. Med.* **18**, 2693-2708 (1999).

20 Yavorska, O. O. & Burgess, S. MendelianRandomization: an R package for performing Mendelian randomization analyses using summarized data. *Int. J. Epidemiol.* **46**, 1734-1739 (2017).

21 Burgess, S., Daniel, R. M., Butterworth, A. S., Thompson, S. G. & Consortium, E. P.-I. Network Mendelian randomization: using genetic variants as instrumental variables to investigate mediation in causal pathways. *Int J Epidemiol* **44**, 484-495 (2015).

22 Baron, R. M. & Kenny, D. A. The moderator-mediator variable distinction in social psychological research: Conceptual, strategic, and statistical considerations. *J. Pers. Soc. Psychol.* **51**, 1173-1182 (1986).

23 MacKinnon, D. P., Lockwood, C. M., Hoffman, J. M., West, S. G. & Sheets, V. A comparison of methods to test mediation and other intervening variable effects. *Psychol. Methods* **7**, 83-104 (2002).

24 Fritz, M. S. & Mackinnon, D. P. Required sample size to detect the mediated effect. *Psychol. Sci.* **18**, 233-239 (2007).

25 Hayes, A. F. & Scharkow, M. The relative trustworthiness of inferential tests of the indirect effect in statistical mediation analysis: does method really matter? *Psychol. Sci.* **24**, 1918-1927 (2013).

26 Sobel, M. E. Asymptotic Confidence Intervals for Indirect Effects in Structural Equation Models. *Sociol. Methodol.* **13**, 290-312 (1982).

27 Mackinnon, D. P. & Dwyer, J. H. Estimating Mediated Effects in Prevention Studies. *Evaluation Rev* **17**, 144-158 (1993).

28 Barfield, R. *et al.* Testing for the indirect effect under the null for genome-wide mediation analyses. *Genet. Epidemiol.* **41**, 824-833 (2017).

29 Mackinnon, D. P., Warsi, G. & Dwyer, J. H. A Simulation Study of Mediated Effect Measures. *Multivariate Behav Res* **30**, 41-62 (1995).

30 Goodman, L. A. On the Exact Variance of Products. *Journal of the American Statistical Association* **55**, 708-713 (1960).

31 Zeng, P. & Zhou, X. Causal effects of blood lipids on amyotrophic lateral sclerosis: a Mendelian randomization study. *Hum. Mol. Genet.* **28**, 688-697 (2019).

32 Willer, C. J. *et al.* Discovery and refinement of loci associated with lipid levels. *Nat. Genet.* **45**, 1274-1283 (2013).

33 Kanai, M. *et al.* Genetic analysis of quantitative traits in the Japanese population links cell types to complex human diseases. *Nat. Genet.* **50**, 390-400 (2018).

34 Burgess, S., Scott, R. A., Timpson, N. J., Davey Smith, G. & Thompson, S. G. Using published data in Mendelian randomization: a blueprint for efficient identification of causal risk factors. *Eur. J. Epidemiol.* **30**, 543-552 (2015).

35 Haycock, P. C. *et al.* Best (but oft-forgotten) practices: the design, analysis, and interpretation of Mendelian randomization studies. *Am. J. Clin. Nutr.* **103**, 965-978 (2016).
